# Synthesis of ethoxy dibenzooxaphosphorin oxides through palladium-catalyzed C(sp^2^)–H activation/C–O formation

**DOI:** 10.3762/bjoc.10.120

**Published:** 2014-05-23

**Authors:** Seohyun Shin, Dongjin Kang, Woo Hyung Jeon, Phil Ho Lee

**Affiliations:** 1 Center for Catalytic Organic Reactions, National Creative Research Laboratory (NCRL), Chuncheon 200-701, Republic of Korea; 2 Department of Chemistry, Kangwon National University, Chuncheon 200-701, Republic of Korea

**Keywords:** C–H activation, catalysis, cyclization, palladium, phosphorus heterocyclic compound

## Abstract

We report an efficient Pd-catalyzed C(sp^2^)–H activation/C–O bond formation for the synthesis of ethoxy dibenzooxaphosphorin oxides from 2-(aryl)arylphosphonic acid monoethyl esters under aerobic conditions.

## Introduction

**Scheme 1:**
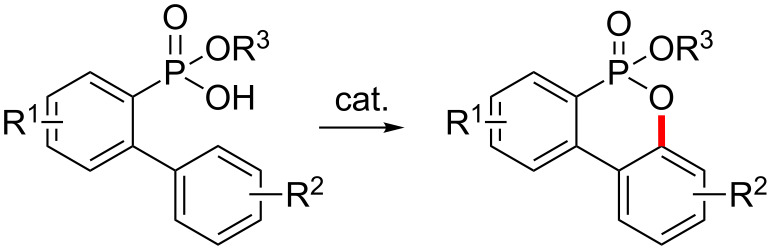
Synthesis of alkoxy dibenzooxaphosphorin oxides by C(sp^2^)–H activation/C–O formation.

Unreactive C(sp^2^)–H and C(sp^3^)–H bonds are ubiquitous in organic compounds [1–7], so that the development of methods for the transition metal-catalyzed C–H activation is one of the challenging goals in organic synthesis. Especially, the development of synthetic methods of C–heteroatom bond formation via C–H activation has received attention owing to the omnipresence of heterocyclic compounds in nature [8]. Recently, it has been demonstrated that the intramolecular bond formation between a heteroatom and a vicinal unreactive C–H is an efficient method for the synthesis of heterocycles [9–17]. Although C–H activation/C–N formation has been widely used for the synthesis of azaheterocycles, the preparation of oxaheterocycles via C–H activation/C–O formation has been described a lot less, because the energy correlation between the HOMO of the Pd–O bond and the LUMO of the Pd–C bond is unfavorable and the Pd–O bond has a significantly ionic character [18–23]. To expand this scope, we are interested in the development of C–H activation/C–O formation by means of new directing groups. Recently, a variety of C–H activations by using new phosphoryl-related directing groups have been reported by our [24–32] and other groups [33–41]. More recently, we developed a method allowing for synthetic access to benzoxaphosphole 1- and 2-oxides starting from phosphonic and phosphinic acids via Pd-catalyzed C(sp^2^ and sp^3^)–H activation/C–O formation [42]. In this context, we herein report the synthetic method of alkoxy dibenzooxaphosphorin oxides from 2-(aryl)arylphosphonic acid monoesters via Pd-catalyzed C(sp^2^)–H activation/C–O formation (Scheme 1).

## Results and Discussion

**Scheme 2:**
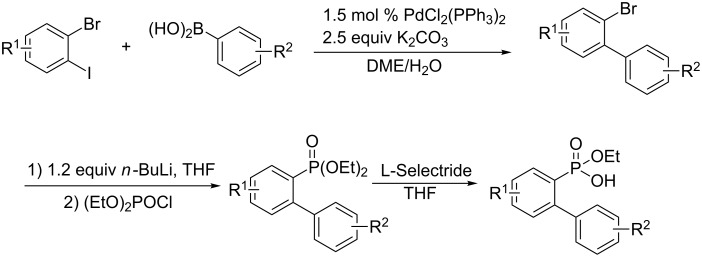
Preparation of 2-(aryl)arylphosphonic acid monoethyl esters.

First, a wide range of 2-(aryl)arylphosphonic acid monoethyl esters were efficiently prepared by a Suzuki reaction of 2-bromoiodoarenes with arylboronic acids, a lithium bromide exchange reaction of 2-bromobiaryls followed by diethylphosphinylation with diethyl chlorophosphate, and the C–O cleavage of diethyl 2-(aryl)arylphosphonates by using L-Selectride (Scheme 2).

**Scheme 3:**
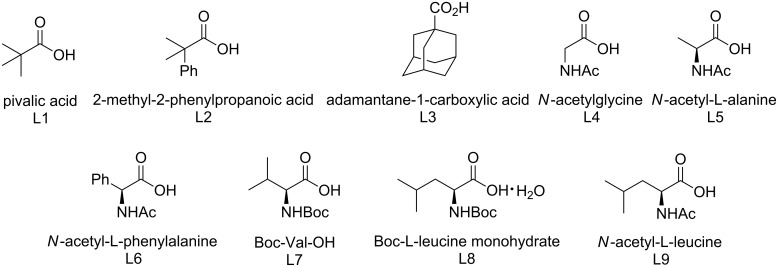
A variety of organic acids and monoprotected amino acids as ligands.

The C–H activation/C–O formation of 2-(phenyl)phenylphosphonic acid monoethyl ester (**1a**) was examined with a variety of oxidants and bases in the presence of Pd(OAc)_2_. A multitude of oxidants such as K_2_S_2_O_8_, BQ, benzoyl peroxide, PhI(TFA)_2_, Cu(OAc)_2_, CuCl_2_, CuBr, AgOAc, Ag_2_CO_3_ and Ag_2_O did not produce the cyclized product **2a** (see Supporting Information File 1). However, PhI(OAc)_2_, which is an efficient oxidant for the Pd(II)/Pd(IV) catalytic cycle, gave **2a** in 30% yield in *t*-butanol (80 °C for 16 h; Table 1, entry 1) [19,43–47]. In addition, various bases were examined. Although NaOAc, CsOAc, CsF and CsOPiv afforded **2a** in yields ranging from 42% to 52%, KOAc gave the best result (57%) in the presence of PhI(OAc)_2_ in *tert*-butanol (see Supporting Information File 1). *tert*-Butanol gave the best result among the solvents DCE, dioxane, ACN, *t*-AmOH, DMF, HFIP, THF, toluene, TFA and MeOH (see Supporting Information File 1). With this preliminary result in hand, we investigated a variety of organic acids as ligands in an effort to improve the catalytic efficiency (Scheme 3). However, these attempts provided no improvement (Table 1, entries 2–4). Finally, we discovered that easily accessible monoprotected amino acids, which have recently been established as efficient ligands in C–H activations [48–50], increased the yield (Table 1, entries 5–10). Among the investigated ligands, *N*-acetyl-*L*-leucine (L9) gave the best results (Table 1, entry 10). After examination of the reaction temperature (Table 1, entries 11–13) and time (Table 1, entries 14–16), the oxidative cyclization using PhI(OAc)_2_ (2 equiv) and KOAc (2 equiv) in the presence of Pd(OAc)_2_ (10 mol %) and L9 (30 mol %) gave the best result under aerobic conditions, affording **2a** in 61% yield (isolated yield 55%, Table 1, entry 16). Both Pd(TFA)_2_ and Pd(OTf)_2_∙H_2_O gave inferior results compared to Pd(OAc)_2_ (Table 1, entries 17 and 18).

**Table 1:** Optimization studies for the cyclization of 2-(phenyl)phenylphosphonic acid monoethyl esters.

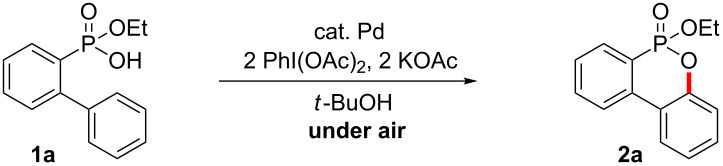					

entry	cat. Pd	ligand	*T* [°C]	*t* [h]	yield^a^ [%]

1	10 mol % Pd(OAc)_2_	—	80	16	30
2	10 mol % Pd(OAc)_2_	30 mol % L1	80	16	23
3	10 mol % Pd(OAc)_2_	30 mol % L2	80	16	34
4	10 mol % Pd(OAc)_2_	30 mol % L3	80	16	28
5	10 mol % Pd(OAc)_2_	30 mol % L4	80	16	48
6	10 mol % Pd(OAc)_2_	30 mol % L5	80	16	48
7	10 mol % Pd(OAc)_2_	30 mol % L6	80	16	54
8	10 mol % Pd(OAc)_2_	30 mol % L7	80	16	53
9	10 mol % Pd(OAc)_2_	30 mol % L8	80	16	51
10	10 mol % Pd(OAc)_2_	30 mol % L9	80	16	57
11	10 mol % Pd(OAc)_2_	30 mol % L9	60	16	20
12	10 mol % Pd(OAc)_2_	30 mol % L9	100	16	61
13	10 mol % Pd(OAc)_2_	30 mol % L9	120	16	50
14	10 mol % Pd(OAc)_2_	30 mol % L9	100	4	45
15	10 mol % Pd(OAc)_2_	30 mol % L9	100	8	51
**16**	**10 mol % Pd(OAc)_2_**	**30 mol % L9**	**100**	**12**	**61(55)**
17	10 mol % Pd(TFA)_2_	30 mol % L9	100	12	53
18	10 mol % Pd(OTf)_2_·H_2_O	30 mol % L9	100	12	45

^a^Yields were determined by ^1^H NMR with CH_2_Br_2_ as an internal standard. The number in parentheses is the isolated yield.

**Scheme 4:**
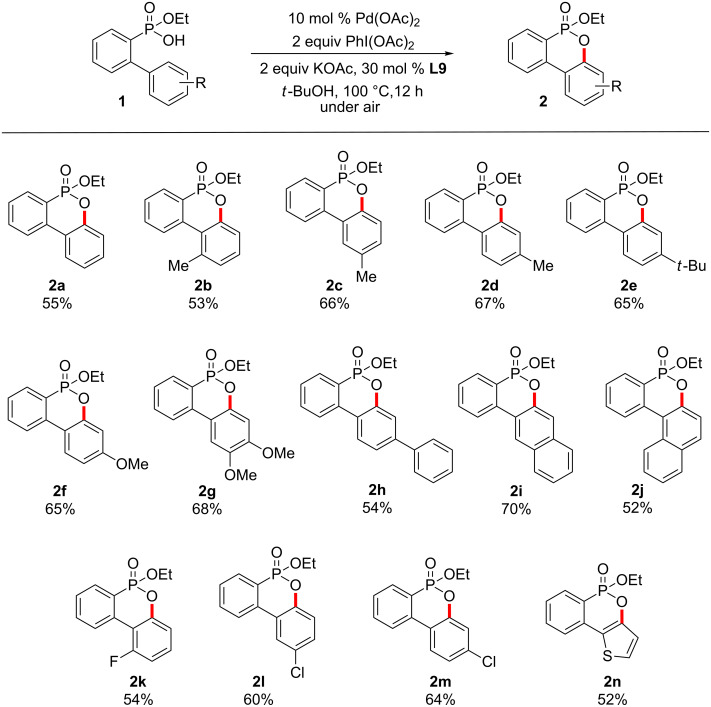
Cyclization of 2-arylphenylphosphonic acid monoethyl esters.

To ascertain the scope of the Pd-catalyzed C–H activation followed by the C–O formation, a wide range of 2-(aryl)phenylphosphonic acid monoethyl esters **1** were examined under the optimized reaction conditions (Scheme 4). Phenylphosphonic acid monoethyl ester **1b** with a 2-methyl group on the phenyl ring was transformed to the desired dibenzooxaphosphorin oxide **2b** in 53% yield. Phenylphosphonic acid monoethyl esters (**1c**) with a 3-methyl group were selectively converted to the cyclized products (**2c**) in 66% yield due to steric effects. In the case of 4-*tert*-butyl, the desired product **2e** was obtained in 65% yield. Substrate **1f**, characterized by an electron-donating 4-methoxy group, was cyclized to dibenzooxaphosphorin oxide **2f** in 65% yield under aerobic conditions. The present method worked equally well with 3,4-dimethoxyphenyl-substituted phenylphosphonic acid monoethyl ester **1g**. Phenylphosphonic acid monoethyl ester **1h** with a 4-phenyl group on the phenyl ring turned out to be compatible with the reaction conditions. As anticipated, 2-naphthyl-substituted phenylphosphonic acid monoethyl ester **1i** underwent the Pd-catalyzed oxidative cyclization regioselectively at the sterically less hindered position to afford the desired dibenzooxaphosphorin oxide **2i** in 70% yield. We were pleased to obtain **2j** by a Pd-catalyzed oxidative cyclization of 1-naphthyl-substituted phenylphosphonic acid monoethyl ester **1j**. 2-(Aryl)phenylphosphonic acid monoethyl esters **1k**, **1l** and **1m** with an electron-withdrawing fluoro or chloro group on the phenyl ring were subjected to the oxidative cyclization to deliver the desired products **2k**, **2l** and **2m** in yields ranging from 54% and 64%. In particular, the tolerance of the chloro groups may be of importance for a subsequent catalytic cross-coupling reaction. Substrate **1n**, which contains a 2-thiophenyl moiety, was subjected to the cyclization affording **2n** in 52% yield. The preparation of 2-arylphenylphosphonic acid monoethyl esters with a nitro, difluoro, or ethoxycarbonyl group failed.

**Scheme 5:**
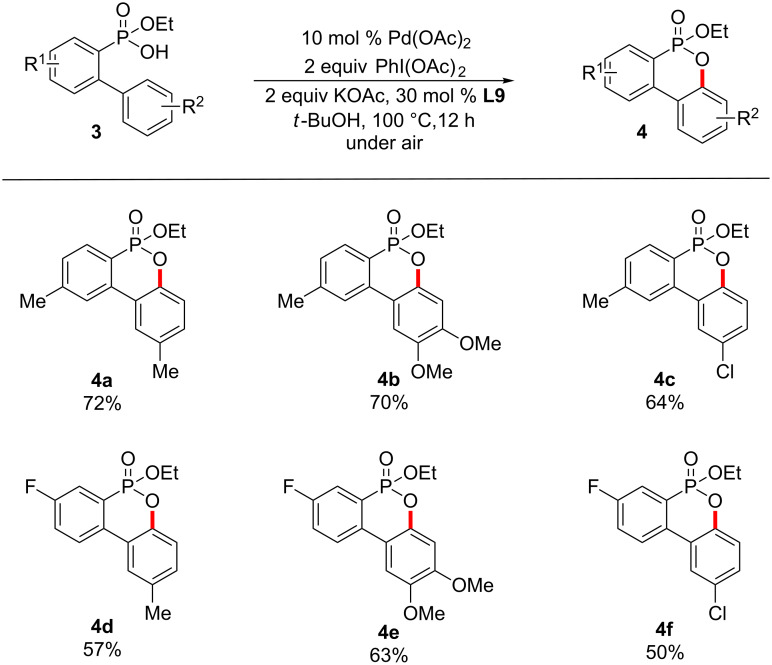
Cyclization of 2-(aryl)arylphosphonic acid monoethyl esters.

Next, the Pd-catalyzed oxidative cyclization of 2-(aryl)arylphosphonic acid monoethyl esters **3** were examined to demonstrate the efficiency of the present method (Scheme 5). 4-Methylphenylphosphonic acid monoethyl esters **3a** and **3b** with a 3-methyl- and 3,4-dimethoxyphenyl group at 2-position turned out to be compatible with the Pd-catalyzed oxidative cyclization. There are no regioisomers formed due to steric effects. Substrate **3c** bearing a chloro group was selectively cyclized to afford **4c** in 64% yield. To our delight, the present method worked equally well even if a fluoro group on the phenyl ring is present. 3-Fluorophenylphosphonic acid monoethyl esters **3d**, **3e** and **3f** with 3-methyl-, 3,4-dimethoxy and 3-chlorophenyl groups at the 2-position selectively underwent the oxidative cyclization to give the corresponding cyclized products **4d**, **4e** and **4f** in yields ranging from 50% and 63%.

**Scheme 6:**
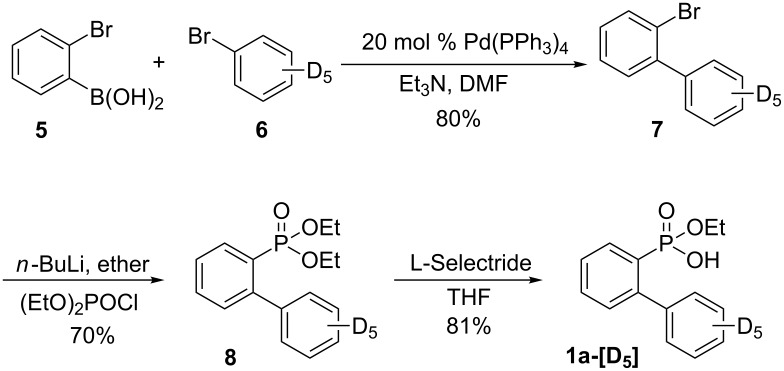
Preparation of **1a**-**[D_5_]**.

**Scheme 7:**
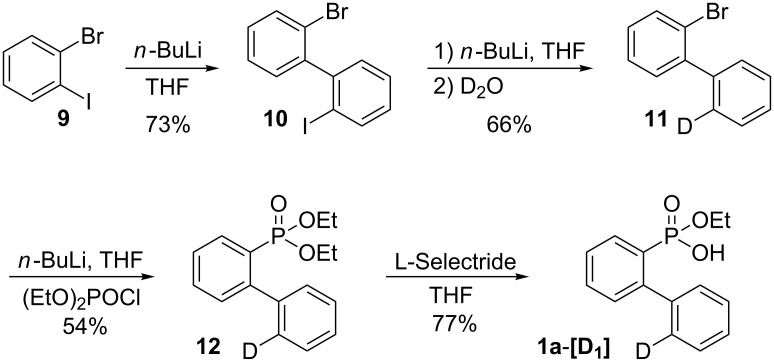
Preparation of **1a**-**[D_1_]**.

**Scheme 8:**
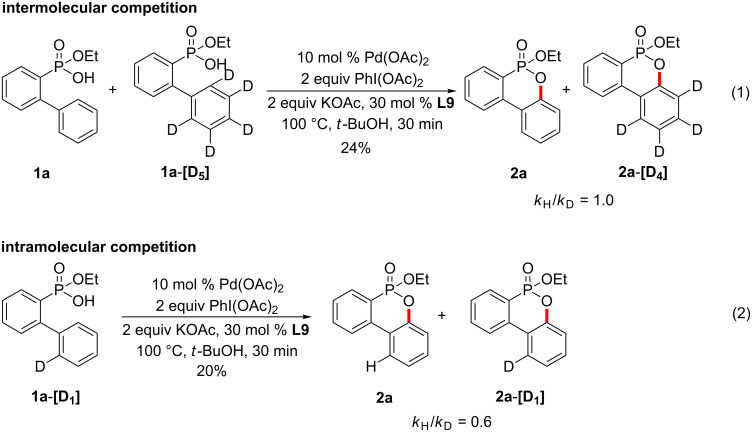
Studies with isotopically labelled compounds.

We carried out kinetic isotope effect (KIE) studies to prove the reaction mechanism (see Scheme 8). The required deuterium-labeled 2-(phenyl)phenylphosphonic acid monoethyl ester **1a**-**[D_5_]** was efficiently prepared by a Suzuki reaction of deuterated bromobenzene (**6**) with 2-bromophenylboronic acid (**5**), a lithium bromide exchange reaction of 2-bromo deuterated biphenyl **7** followed by diethylphosphinylation with diethyl chlorophosphate, and C–O cleavage of diethyl 2-(phenyl)phenylphosphonate by using L-Selectride (Scheme 6). In addition, the deuterium-labeled 2-(phenyl)phenylphosphonic acid monoethyl ester **1a**-**[D_1_]** was obtained by the lithium bromide exchange reaction of 2‘-bromo-2-iodo-1,1‘-biphenyl (**10**) and the treatment of D_2_O, diethylphosphinylation with diethyl chlorophosphate, and C–O cleavage of diethyl 2-(phenyl)phenylphosphonate by using L-Selectride (Scheme 7).

In the case of an intermolecular competition reaction using **1a** and **1a**-**[D_5_]**, a KIE was detected (*k*_H_/*k*_D_ = 1.0; Scheme 8, reaction 1) [51–52]. Also, an intramolecular competition reaction using **1a**-**[D_1_]** was carried out to give KIE (*k*_H_/*k*_D_ = 0.6; Scheme 8, reaction 2). These results indicate that the C–H cleavage at the *ortho*-position of 2-(phenyl)phenylphosphonic acid monoethyl ester is not involved in the rate-limiting step and the C–H bond metallation is reversible.

**Scheme 9:**
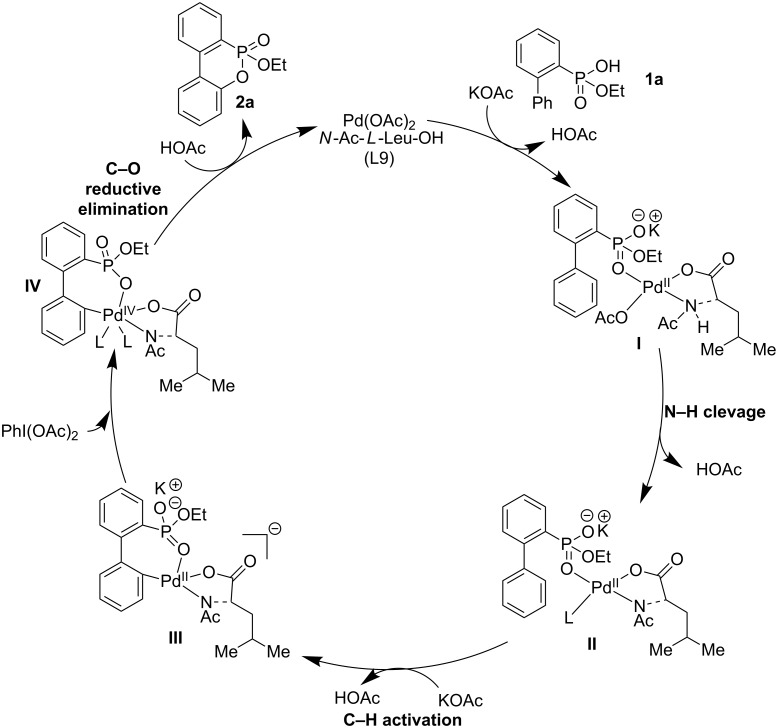
A plausible mechanism.

To elucidate the mechanism of the present reaction, the reaction was conducted with a stoichiometric amount of Pd(OAc)_2_ and without the oxidant PhI(OAc)_2_. However, no cyclized product was observed. This result indicates that the C–O reductive elimination from Pd(II) is not favorable. Because both the intermolecular and intramolecular competition experiments exhibited no significant kinetic isotope effect (*k*_H_/*k*_D_ = 1.0 and 0.6; Scheme 8), we hypothesize that the C–O reductive elimination step is the rate-determining step. A feasible mechanism involving the Pd(II)/Pd(IV) catalytic cycle is described in Scheme 9. The C–H activation might be efficiently accelerated by the N–H activation propelled by *N*-Ac-*L*-Leu-OH (L9) as a ligand [53–55], resulting in the formation of palladacycle **III**. Thereafter, ethoxy dibenzooxaphosphorin oxide **2a** is obtained from the oxidation of the Pd(II) to Pd(IV) species **IV** and the subsequent C–O reductive elimination.

## Conclusion

In this paper, we have developed an efficient synthetic method for a wide range of ethoxy dibenzooxaphosphorin oxides starting from 2-(aryl)arylphosphonic acid monoethyl esters and employing Pd-catalyzed C(sp^2^)–H activation/C–O formation under aerobic conditions. Oxidative cyclization by means of a Pd(II)/Pd(IV) catalytic cycle might play a role in the mechanism of the present reaction.

## Supporting Information

File 1 Experimental procedures, characterization data, and ^1^H and ^13^C NMR spectra of new compounds.
