# Supplementary material for: Synthesis of ethoxy dibenzooxaphosphorin oxides through palladium-catalyzed C(sp2)–H activation/C–O formation
Source: Beilstein J Org Chem. 2014 May 23;10:1220–7. doi: 10.3762/bjoc.10.120 (PMC4077535; doi:10.3762/bjoc.10.120)
Supplement: File 1 — Experimental procedures, characterization data, and 1H and 13C NMR spectra of new compounds. [file Beilstein_J_Org_Chem-10-1220-s001.pdf]

Supporting Information
for

Synthesis of ethoxy dibenzooxaphosphorin oxides through Palladium-catalyzed $C(sp^2)$–H activation/C–O formation

Seohyun Shin,[§] Dongjin Kang,[§] Woo Hyung Jeon and Phil Ho Lee*

Address: Center for Catalytic Organic Reactions, National Creative Research Laboratory (NCRL), Chuncheon 200-701, Republic of Korea and Department of Chemistry, Kangwon National University, Chuncheon 200-701, Republic of Korea

Email: Phil Ho Lee - phlee@kangwon.ac.kr

[*]Corresponding author

[§]These authors contributed equally to this work.

This paper is dedicated to Professor Bong Rae Cho (Korea University) on the occasion of his honorable retirement.

Experimental procedures, characterization data, and ^{1}H and ^{13}C NMR spectra of new compounds

Contents

1. Experimental section--- S3

1. 1. General-- S3

1.2. Additional data for reaction optimization --- S4

1.3. Preparation of 1,1'-biphenylphosphonic acid monoethyl esters (**1a**)----------------------- S7

1.4. Preparation of 2-(thiophene-2-yl)phenlyphosphonic acid monoethyl ester (**1n**)----- -----S8

1.5. Preparation of isotopically labeled compounds-- S8

1.6. Preparation of 6-ethoxy-6*H*-dibenz[*c*,*e*]oxaphosphorin-6-oxide (**2a**)--------------------- S9

1.7. Studies with isotopically labelled compounds--- S31

1.8. Intermolecular competition-- S31

1.9. Intramolecular competition-- S32

2. References-- S32

3. ^{1}H and ^{13}C NMR spectra -- S34

1. Experimental Section

1.1 General

Reactions were carried out in oven-dried glassware under air atmosphere. Commercial available reagents were used without purification. All reaction mixtures were stirred magnetically and were monitored by thin-layer chromatography using silica gel pre-coated glass plates, which were visualized with UV light and then, developed using either iodine or a solution of anisaldehyde. Flash column chromatography was carried out using silica gel (230-400 mesh). ^{1}H NMR (400 MHz) and ^{13}C NMR (100 MHz) spectra were recorded on a Brucker DPX FT spectrometer. Deuterated chloroform was used as the solvent and chemical shift values (δ) are reported in parts per million relative to the residual signals of this solvent (δ 7.24 for ^{1}H and δ 77.0 for ^{13}C). Infrared spectra were recorded on a JASCO FT/IR-460 plus FT-IR spectrometer as either a thin film or as a solid suspended in a potassium bromide pellet. Mass spectrometry was performed on a GC/HRMS spectrometer by electron impact (EI) ionization techniques from the KBSI. Melting points were determined in open capillary tube using Electrothermal 9100 apparatus.

1.2. Additional data for reaction optimization

Table 1: Effect of oxidant.[a]

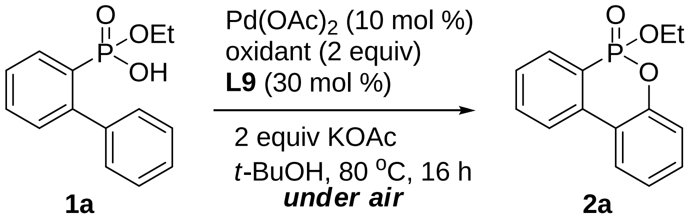

entry	oxidant	yield[b] [%]	entry	oxidant	yield[b] [%]
1	$K_2S_2O_8$	0	6	$CuCl_2$	0
2	BQ	0	7	CuBr	0
3	$[PhCO_2]_2$	0	8	AgOAc	0
4	$PhI(TFA)_2$	0	9	Ag_2CO_3	0
5	$Cu(OAc)_2$	0	10	Ag_2O	0

[a]Reaction condition : **1a** (0.2 mmol), $Pd(OAc)_2$ (10 mol %), *t*-BuOH (2.5 mL), 100 ^{o}C for 16 h.
[b]^{1}H NMR yield using CH_2Br_2 as an internal standard.

Table 2: Effect of base.[a]

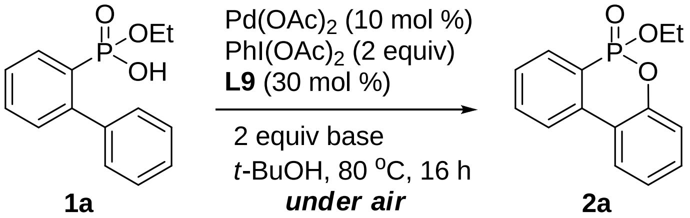

entry	base	yield[b] [%]	entry	base	yield[b] [%]
1	$KHCO_3$	31	10	CsOAc	51
2	K_2HPO_4	35	11	CsF	52
3	K_2CO_3	17	12	CsOPiv	46
4	KOH	33	13	LiOAc	9
5	KF	39	14	$LiPF_6$	12
6	KPF_6	0	15	Li_3PO_4	8
7	Na_2CO_3	26	16	LiF	8
8	NaOAc	42	17	LiI	5
9	NaF	9			

[a]Reaction condition : **1a** (0.2 mmol), $Pd(OAc)_2$ (10 mol %), *t*-BuOH (2.5 mL), 100 ^{o}C for 16 h.
[b]^{1}H NMR yield using CH_2Br_2 as an internal standard.

Table 3: Effect of solvent.[a]

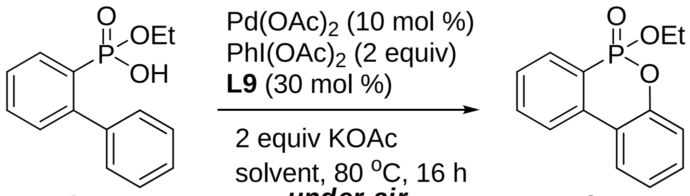

1a **2a**

entry	base	yield[b] [%]	entry	base	yield[b] [%]
1	DCE	7	6	HFIP	0
2	dioxane	0	7	THF	7
3	CH_3CN	4	8	toluene	6
4	*t*-AmOH	26	9	TFA	42
5	DMF	9	10	MeOH	14

[a]Reaction condition : **1a** (0.2 mmol), $Pd(OAc)_2$ (10 mol %), solvent (2.5 mL), 100 °C for 16 h.
[b]^{1}H NMR yield using CH_2Br_2 as an internal standard.

Table 4: Effect of temperature.[a]

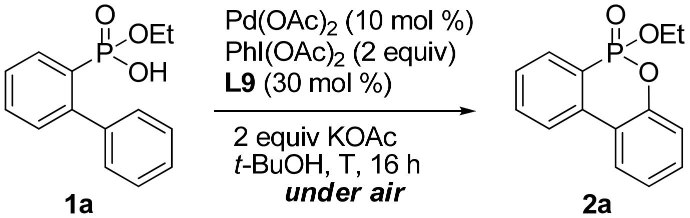

1a **2a**

entry	T [°C]	yield[b] [%]
1	60	20
2	100	61
3	120	50
4	140	8
5	160	7

[a]Reaction condition : **1a** (0.2 mmol), $Pd(OAc)_2$ (10 mol %), *t*-BuOH (2.5 mL), 16 h.
[b]^{1}H NMR yield using CH_2Br_2 as an internal standard.

Table 5: Effect of time.[a]

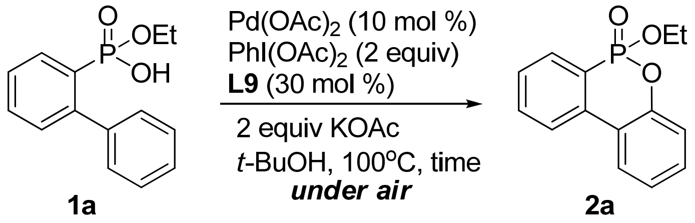

entry	t [h]	yield[b] [%]
1	1	32
2	2	40
3	4	45
4	8	51
5	12	61(55)

[a]Reaction condition : **1a** (0.2 mmol), $Pd(OAc)_2$ (10 mol %), *t*-BuOH (2.5 mL), 100 °C
[b]^{1}H NMR yield using CH_2Br_2 as an internal standard.

1.3. Preparation of 1,1'-biphenylphosphonic acid monoethyl esters (1a) [1-4]

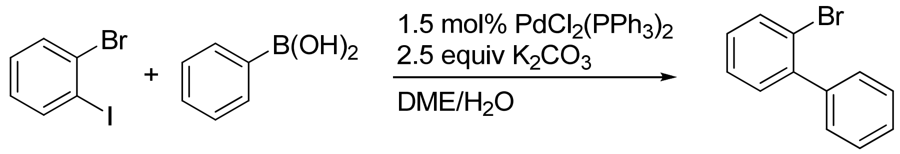

To a solution of $PdCl_2(PPh_3)_2$ (31.6 mg, 0.045 mmol) and potassium carbonate (1.04 g, 7.5 mmol) in water (1.2 mL) and DME (10.0 mL) were added 2-bromoiodobenzene (0.85 g, 3.0 mmol) and substrated phenylboronic acid (4.5 mmol). The reaction mixture was stirred at 80 ^oC for 5 h in oil bath until substrate disappeared on TLC. When the reaction was complete, water (10.0 mL) and ether (10.0 mL) were added. The aqueous layer was separated and extracted with ether (10.0 mL x 3). The combined organic layer was washed with brine and the organic fraction was dried with $MgSO_4$, filtered and concentrated under reduced pressure. The crude product was then purified by flash column chromatography (hexane) on silica gel, producing the title compound as a colorless oil (607.0 mg, 87%).

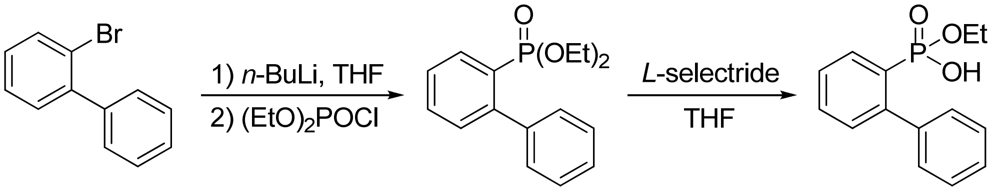

To a solution of 2-bromo-1,1'-biphenyl (2.60 mmol) in dry THF (8.7 mL) was added *n*-BuLi (2.5 M solution in *n*-hexane, 1.3 mL, 3.1 mmol) at 0 ^oC under nitrogen atmosphere. The mixture was stirred at 0 ^oC for 30 min and then, diethyl chlorophosphate (0.38 mL, 2.60 mmol) was added at 0 ^oC. The solution was stirred for 1 h at 0 ^oC, warmed to 25 ^oC and stirred for 3 h. When the reaction was complete, the solution was quenched with ammonium chloride. The aqueous layer was separated and extracted with ether (10 mL x 3) and the orgarnic fraction was dried with $MgSO_4$, filtered and concentrated under reduced pressure. The crude product was then purified by flash column chromatography (ethyl acetate:hexane = 1:1) on silica gel, producing the title compound as a colorless oil (580.0 mg, 77%).

A solution of 1,1'-biphenylphosphonic acid diethyl ester (580.0 mg, 2.0 mmol) in THF (6.7 mL) was treated with *L*-selectride (1.0 M solution in THF, 4.0 mL, 4.0 mmol) at 50 ^oC for 30 min. When the reaction was complete, the solution was quenched by addition of water.

The aqueous layer was extracted with ethyl acetate (20 mL x 3) to remove impurities, then acidified to pH 1 with 1 N HCl (10 mL) and extracted with ethyl acetate (20 mL x 5). The combined organic layer was dried with $MgSO_4$ and concentrated under reduced pressure. Then, the crude product was dried in vacuum to afford 1,1'-biphenylphosphonic acid monoethyl ester (420.0 mg, 80%) as a white crystalline solid.

1.4. Preparation of 2-(thiophene-2-yl)phenlyphosphonic acid monoethyl ester (1n)[5]

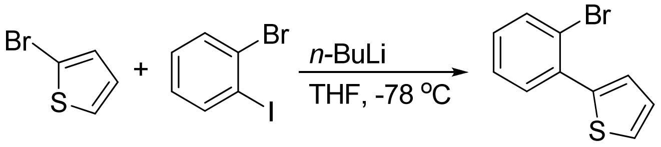

To a solution of 2-bromothiophene (9.0 mmol) in dry THF (30 mL) was added *n*-BuLi (2.5 M solution in *n*-hexane, 3.8 mL, 9.45 mmol) at -78 ^{o}C under nitrogen atmosphere. The reaction mixture was stirred at -78 ^{o}C for 30 min and then, 1-bromo-2-iodobenzene (9.0 mmol) was dropwise at -78 ^{o}C. The mixture was warmed to 25 ^{o}C and stirred for 1 h. When the reaction was complete, the solution was quenched with ammonium chloride. The aqueous layer was extracted with ethyl ether (3 x 20 mL) and the combined organic layer was dried over $MgSO_4$ and concentrated under reduced pressure.The crude product was then purified by flash column chromatography (hexane) on silica gel, producing the title compound as a yellow oil (1.70 g, 79%).

1.5. Preparation of isotopically labeled compounds.[6-7]

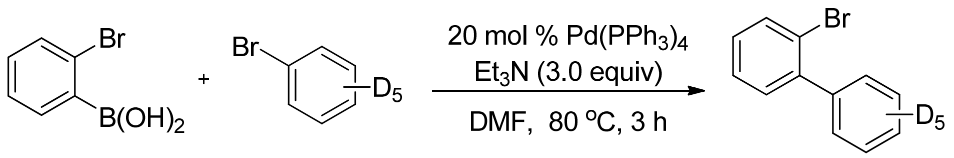

2-Bromophenylboronic acid (3.0 mmol) and bromobenzene-d_5 (3.0 mmol) in presence of tetrakis(triphenylphosphine)palladium (69.3 mg, 20 mol %) and triethylamine (911.0 mg, 6.0 mmol) in DMF (18 mL) were heated at 80 ^{o}C. After 3 h, it was allowed to cool to 25 ^{o}C and extracted with Et_2O (3 x 20 mL). Then, organic layer was washed with water and dried over $MgSO_4$ and concentrated under reduced pressure. The crude product was then purified by flash column chromatography (hexane) on silica gel, producing the title compound as a colorless oil (572.0 mg, 80%).

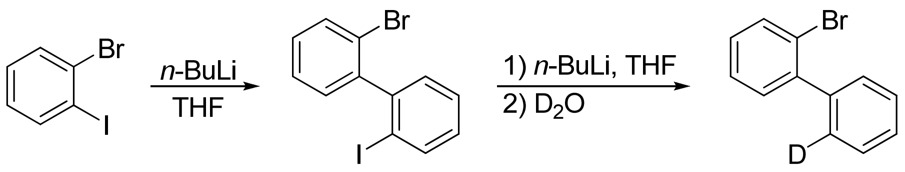

To a solution of 2-bromoiodobenzene (10 mmol) in THF (20 mL) was added *n*-BuLi (2.5 M solution in *n*-hexane, 2.0 mL, 5.0 mmol) at -78 ^{o}C under nitrogen atmosphere. The reaction mixture was then warmed to 25 ^{o}C over a two hours and hydrolyzed with a 1.0 M aqueous hydrochloric acid solution (20 mL). After the aqueous layer was extracted with diethyl ether (3 x 40 mL), the combined organic layer was dried over $MgSO_4$ and concentrated under reduced pressure. Upon crystallization from ethanol, 2-bromo-2'-iodobiphenyl was obtained as a colorless needle (2.58 g, 73%).

To a solution of 2-bromo-2'-iodobiphenyl (2.0 mmol) in THF (8.0 mL) was added *n*-BuLi (2.5 M solution in *n*-hexane, 0.8 mL, 2.0 mmol) at -78 ^{o}C under nitrogen atmosphere. The reaction mixture was stirred at -78 ^{o}C for 1 h and then, quenched by D_2O (3.0 mmol). After being stirred for 30 min, the mixture was poured into cold water and extracted with dichloromethane (3 x 15 mL). The combined organic layer was dried over $MgSO_4$ and concentrated under reduced pressure. The crude was purified by flash column chromatography (hexane) on silica gel, producing 2-bromo-2'-deuterio-1,1'-biphenyl as a colorless oil (0.31 g, 66%).

1.6. Preparation of 6-ethoxy-6*H*-dibenz[*c*,*e*]oxaphosphorin-6-oxide 2a.

1,1'-Biphenylphosphonic acid monoethyl ester **1a** (52.4 mg, 0.2 mmol), $Pd(OAc)_2$ (4.49 mg, 0.02 mmol), *N*-acetyl-L-leucine (10.4 mg, 0.06 mmol), $PhI(OAc)_2$ (128.8 mg, 0.4 mmol), KOAc (39.3 mg, 0.4 mmol) and *t*-BuOH (2.5 mL) were added to a 2.5 mL screw-top v-vial. The reaction mixture was stirred at 100 ^{o}C for 12 h under air atmosphere. After celite filtration and evaporation of the solvents in vacuo, the crude product was purified by column chromatography on silica gel (EtOAc/hexane = 1/1) to yield **2a** (28.9 mg, 55%) as a yellow oil.

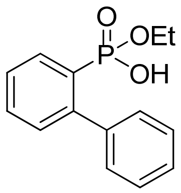

1a

1,1'-Biphenyl phosphonic acid monoethyl ester (1a) : White solid, m.p. 107-125 ^{o}C; ^{1}H NMR (400 MHz, $CDCl_3$) δ 12.21 (br, 1H), 8.05 (ddd, J = 14.8, 7.7, 1.2 Hz, 1H), 7.58-7.53 (m, 1H), 7.46-7.30 (m, 7H), 3.70 (m, 2H), 0.95 (t, J = 7.1 Hz, 3H); ^{13}C NMR (100 MHz, $CDCl_3$) δ 146.1 (d, J = 9.9 Hz), 141.4 (d, J = 4.3 Hz), 133.5 (d, J = 10.2 Hz), 132.0 (d, J = 2.9 Hz), 131.3 (d, J = 14.3 Hz), 129.4, 127.5, 127.4, 127.3 (d, J = 192.7 Hz), 126.8 (d, J = 14.8 Hz), 61.5 (d, J = 6.2 Hz), 15.7 (d, J = 7.3 Hz); ^{31}P NMR (161 MHz, $CDCl_3$) δ 20.81; IR (film): 3056, 2981, 2801, 2359, 1469, 1199, 1040, 988, 754, 701 cm^{-1}; HRMS (EI): *m/z* calcd for $C_{14}H_{15}O_3P$: 262.0759; found : 262.0760.

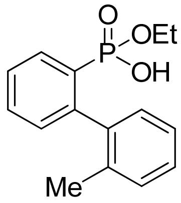

1b

2'-Methyl-1,1'-biphenyl phosphonic acid monoethyl ester (1b) : White solid, m.p. 83-87 ^{o}C; ^{1}H NMR (400 MHz, $CDCl_3$) δ 11.11 (br, 1H), 8.03-8.00 (m, 1H), 7.55-7.50 (m, 1H), 7.41-7.36 (m, 1H), 7.23-7.12 (m, 5H), 3.74-3.57 (m, 2H), 2.02 (s, 3H), 1.01 (t, J = 7.1 Hz, 3H); ^{13}C NMR (100 MHz, $CDCl_3$) δ 145.4 (d, J = 10.2 Hz), 140.4 (d, J = 4.1 Hz), 136.3, 133.2 (d, J = 10.4 Hz), 131.8 (d, J = 2.9 Hz), 130.8 (d, J = 14.5 Hz), 130.0, 129.3, 127.8 (d, J = 194.5 Hz), 127.6, 126.7 (d, J = 15.2 Hz), 124.5, 61.3 (d, J = 6.6 Hz), 20.3, 15.9 (d, J = 6.9 Hz); ^{31}P NMR (161 MHz, $CDCl_3$) δ 20.35; IR (film): 3057, 2980, 2309, 1468, 1198, 1040, 988, 760 cm^{-1}; HRMS (EI): *m/z* calcd for $C_{15}H_{17}O_3P$: 276.0915; found : 276.0916.

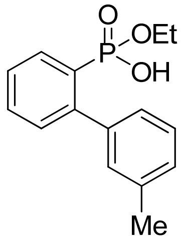

1c

3'-Methyl-1,1'-biphenyl phosphonic acid monoethyl ester (1c) : White solid, m.p. 80-87 °C; ^{1}H NMR (400 MHz, CDCl$_3$) δ 11.08 (br, 1H), 8.03 (ddd, J = 14.8, 7.7, 1.0 Hz, 1H), 7.51 (tt, J = 11.3, 1.5 1H), 7.41-7.37 (m, 1H), 7.32-7.29 (m, 1H), 7.24-7.23 (m, 3H), 3.72 (p, J = 7.2 Hz, 2H), 2.37 (s, 3H), 0.98 (t, J = 7.1 Hz, 3H); ^{13}C NMR (100 MHz, CDCl$_3$) δ 146.2 (d, J = 10.1 Hz), 141.3 (d, J = 4.3 Hz), 136.9, 133.5 (d, J = 10.1 Hz), 131.9 (d, J = 2.8 Hz), 131.2 (d, J = 14.4 Hz), 130.1, 128.1, 127.4, 127.3 (d, J = 192.4 Hz), 126.7 (d, J = 15.1 Hz), 126.3, 61.4 (d, J = 6.3 Hz), 21.4, 15.7 (d, J = 7.4 Hz); ^{31}P NMR (161 MHz, CDCl$_3$) δ 20.84; IR (film): 2980, 2904, 2297, 1467, 1200, 1143, 1042, 989, 790 cm^{-1}; HRMS (EI): *m/z* calcd for $C_{15}H_{17}O_3P$: 276.0915; found : 276.0916.

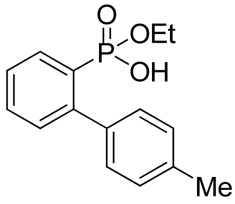

1d

4'-Methyl-1,1'-biphenyl phosphonic acid monoethyl ester (1d) : White solid, m.p. 80-85 °C; ^{1}H NMR (400 MHz, CDCl$_3$) δ 12.08 (br, 1H), 8.03 (dd, J = 14.7, 7.1 Hz, 1H), 7.52 (t, J = 7.5 1H), 7.40-7.28 (m, 4H), 7.17 (s, 1H), 7.15 (s, 1H), 3.70 (m, 2H), 2.36 (s, 3H), 0.97 (t, J = 7.0 Hz, 3H); ^{13}C NMR (100 MHz, CDCl$_3$) δ 146.2 (d, J = 10.1 Hz), 138.5 (d, J = 4.2 Hz), 137.0 133.6 (d, J = 10.1 Hz), 131.9 (d, J = 2.2 Hz), 131.4 (d, J = 14.3 Hz), 129.3, 128.2, 126.6 (d, J = 14.8 Hz), 126.3, 61.5 (d, J = 6.2 Hz), 21.3, 15.8 (d, J = 7.4 Hz); ^{31}P NMR (161 MHz, CDCl$_3$) δ 20.81; IR (film): 2981, 2925, 2331, 1469, 1200, 1041, 989, 821, 765 cm^{-1}; HRMS (EI): *m/z* calcd for $C_{15}H_{17}O_3P$: 276.0915; found : 276.0916.

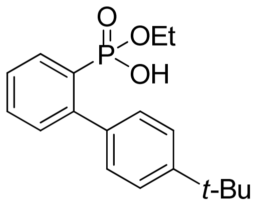

1e

4'-*tert*-Butyl- 1,1'-biphenyl phosphonic acid monoethyl ester (1e) : Ivory solid, m.p. 100-123 °C; ^{1}H NMR (400 MHz, $CDCl_3$) δ 11.28 (br, 1H), 8.06 (ddd, J = 15.0, 7.7, 1.0 Hz, 1H), 7.55 (tt, J = 11.3, 1.4 Hz, 1H), 7.44-7.33 (m, 6H), 3.72 (p, J = 7.1 Hz, 2H), 1.34 (s, 9H), 0.89 (t, J = 7.0 Hz, 3H); ^{13}C NMR (100 MHz, $CDCl_3$) δ 150.3, 146.1 (d, J = 10.1 Hz), 138.4 (d, J = 4.2 Hz), 133.6 (d, J = 10.4 Hz), 132.1 (d, J = 2.9 Hz), 131.4 (d, J = 14.3 Hz), 129.0, 126.8 (d, J = 193.6 Hz), 126.7 (d, J = 15.0 Hz), 124.5, 61.6 (d, J = 6.5 Hz), 34.6, 31.4, 15.5 (d, J = 7.5 Hz); ^{31}P NMR (161 MHz, $CDCl_3$) δ 21.35; IR (film): 3056, 2963, 2308, 1591, 1471, 1195, 1041, 989, 835, 756 cm^{-1}; HRMS (EI): *m/z* calcd for $C_{18}H_{23}O_3P$: 318.1385; found : 318.1388.

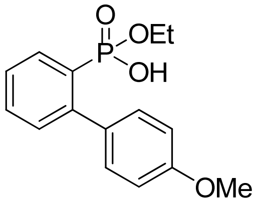

1f

4'-Methoxy- 1,1'-biphenyl phosphonic acid monoethyl ester (1f) : White solid, m.p. 120-123 °C; ^{1}H NMR (400 MHz, $CDCl_3$) δ 12.67 (br, 1H), 8.03 (ddd, J = 14.8, 7.7, 0.9 Hz, 1H), 7.53 (t, J = 7.5 Hz, 1H), 7.41-7.37 (m, 3H), 7.30 (t, J = 6.6 Hz, 1H), 6.90 (d, J = 8.7 Hz, 2H), 3.82 (s, 3H), 3.73 (p, J = 5.8 Hz, 2H), 0.99 (t, J = 7.1 Hz, 3H); ^{13}C NMR (100 MHz, $CDCl_3$) δ 159.0, 145.8 (d, J = 14.3 Hz), 130.6, 127.2 (d, J = 192.2 Hz), 126.5 (d, J = 15.0 Hz), 113.0, 61.5 (d, J = 6.2 Hz), 55.2, 15.8 (d, J = 7.3 Hz); ^{31}P NMR (161 MHz, $CDCl_3$) δ 21.21; IR (film): 3057, 2904, 2295, 1517, 1244, 1179, 1038, 988, 768 cm^{-1}; HRMS (EI): *m/z* calcd for $C_{15}H_{17}O_4P$: 292.0864; found : 292.0981.

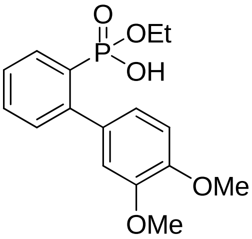

1g

3',4'-Dimethoxy- 1,1'-biphenyl phosphonic acid monoethyl ester (1g) : Yellow solid, m.p. 128-135 °C; ^{1}H NMR (400 MHz, $CDCl_3$) δ 11.23 (br, 1H), 8.07 (dd, J = 14.8, 7.7 Hz, 1H), 7.64-7.50 (m, 7H), 7.46-7.33 (m, 5H), 3.77 (m, 2H), 0.94 (t, J = 7.0 Hz, 3H); ^{13}C NMR (100 MHz, $CDCl_3$) δ 148.4, 147.9, 145.9 (d, J = 10.1 Hz), 134.1 (d, J = 4.3 Hz), 133.7 (d, J = 10.1 Hz), 132.0 (d, J = 2.8 Hz), 131.4 (d, J = 14.2 Hz), 127.2 (d, J = 192.0 Hz), 126.6 (d, J = 15.0 Hz), 121.5, 113.3, 110.3, 61.6 (d, J = 6.2 Hz), 55.9 (d, J = 3.5 Hz), 15.8 (d, J = 7.3 Hz); ^{31}P NMR (161 MHz, $CDCl_3$) δ 21.25; IR (film): 3057, 2835, 2362, 2254, 1605, 1520, 1470, 1247, 1141, 1029, 732 cm^{-1}; HRMS (EI): *m/z* calcd for $C_{16}H_{19}O_5P$: 322.0970; found : 322.0966.

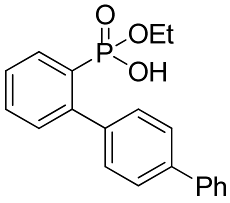

1h

4'-Phenyl-1,1'-biphenyl phosphonic acid monoethyl ester (1h) : White solid, m.p. 166-175 °C; ^{1}H NMR (400 MHz, $CDCl_3$) δ 11.23 (br, 1H), 8.07 (dd, J = 14.8, 7.7 Hz, 1H), 7.64-7.50 (m, 7H), 7.46-7.33 (m, 5H), 3.77 (m, 2H), 0.94 (t, J = 7.0 Hz, 3H); ^{13}C NMR (100 MHz, DMSO-d_6) δ 144.5 (d, J = 9.4 Hz), 140.7 (d, J = 3.9 Hz), 139.8, 138.8, 133.0 (d, J = 9.2 Hz), 131.5, 131.2 (d, J = 13.2 Hz), 129.8, 129.4 (d, J = 181.6 Hz), 128.5, 127.4, 126.9 (d, J = 13.9 Hz), 126.6, 125.6, 60.2 (d, J = 5.6 Hz), 15.8 (d, J = 7.0 Hz) ; ^{31}P NMR (161 MHz, $CDCl_3$) δ 21.02; IR (film): 3056, 2981, 2348, 1591, 1470, 1195, 1038, 988, 841, 751 cm^{-1}; HRMS (EI): *m/z* calcd for $C_{20}H_{19}O_3P$: 338.1072; found : 338.1070.

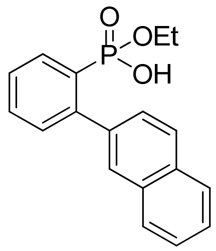

1i

2-(Naphthalen-2-yl)phenlyphosphonic acid monoethyl ester (1i) : White solid, m.p. 109-125 °C; ^{1}H NMR (400 MHz, $CDCl_3$) δ 10.60 (br, 1H), 8.05 (ddd, *J* = 14.8, 7.7, 0.92 Hz, 1H), 7.87 (s, 1H), 7.83-7.80 (m, 2H), 7.60-7.54 (m, 2H), 7.48-7.37 (m, 4H), 3.61 (p, *J* = 7.3 Hz, 2H), 0.81 (t, *J* = 7.1 Hz, 3H); ^{13}C NMR (100 MHz, $CDCl_3$) δ 146.0 (d, *J* = 9.9 Hz), 138.8 (d, *J* = 4.3 Hz), 133.5 (d, *J* = 10.1 Hz), 132.8, 132.6, 131.9 (d, *J* = 2.9 Hz), 131.5 (d, *J* = 14.4 Hz), 128.0 (d, *J* = 53.2 Hz), 127.9 (d, *J* = 64.9 Hz), 127.5 (d, *J* = 192.9 Hz), 126.9, 126.9 (d, *J* = 14.7 Hz), 126.0, 61.4 (d, *J* = 6.4 Hz), 15.7 (d, *J* = 7.2 Hz); ^{31}P NMR (161 MHz, $CDCl_3$) δ 20.85; IR (film): 3055, 2981, 2364, 1481, 1394, 1196, 1039, 1030, 879, 770 cm^{-1}; HRMS (EI): *m/z* calcd for $C_{18}H_{17}O_3P$: 312.0915; found : 312.0917.

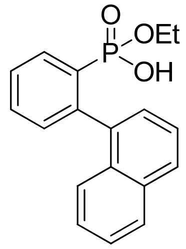

1j

2-(Naphthalen-1-yl)phenlyphosphonic acid monoethyl ester (1j) : White solid, m.p. 146-152 °C; ^{1}H NMR (400 MHz, $CDCl_3$) δ 12.04 (br, 1H), 8.04 (ddd, *J* = 14.8, 7.7, 1.0 Hz, 1H), 7.87 (d, *J* = 1.1 Hz, 1H), 7.84-7.79 (m, 3H), 7.60-7.54 (m, 2H), 7.48-7.37 (m, 4H), 3.60 (p, *J* = 7.2 Hz, 2H), 0.80 (t, *J* = 7.1 Hz, 3H); ^{13}C NMR (100 MHz, $CDCl_3$) δ 144.0 (d, *J* = 9.6 Hz), 138.1 (d, *J* = 3.6 Hz), 133.5 (d, *J* = 10.5 Hz), 133.4, 132.5, 132.0 (d, *J* = 14.2 Hz), 131.5 (d, *J* = 14.4 Hz), 128.0 (d, *J* = 53.2 Hz), 127.9 (d, *J* = 64.9 Hz), 127.5 (d, *J* = 192.9 Hz), 129.9, 127.9, 127.8, 127.1, 126.5, 125.5 (d, *J* = 20.5 Hz), 124.7, 61.2 (d, *J* = 6.6 Hz), 15.2 (d, *J* = 7.2 Hz); ^{31}P NMR (161 MHz, $CDCl_3$) δ 20.80; IR (film): 3056, 2981, 2368, 2264, 1655, 1393,

1198, 1041, 990, 779 cm^{-1}; HRMS (EI): *m/z* calcd for $C_{18}H_{17}O_3P$: 312.0915; found : 312.0912.

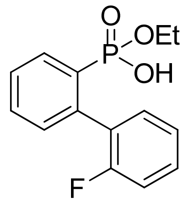

1k

2'-Fluoro- 1,1'-biphenyl phosphonic acid monoethyl ester (1k) : Yellow solid, m.p. 72-91 ^{o}C; ^{1}H NMR (400 MHz, $CDCl_3$) δ 11.40 (br, 1H), 8.01 (ddd, *J* = 14.6, 7.7, 1.1 Hz, 1H), 7.58-7.53 (m, 1H), 7.46-7.41 (m, 1H), 7.37-7.28 (m, 3H), 3.74 (p, *J* = 7.2 Hz, 2H), 1.03 (t, *J* = 7.1 Hz, 3H); ^{13}C NMR (100 MHz, $CDCl_3$) δ 159.7 (d, *J* = 245.9 Hz), 139.2 (d, *J* = 9.3 Hz), 133.5 (d, *J* = 9.8 Hz), 132.2 (d, *J* = 2.3 Hz), 131.8 (d, *J* = 2.8 Hz), 131.5 (d, *J* = 14.1 Hz), 129.5 (d, *J* = 8.0 Hz), 128.3 (d, *J* = 192.8 Hz), 127.5 (d, *J* = 15.0 Hz), 123.0 (d, *J* = 3.5 Hz), 115.0 (d, 22.3 Hz), 61.5 (d, *J* = 6.1 Hz), 15.8 (d, *J* = 7.3 Hz); ^{31}P NMR (161 MHz, $CDCl_3$) δ 19.93; IR (film): 3060, 2982, 2328, 1469, 1210, 1094, 1037, 989, 757, 684 cm^{-1}; HRMS (EI): *m/z* calcd for $C_{14}H_{14}FO_3P$: 280.0665; found : 280.0666.

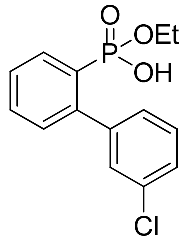

1l

3'-Chloro- 1,1'-biphenyl phosphonic acid monoethyl ester (1l) : White solid, m.p. 75-83 ^{o}C; ^{1}H NMR (400 MHz, $CDCl_3$) δ 12.67 (br, 1H), 8.03 (dd, *J* = 14.7, 7.6 Hz, 1H), 7.56 (t, *J* = 7.5 Hz, 1H), 7.46-7.41 (m, 2H), 7.34-7.28 (m, 4H), 3.78 (p, *J* = 7.2 Hz, 2H), 1.03 (t, *J* = 7.1 Hz, 3H); ^{13}C NMR (100 MHz, $CDCl_3$) δ 144.4 (d, *J* = 9.7 Hz), 143.0 (d, *J* = 4.3 Hz), 133.5 (d, *J* = 9.9 Hz), 133.3, 132.1 (d, *J* = 2.9 Hz), 131.0 (d, *J* = 14.2 Hz), 128.7, 127.8, 127.5, 127.3 (d, *J* = 193.1 Hz), 127.9 (d, *J* = 14.9 Hz), 61.5 (d, *J* = 6.5 Hz), 15.8 (d, *J* = 7.3 Hz); ^{31}P NMR (161 MHz, $CDCl_3$) δ 20.43; IR (film): 3061, 2982, 2359, 1462, 1200, 1039, 990, 909, 734,

697 cm^{-1}; HRMS (EI): *m/z* calcd for $C_{14}H_{14}ClO_3P$: 296.0369; found : 296.0367.

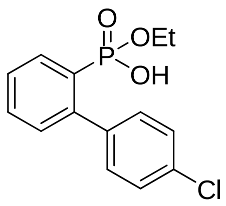

1m

4'-Chloro- 1,1'-biphenyl phosphonic acid monoethyl ester (1m) : White solid, m.p. 130-132 °C; ^{1}H NMR (400 MHz, CDCl$_3$) δ 12.58 (br, 1H), 8.02 (ddd, *J* = 14.8, 7.7, 1.0 Hz, 1H), 7.56 (t, *J* = 7.6 Hz, 1H), 7.43 (td, *J* = 11.4, 3.5 Hz, 1H), 7.38-7.36 (m, 2H), 7.34-7.26 (m, 3H), 3.75 (p, *J* = 7.2 Hz, 2H), 1.01 (t, *J* = 7.1 Hz, 3H); ^{13}C NMR (100 MHz, CDCl$_3$) δ 144.4 (d, *J* = 10.0 Hz), 139.7 (d, *J* = 4.3 Hz), 133.5 (d, *J* = 2.5 Hz), 133.4, 132.1 (d, *J* = 2.8 Hz), 131.2 (d, *J* = 14.1 Hz), 130.8, 127.7, 127.2 (d, *J* = 192.6 Hz), 127.2 (d, *J* = 14.9 Hz), 61.6 (d, *J* = 6.3 Hz), 15.8 (d, *J* = 7.2 Hz); ^{31}P NMR (161 MHz, CDCl$_3$) δ 20.38; IR (film): 2982, 2285, 1469, 1200, 1039, 990, 831, 764 cm^{-1}; HRMS (EI): *m/z* calcd for $C_{14}H_{14}ClO_3P$: 296.0369; found : 296.0370.

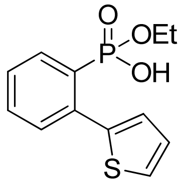

1n

2-(Thiophene-2-yl)phenlyphosphonic acid monoethyl ester (1n) : Brown solid, m.p. 91-101 °C; ^{1}H NMR (400 MHz, CDCl$_3$) δ 12.64 (br, 1H), 8.08 (ddd, *J* = 14.8, 7.8, 1.2 Hz, 1H), 7.53 (tt, *J* = 11.3, 1.5 Hz, 1H), 7.50-7.46 (m, 1H), 7.44-7.39 (m, 2H), 7.32 (dd, *J* = 5.1, 1.2 Hz, 1H), 3.83 (m, 2H), 1.06 (t, *J* = 7.1 Hz, 3H); ^{13}C NMR (100 MHz, CDCl$_3$) δ 141.4 (d, *J* = 5.0 Hz), 138.2 (d, *J* = 8.9 Hz), 134.1 (d, *J* = 9.8 Hz), 132.3 (d, *J* = 13.5 Hz), 132.0 (d, *J* = 2.9 Hz), 128.7, 128.0 (d, *J* = 192.6 Hz), 127.4 (d, *J* = 14.7 Hz), 127.0, 125.8, 61.8 (d, *J* = 6.3 Hz), 15.9 (d, *J* = 7.5 Hz); ^{31}P NMR (161 MHz, CDCl$_3$) δ 20.44; IR (film): 3059, 2982, 2903, 2288, 1590, 1471, 1199, 1143, 1039, 849, 766, 701 cm^{-1}; HRMS (EI): *m/z* calcd for $C_{12}H_{13}O_3PS$: 268.0323; found : 268.0321.

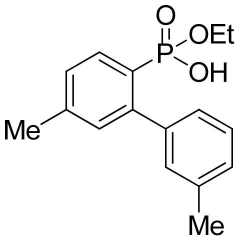

3a

3'-Methyl-5-methyl-1,1'-biphenyl phosphonic acid monoethyl ester (3a) : White solid, m.p. 77-81 °C; ^{1}H NMR (400 MHz, $CDCl_3$) δ 12.07 (br, 1H), 7.91 (dd, *J* = 14.6, 7.9 Hz, 1H), 7.24-7.18 (m, 4H), 7.14-7.12 (m, 2H), 3.69 (p, *J* = 7.2 Hz, 2H), 2.40 (s, 3H), 2.36 (s, 3H), 0.95 (t, *J* = 7.1 Hz, 3H); ^{13}C NMR (100 MHz, $CDCl_3$) δ 146.2 (d, *J* = 10.6 Hz), 142.3 (d, *J* = 3.1 Hz), 141.4 (d, *J* = 4.0 Hz), 136.9, 133.7 (d, *J* = 10.7 Hz), 132.1 (d, *J* = 14.7 Hz), 130.1, 128.0, 127.4, 127.3 (d, *J* = 15.3 Hz), 126.5, 124.1 (d, *J* = 194.9 Hz), 61.3 (d, *J* = 6.3 Hz), 21.5, 21.4, 15.7 (d, *J* = 7.4 Hz); ^{31}P NMR (161 MHz, $CDCl_3$) δ 21.48; IR (film): 3030, 2980, 2297, 1603, 1392, 1196, 1041, 987, 789, 664 cm^{-1}; HRMS (EI): *m/z* calcd for $C_{16}H_{19}O_3P$: 290.1072; found : 290.1071.

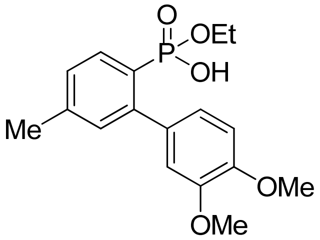

3b

3',4'-Dimethoxy-5-mehtyl-1,1'-biphenyl phosphonic acid monoethyl ester (3b) : White solid, m.p. 40-50 °C; ^{1}H NMR (400 MHz, $CDCl_3$) δ 12.87 (br, 1H), 7.92 (dd, *J* = 7.9, 14.6 Hz, 1H), 7.22-7.20 (m, 1H), 7.16 (d, *J* = 5.2, 1H), 7.09 (d, *J* = 1.5, 1H), 7.00-6.97 (m, 1H), 7.00-6.97 (m, 1H), 6.90-6.82 (m, 1H), 3.894 (s, 3H), 3.889 (s, 3H), 3.71 (p, *J* = 7.2 Hz, 2H), 2.41 (s, 3H), 0.97 (t, *J* = 7.1 Hz, 3H); ^{13}C NMR (100 MHz, $CDCl_3$) δ 148.1 (d, *J* = 47.0 Hz), 145.8 (d, *J* = 10.6 Hz), 142.5 (d, *J* = 3.2 Hz), 134.2 (d, *J* = 5.1 Hz), 133.8 (d, *J* = 10.4 Hz), 132.2 (d, *J* = 14.6 Hz), 127.3 (d, *J* = 15.4 Hz), 125.0, 123.1, 121.5, 113.2, 110.3, 61.4 (d, *J* = 6.2 Hz), 55.9, 55.8, 21.5, 15.8 (d, *J* = 7.3 Hz); ^{31}P NMR (161 MHz, $CDCl_3$) δ 21.87; IR (film): 2936, 2254, 1603, 1517, 1248, 1171, 1029, 732 cm^{-1}; HRMS (EI): *m/z* calcd for $C_{17}H_{21}O_5P$:

336.1127; found : 336.1123.

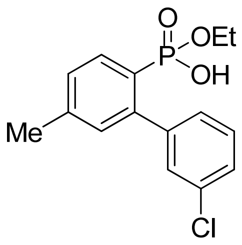

3c

3'-Chloro-5-methyl-1,1'-biphenyl phosphonic acid monoethyl ester (3c) : Yellow solid, m.p. 107-110 ^{o}C; ^{1}H NMR (400 MHz, $CDCl_3$) δ 12.66 (br, 1H), 7.90 (dd, J = 14.5, 7.9 Hz, 1H), 7.34-7.22 (m, 4H), 7.10 (d, J = 4.8 Hz, 1H), 3.75 (p, J = 7.2 Hz, 2H), 2.40 (s, 3H), 1.01 (t, J = 7.0 Hz, 3H); ^{13}C NMR (100 MHz, $CDCl_3$) δ 144.4 (d, J = 10.2 Hz), 143.1 (d, J = 4.0 Hz), 142.6 (d, J = 2.9 Hz), 133.7 (d, J = 10.3 Hz), 133.2, 131.9 (d, J = 14.5 Hz), 129.4, 128.7, 127.9 (d, J = 15.3 Hz), 127.8, 127.4, 124.2 (d, J = 195.5 Hz), 61.4 (d, J = 6.4 Hz), 21.5, 15.8 (d, J = 7.3 Hz); ^{31}P NMR (161 MHz, $CDCl_3$) δ 20.87; IR (film): 3065, 2982, 2355, 1619, 1436, 1270, 1034, 771, 618 cm^{-1}; HRMS (EI): *m/z* calcd for $C_{15}H_{16}ClO_3P$: 310.0526; found : 310.0529.

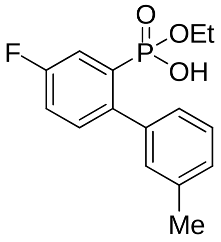

3d

3'-Methyl-1,1'-biphenyl-4-fluoro phosphonic acid monoethyl ester (3d) : White solid, m.p. 93-95 ^{o}C; ^{1}H NMR (400 MHz, $CDCl_3$) δ 10.65 (br, 1H), 7.70 (ddd, J = 9.0, 15.9, 2.7 Hz, 1H), 7.31-7.20 (m, 5H), 7.15 (d, J = 7.0 Hz, 1H), 3.74 (p, J = 7.2 Hz, 2H), 2.37 (s, 3H), 1.00 (t, J = 7.1 Hz, 3H); ^{13}C NMR (100 MHz, $CDCl_3$) δ 161.2 (dd J = 21.2, 248.3 Hz), 142.3 (dd, J = 3.6, 9.5 Hz), 140.2 (d, J = 3.9 Hz), 137.1, 133.2 (dd, J = 7.0, 17.1 Hz), 130.2, 129.4 (dd, J = 193.5, 23.2 Hz), 128.3, 127.5, 126.6, 120.2 (dd, J = 10.7, 23.2 Hz), 118.8 (dd, J = 2.8, 20.8 Hz), 61.8 (d, J = 6.4 Hz), 21.4, 15.7 (d, J = 7.3 Hz); ^{31}P NMR (161 MHz, $CDCl_3$) δ 18.80 (d, J =

7.7 Hz); IR (film): 2981, 2349, 1469, 1389, 1213, 1038, 993, 788, 707 cm^{-1}; HRMS (EI): *m/z* calcd for $C_{15}H_{16}FO_3P$: 294.0821; found : 294.0823.

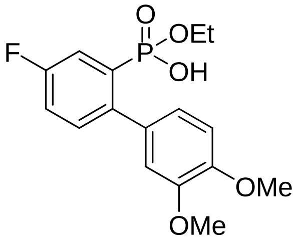

3e

3',4'-Dimethoxy-4-fluoro-1,1'-biphenyl phosphonic acid monoethyl ester (3e) : Yellow solid, m.p. 163-167 °C; ^{1}H NMR (400 MHz, $CDCl_3$) δ 10.88 (br, 1H), 7.72 (ddd, J = 15.9, 9.1, 2.8 Hz, 1H), 7.35-7.29 (m, 1H), 7.26-7.21 (m, 1H), 7.05 (d, J = 2.0, 1H), 6.96-6.94 (m, 1H), 6.88-6.86 (m, 1H), 3.90 (s, 3H), 3.88 (s, 3H), 3.74 (p, J = 7.2 Hz, 2H), 1.00 (t, J = 7.0 Hz, 3H); ^{13}C NMR (100 MHz, $CDCl_3$) δ 161.2 (dd, J = 21.3, 248.5 Hz), 148.2 (d, J = 60.7 Hz), 141.9 (dd, J = 9.5, 3.6 Hz), 133.4 (dd, J = 16.7, 7.2 Hz), 132.9 (d, J = 4.2 Hz), 130.3, 128.4, 121.7, 120.3 (dd, J = 23.3, 10.6 Hz), 118.9 (dd, J = 20.6, 3.0 Hz), 113.3, 110.3, 61.9 (d, J = 6.3 Hz), 55.9, 55.8, 15.8 (d, J = 7.2 Hz); ^{31}P NMR (161 MHz, $CDCl_3$) δ 18.83; IR (film): 2938, 1602, 1482, 1246, 1140, 1026, 765 cm^{-1}; HRMS (EI): *m/z* calcd for $C_{16}H_{18}FO_5P$: 340.0876; found : 340.0878.

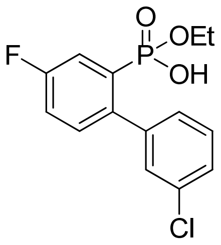

3f

3'-Chloro-4-fluoro-1,1'-biphenyl phosphonic acid monoethyl ester (3f) : Yellow solid, m.p. 163-167 °C; ^{1}H NMR (400 MHz, $CDCl_3$) δ 10.88 (br, 1H), 7.72 (ddd, J = 15.9, 9.1, 2.8 Hz, 1H), 7.35-7.29 (m, 1H), 7.26-7.21 (m, 1H), 7.05 (d, J = 2.0, 1H), 6.96-6.94 (m, 1H), 6.88-6.86 (m, 1H), 3.90 (s, 3H), 3.88 (s, 3H), 3.74 (p, J = 7.2 Hz, 2H), 1.00 (t, J = 7.0 Hz, 3H); ^{13}C NMR (100 MHz, $CDCl_3$) δ 161.2 (dd, J = 21.3, 248.5 Hz), 148.2 (d, J = 60.7 Hz),

141.9 (dd, J = 9.5, 3.6 Hz), 133.4 (dd, J = 16.7, 7.2 Hz), 132.9 (d, J = 4.2 Hz), 130.3, 128.4, 121.7, 120.3 (dd, J = 23.3, 10.6 Hz), 118.9 (dd, J = 20.6, 3.0 Hz), 113.3, 110.3, 61.9 (d, J = 6.3 Hz), 55.9, 55.8, 15.8 (d, J = 7.2 Hz); ^{31}P NMR (161 MHz, $CDCl_3$) δ 18.83; IR (film): 2984, 2287, 1600, 1462, 1212, 995, 786, 701 cm^{-1}; HRMS (EI): *m/z* calcd for $C_{14}H_{13}ClFO_3P$: 314.0275; found : 314.0272.

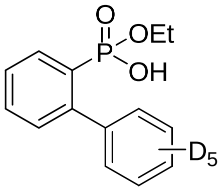

1a-[D$_5$]

2',3',4',5',6'-pentadeuterio-1,1'-biphenyl phosphonic acid monoethyl ester (1a-D$_5$) : White solid, m.p. 114-118 °C; ^{1}H NMR (400 MHz, $CDCl_3$) δ 11.00 (br, 1H), 8.04 (ddd, J = 14.8, 7.6, 1.2 Hz, 1H), 7.55 (t, 1H), 7.41 (td, J = 7.6, 3.5, 1.1 Hz, 1H), 7.32 (t, 6.8 Hz, 1H), 3.70 (p, J = 7.2 Hz, 2H), 0.94 (t, J = 7.1 Hz, 3H); ^{31}P NMR (161 MHz, $CDCl_3$) δ 21.04; IR (film): 3057, 2981, 2903, 2283, 1671, 1475, 1382, 1196, 988, 757 cm^{-1}; HRMS (EI): *m/z* calcd for $C_{14}H_{10}D_5O_3P$: 267.1073; found : 267.1073.

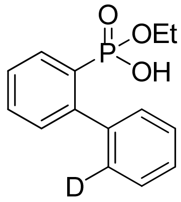

1a-[D$_1$]

2'-deuterio-1,1'-biphenyl phosphonic acid monoethyl ester (1a-D$_1$) : White solid, m.p. 110-112 °C; ^{1}H NMR (400 MHz, $CDCl_3$) δ 11.20 (br, 1H), 8.05 (ddd, J = 14.8, 7.7, 1.1 Hz, 1H), 7.54 (tt, J = 11.4, 1.5 Hz, 1H), 7.46-7.30 (m, 6H), 3.70 (p, J = 7.2 Hz, 2H), 0.94 (t, J = 7.1 Hz, 3H); ^{31}P NMR (161 MHz, $CDCl_3$) δ 20.93; IR (film): 3055, 2981, 2590, 2309, 1670, 1460, 1196, 1040, 988, 754 cm^{-1}; HRMS (EI): *m/z* calcd for $C_{14}H_{14}DO_3P$: 263.0822; found : 263.0818.

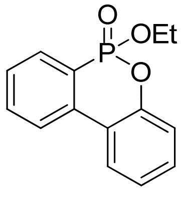

2a

6-Ethoxy-6*H*-dibenz[*c*,*e*]oxaphosphorin-6-oxide (2a) : R_f = 0.4 (EtOAc: hexane = 1:1); Yellow oil (55%, 28.9 mg); ^{1}H NMR (400 MHz, $CDCl_3$) δ 8.00-7.93 (m, 3H), 7.74-7.70 (m, 1H), 7.55-7.50 (m, 1H), 7.41-7.37 (m, 1H), 4.26-4.19 (m, 2H), 1.28 (t, J = 7.1 Hz, 3H); ^{13}C NMR (100 MHz, $CDCl_3$) δ 150.0 (d, J = 7.6 Hz), 137.0 (d, J = 6.8 Hz), 133.4 (d, J = 2.5 Hz), 130.5, 130.2 (d, J = 9.0 Hz), 128.3 (d, J = 15.5 Hz), 125.2, 124.7, 124.0 (d, J = 12.3 Hz), 122.6 (d, J = 11.9 Hz), 122.5 (d, J = 181.8 Hz), 120.2 (d, J = 6.7 Hz), 63.0 (d, J = 6.6 Hz), 16.3 (d, J = 5.8 Hz); ^{31}P NMR (161 MHz, $CDCl_3$) δ 10.14; IR (film): 3065, 2983, 2917, 2359, 1476, 1271, 1240, 1205, 1030, 918, 759 cm^{-1}; HRMS (EI): *m/z* calcd for $C_{14}H_{13}O_3P$: 260.0602; found : 260.0599.

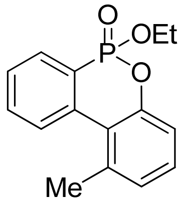

2b

6-Ethoxy-1-methyl-6*H*-dibenz[*c*,*e*]oxaphosphorin-6-oxide (2b) : R_f = 0.4 (EtOAc: hexane = 1:1); Yellow solid (53%, 29.1 mg); m.p. 49-53 ^{o}C; ^{1}H NMR (400 MHz, $CDCl_3$) δ 8.01 (ddd, J = 15.0, 7.5, 1.1 Hz, 1H), 7.84 (t, J = 7.2 Hz, 3H), 7.70-7.66 (m, 1H), 7.52-7.48 (m, 1H), 7.28-7.24 (m, 1H), 7.12 (d, J = 17.9 Hz, 1H), 4.29-4.14 (m, 2H), 2.70 (s, 3H), 1.25 (t, J = 7.1 Hz, 3H); ^{13}C NMR (100 MHz, $CDCl_3$) δ 150.1 (d, J = 8.0 Hz), 137.3 (d, J = 6.4 Hz), 136.7 (d, J = 1.3 Hz), 132.2 (d, J = 2.4 Hz), 130.0 (d, J = 9.8 Hz), 129.2, 128.9 (d, J = 11.8 Hz), 128.5, 127.6 (d, J = 15.6 Hz), 124.9 (d, J = 193.2 Hz), 124.0 (d, J = 2.8 Hz), 117.7 (d, J = 5.9 Hz), 62.9 (d, J = 6.7 Hz), 23.5, 16.3 (d, J = 5.7 Hz); ^{31}P NMR (161 MHz, $CDCl_3$) δ 10.68; IR (film): 3885, 2981, 1576, 1454, 1422, 1275, 1155, 961, 875, 792 cm^{-1}; HRMS (EI): *m/z* calcd for $C_{15}H_{15}O_3P$: 274.0759; found : 274.0761.

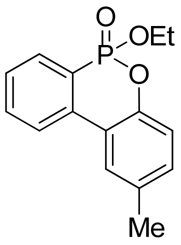

2c

6-Ethoxy-2-methyl-6*H*-dibenz[*c*,*e*]oxaphosphorin-6-oxide (2c) : R_f = 0.4 (EtOAc: hexane = 1:1); Yellow oil (66%, 36.3 mg); ^{1}H NMR (400 MHz, $CDCl_3$) δ 8.00-7.93 (m, 2H), 7.72-7.68 (m, 2H), 7.52-7.48 (m, 1H), 7.18 (d, J = 8.3 Hz, 1H), 7.12 (d, J = 8.3 Hz, 1H), 4.24-4.17 (m, 2H), 2.41 (s, 3H), 1.27 (t, J = 7.1 Hz, 3H); ^{13}C NMR (100 MHz, $CDCl_3$) δ 147.9 (d, J = 7.9 Hz), 137.1 (d, 7.1 Hz), 134.1, 133.3 (d, J = 2.6 Hz), 131.2, 130.1 (d, J = 9.2 Hz), 128.1 (d, J = 15.5 Hz), 125.5 (d, J = 1.0 Hz), 123.9 (d, J = 12.2 Hz), 122.5 (d, J = 181.7 Hz), 122.2 (d, J = 11.9 Hz), 119.9 (d, J = 6.6 Hz), 62.8 (d, J = 6.6 Hz), 21.0, 16.3 (d, J = 5.8 Hz); ^{31}P NMR (161 MHz, $CDCl_3$) δ 10.42; IR (film): 3060, 2983, 2297, 1467, 1272, 1200, 1143, 966, 853, 770 cm^{-1}; HRMS (EI): *m/z* calcd for $C_{15}H_{15}O_3P$: 274.0759; found : 274.0761.

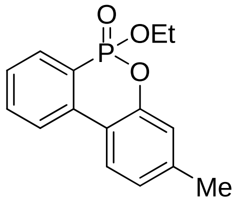

2d

6-Ethoxy-3-methyl-6*H*-dibenz[*c*,*e*]oxaphosphorin-6-oxide (2d) : R_f = 0.4 (EtOAc: hexane = 1:1); Yellow oil (67%, 36.9 mg); ^{1}H NMR (400 MHz, $CDCl_3$) δ 8.00-7.91 (m, 2H), 7.81 (d, J = 8.1 Hz, 1H), 7.70 (t, J = 7.8 Hz, 1H), 7.50-7.46 (m, 1H), 7.08 (d, J = 8.2 Hz, 1H), 7.05 (s, 1H), 4.25-4.17 (m, 2H), 2.40 (s, 3H), 1.28 (t, J = 7.1 Hz, 3H); ^{13}C NMR (100 MHz, $CDCl_3$) δ 149.8 (d, J = 7.8 Hz), 141.2, 137.2 (d, J = 7.1 Hz), 133.4 (d, J = 2.3 Hz), 130.1 (d, J = 9.1 Hz), 127.8 (d, J = 15.4 Hz), 125.6, 124.9, 123.7 (d, J = 12.0 Hz), 122.0 (d, J = 181.4 Hz), 120.5 (d, J = 6.6 Hz), 119.8 (d, J = 12.1 Hz), 62.9 (d, J = 6.6 Hz), 21.2, 16.3 (d, J = 5.9 Hz); ^{31}P NMR (161 MHz, $CDCl_3$) δ 10.50; IR (film): 3542, 2982, 2925, 2357, 1621, 1472, 1272, 966, 853, 770 cm^{-1}; HRMS (EI): *m/z* calcd for $C_{15}H_{15}O_3P$: 274.0759; found : 274.0761.

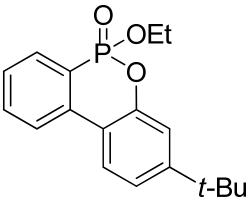

2e

6-Ethoxy-3-*tert*-butyl-6*H*-dibenz[*c*,*e*]oxaphosphorin-6-oxide (2e) : R_f = 0.4 (EtOAc: hexane = 1:1); Yellow oil (65%, 40.9 mg); ^{1}H NMR (400 MHz, $CDCl_3$) δ 7.97-7.92 (m, 2H), 7.85 (d, J = 8.4 Hz, 1H), 7.72-7.67 (m, 1H), 7.51-7.46 (m, 1H), 7.29 (dd, J = 8.4, 1.8 Hz, 1H), 7.23 (d, J = 2.0 Hz, 1H), 4.26-4.19 (m, 2H), 1.35 (s, 9H), 1.29 (t, J = 7.1 Hz, 3H); ^{13}C NMR (100 MHz, $CDCl_3$) δ 154.7, 149.8 (d, J = 7.8 Hz), 137.1 (d, J = 7.2 Hz), 133.3 (d, J = 2.3 Hz), 130.1 (d, J = 9.0 Hz), 127.9 (d, J = 15.4 Hz), 124.7, 123.7 (d, J = 12.3 Hz), 122.1 (d, 181.1 Hz), 121.9, 119.6 (d, J = 11.8 Hz), 117.0 (d, J = 6.8 Hz), 63.0 (d, J = 6.6 Hz), 34.9, 16.3 (d, J = 5.8 Hz); ^{31}P NMR (161 MHz, $CDCl_3$) δ 10.52; IR (film): 2964, 2905, 2359, 1618, 1472, 1272, 1032, 966, 773, 620 cm^{-1}; HRMS (EI): *m/z* calcd for $C_{18}H_{21}O_3P$: 316.1228; found : 316.1231.

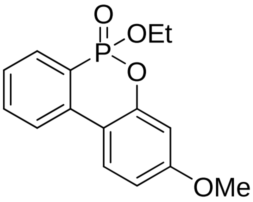

2f

6-Ethoxy-3-methoxy-6*H*-dibenz[*c*,*e*]oxaphosphorin-6-oxide (2f) : R_f = 0.4 (EtOAc: hexane = 2:1); Colorless oil (65%, 38 mg); ^{1}H NMR (400 MHz, $CDCl_3$) δ 7.95 (ddd, J = 14.6, 7.6, 1.1 Hz, 1H), 7.89-7.82 (m, 2H), 7.70-7.66 (m, 1H), 7.47-7.43 (m, 1H), 6.84 (dd, J = 8.8, 2.6 Hz, 1H), 6.76 (d, J = 2.6 Hz, 1H), 4.26-4.18 (m, 2H), 3.87 (s, 3H), 1.29 (t, J = 7.1 Hz, 3H); ^{13}C NMR (100 MHz, $CDCl_3$) δ 161.3, 151.1 (d, J = 7.5 Hz), 137.2 (d, J = 7.1 Hz), 133.4 (d, J = 2.3 Hz), 130.1 (d, J = 9.4 Hz), 127.3 (d, J = 15.5 Hz), 126.1, 123.3 (d, J = 12.3 Hz), 121.1 (d, J = 181.4 Hz), 115.4 (d, J = 12.0 Hz), 111.5, 104.8 (d, J = 7.2 Hz), 62.9 (d, J = 6.5 Hz), 55.7, 16.4 (d, J = 5.9 Hz); ^{31}P NMR (161 MHz, $CDCl_3$) δ 10.83; IR (film): 3063, 2978, 2355, 1619, 1436, 1270, 1034, 771, 618 cm^{-1}; HRMS (EI): *m/z* calcd for $C_{15}H_{15}O_4P$: 290.0708; found : 290.0706.

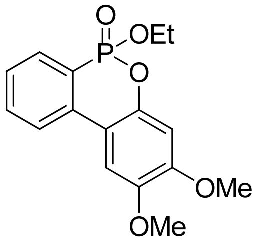

2g

5-Ethoxy-2,3-dimethoxy-6*H*-dibenz[*c*,*e*]oxaphosphorin-5-oxide (2g) : R_f = 0.3 (EtOAc: hexane = 2:1); Yellow solid (68%, 43.5 mg); m.p. 106-111 °C; ^{1}H NMR (400 MHz, $CDCl_3$) δ 7.95 (ddd, *J* = 14.6, 7.5, 1.0 Hz, 1H), 7.83 (t, *J* = 7.2 Hz, 1H), 7.69 (t, *J* = 7.8 Hz, 1H), 7.48-7.43 (m, 1H), 7.32 (s, 1H), 4.25-4.18 (m, 2H), 3.95 (d, *J* = 14.5 Hz, 6H), 1.29 (t, *J* = 7.1 Hz, 3H); ^{13}C NMR (100 MHz, $CDCl_3$) δ 150.9, 146.1, 144.4 (d, *J* = 7.4 Hz), 137.2 (d, *J* = 6.9 Hz), 133.3 (d, *J* = 2.3 Hz), 130.2 (d, *J* = 9.2 Hz), 127.3 (d, *J* = 15.5 Hz), 123.3 (d, *J* = 12.1 Hz), 121.5 (d, *J* = 181.9 Hz), 114.2 (d, *J* = 12.0 Hz), 107.1, 103.5 (d, *J* = 7.2 Hz), 62.9 (d, *J* = 6.5 Hz), 56.3 (d, *J* = 25.5 Hz), 16.4 (d, *J* = 5.8 Hz); ^{31}P NMR (161 MHz, $CDCl_3$) δ 11.03; IR (film): 3478, 2981, 2937, 1617, 1437, 1274, 1153, 1027, 892, 770 cm^{-1}; HRMS (EI): *m/z* calcd for $C_{16}H_{17}O_5P$: 320.0814; found : 320.0810.

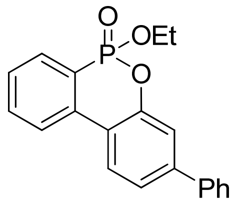

2h

6-Ethoxy-3-phenyl-6*H*-dibenz[*c*,*e*]oxaphosphorin-6-oxide (2h) : R_f = 0.35 (EtOAc: hexane = 1:1); Colorless oil (54%, 36.8 mg); ^{1}H NMR (400 MHz, $CDCl_3$) δ 8.02 (m, 3H), 7.75-7.71 (m, 1H), 7.66-7.63 (m, 2H), 7.55-7.46 (m, 5H), 7.43-7.39 (m, 1H), 4.29-4.21 (m, 2H), 1.30 (t, *J* = 7.1 Hz, 3H); ^{13}C NMR (100 MHz, $CDCl_3$) δ 150.3 (d, *J* = 7.7 Hz), 143.5, 139.2, 136.8 (d, *J* = 7.2 Hz), 133.5 (d, *J* = 2.4 Hz), 130.2 (d, *J* = 9.3 Hz), 129.0, 128.2 (d, *J* = 15.5 Hz), 128.2, 127.0, 125.6, 124.0 (d , *J* = 12.2 Hz), 123.2, 122.3 (d, *J* = 189.9 Hz), 121.4 (d, *J* = 3.6 Hz), 118.4 (d, *J* = 6.7 Hz), 63.0 (d, *J* = 6.6 Hz), 16.4 (d, *J* = 5.8 Hz); ^{31}P NMR (161 MHz, $CDCl_3$) δ 10.37; IR (film): 3060, 2982, 1471, 1272, 1188, 1142, 1029, 946, 760, 697 cm^{-1}; HRMS (EI): *m/z* calcd for $C_{20}H_{17}O_3P$: 336.0915; found : 336.0911.

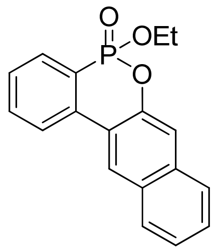

2i

5-Ethoxy-5*H*-benzo[*c*]naphtho[2,3-*e*][1,2]oxaphosphorin-5-oxide (2i) : R_f = 0.35 (EtOAc: hexane = 1:1); Yellow oil (70%, 43.4 mg); ^{1}H NMR (400 MHz, $CDCl_3$) δ 8.40 (s, 1H), 8.13 (t, *J* = 7.2 Hz, 1H), 8.02 (ddd, *J* = 14.6, 7.5, 1.0 Hz, 1H), 7.90 (d, *J* = 8.1 Hz, 1H), 7.80 (d, *J* = 8.1 Hz, 1H), 7.76-7.73 (m, 1H), 4.28-4.20 (m, 2H), 1.26 (t, *J* = 7.1 Hz, 3H); ^{13}C NMR (100 MHz, $CDCl_3$) δ 147.7 (d, *J* = 7.8 Hz), 137.2 (d, *J* = 6.9 Hz), 134.1, 133.5 (d, *J* = 2.3 Hz), 130.5, 130.3 (d, *J* = 9.1 Hz), 128.4, 128.4 (d, *J* = 15.4 Hz), 127.6, 126.9, 125.8, 125.5, 124.6 (d, *J* = 12.2 Hz), 123.1, 122.9 (d, *J* = 11.6 Hz), 116.3 (d, *J* = 6.8 Hz), 63.0 (d, *J* = 6.6 Hz), 16.3 (d, *J* = 5.9 Hz); ^{31}P NMR (161 MHz, $CDCl_3$) δ 20.87; IR (film): 3477, 2983, 2941, 1596, 1431, 1271, 1158, 1030, 876, 770 cm^{-1}; HRMS (EI): *m/z* calcd for $C_{18}H_{15}O_3P$: 310.0759; found : 310.0760.

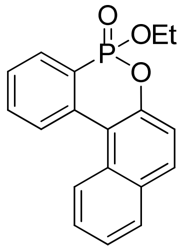

2j

5-Ethoxy-5*H*-benzo[c]naphtho[1,2-e][1,2]oxaphosphorin-5-oxide (2j) : R_f = 0.4 (EtOAc: hexane = 1:1); Yellow oil (52%, 32.2 mg); ^{1}H NMR (400 MHz, $CDCl_3$) δ 8.52 (d, *J* = 8.5 Hz, 1H), 8.17 (t, *J* = 7.0 Hz, 1H), 8.08 (ddd, *J* = 14.8, 7.5, 0.8 Hz, 1H), 7.90 (dd, *J* = 14.3, 8.0 Hz, 2H), 7.74 (t, *J* = 7.8 Hz, 1H), 7.60-7.49 (m, 3H), 7.38 (d, *J* = 8.8 Hz, 1H), 4.30-4.15 (m, 2H), 1.22 (t, *J* = 7.1 Hz, 3H); ^{13}C NMR (100 MHz, $CDCl_3$) δ 147.6 (d, *J* = 8.0 Hz), 136.8 (d, *J* = 6.0 Hz), 132.5 (d, *J* = 2.3 Hz), 131.8, 131.2, 130.4 (d, *J* = 9.8 Hz), 130.4, 129.4 (d, *J* = 11.8 Hz), 128.9, 127.8 (d, *J* = 16.0 Hz), 127.4, 125.6, 125.3, 123.7, 119.4 (d, *J* = 5.9 Hz), 119.1 (d, *J* = 12.1 Hz), 63.0 (d, *J* = 6.8 Hz), 16.3 (d, *J* = 5.6 Hz); ^{31}P NMR (161 MHz, $CDCl_3$) δ 10.07;

IR (film): 3726, 3061, 2982, 2926, 2358, 1618, 1380, 1275, 1027, 875, 729 cm^{-1}; HRMS (EI): *m/z* calcd for $C_{18}H_{15}O_3P$: 310.0759; found : 310.0756.

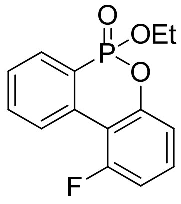

2k

6-Ethoxy-1-floro-6*H*-dibenz[*c*,*e*]oxaphosphorin-6-oxide (2k) : R_f = 0.4 (EtOAc: hexane = 1:1); Yellow solid (54%, 29.8 mg); m.p. 72-78 °C; ^{1}H NMR (400 MHz, $CDCl_3$) δ 8.27 (t, *J* = 7.3 Hz, 1H), 8.04-8.00 (m, 1H), 7.72 (t, *J* = 7.8 Hz, 1H), 7.57-7.52 (m, 1H), 7.09-7.01 (m, 2H), 4.28-4.20 (m, 2H), 1.28 (t, *J* = 7.1 Hz, 3H); ^{13}C NMR (100 MHz, $CDCl_3$) δ 160.6 (d, *J* = 254.9 Hz), 150.6 (t, *J* = 7.0 Hz), 133.7 (dd, *J* = 6.2, 4.1 Hz), 133.4, 130.0, 129.9 (d, *J* = 3.7 Hz), 128.6 (d, *J* = 4.2 Hz), 128.4, 128.4 (d, *J* = 19.4 Hz), 123.0 (d, *J* = 182.7 Hz), 116.0 (dd, *J* = 6.3, 3.3 Hz), 112.8 (d, *J* = 24.6 Hz), 63.2 (d, *J* = 6.6 Hz), 16.3 (d, *J* = 5.8 Hz); ^{31}P NMR (161 MHz, $CDCl_3$) δ 9.87; IR (film): 3063, 2983, 1619, 1433, 1277, 1221, 1010, 796, 609 cm^{-1}; HRMS (EI): *m/z* calcd for $C_{14}H_{12}FO_3P$: 278.0508; found : 278.0509.

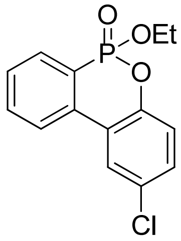

2l

6-Ethoxy-2-chloro-6*H*-dibenz[*c*,*e*]oxaphosphorin-6-oxide (2l) : R_f = 0.4 (EtOAc: hexane = 1:1); White solid (60%, 35.2 mg); m.p. 110-113 °C; ^{1}H NMR (400 MHz, $CDCl_3$) δ 8.00 (ddd, *J* = 14.6, 7.6, 1.1 Hz, 1H), 7.94-7.90 (m, 2H), 7.76-7.72 (m, 1H), 7.59-7.52 (m, 1H), 7.36-7.33 (m, 1H), 7.19 (d, *J* = 8.6 Hz, 1H), 4.27-4.20 (m, 2H), 1.29 (t, *J* = 7.1 Hz, 3H); ^{13}C NMR (100 MHz, $CDCl_3$) δ 148.4 (d, *J* = 7.9 Hz), 135.8 (d, *J* = 7.0 Hz), 133.6 (d, *J* = 2.4 Hz), 130.2, 130.2, 130.1, 130.0 (d, *J* = 28.0 Hz), 129.0 (d, *J* = 15.3 Hz), 125.2 (d, *J* = 1.0 Hz), 124.1 (d, *J* = 12.0 Hz), 123.7 (d, *J* = 60.1 Hz), 121.6 (d, *J* = 6.7 Hz), 63.2 (d, *J* = 6.6 Hz), 16.3 (d, *J* = 5.8

Hz); ^{31}P NMR (161 MHz, $CDCl_3$) δ 9.87; IR (film): 3060, 2981, 1596, 1243, 1111, 1025, 966, 789, 659 cm^{-1}; HRMS (EI): *m/z* calcd for $C_{14}H_{12}ClO_3P$: 294.0213; found : 294.0210.

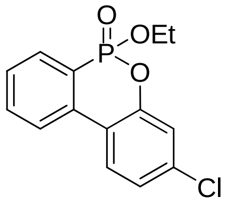

2m

6-Ethoxy-3-chloro-6*H*-dibenz[*c*,*e*]oxaphosphorin-6-oxide (2m) : R_f = 0.4 (EtOAc: hexane = 1:1); White solid (64%, 37.5 mg); m.p. 105-112 °C; ^{1}H NMR (400 MHz, $CDCl_3$) δ 8.00 (ddd, *J* = 14.7, 7.6, 1.0 Hz, 1H), 7.92 (t, *J* = 7.2 Hz, 1H), 7.90 (d, *J* = 9.1 Hz, 1H), 7.75-7.70 (m, 1H), 7.57-7.52 (m, 1H), 7.27-7.25 (m, 2H), 4.29-4.20 (m, 2H), 1.30 (t, *J* = 7.1 Hz, 3H); ^{13}C NMR (100 MHz, $CDCl_3$) δ 150.2 (d, *J* = 7.6 Hz), 136.1 (d, *J* = 6.8 Hz), 135.6, 133.6 (d, *J* = 2.3 Hz), 130.3 (d, *J* = 9.3 Hz), 128.6 (d, *J* = 15.4 Hz), 126.2 (d, *J* = 1.0 Hz), 125.1, 124.0 (d, *J* = 11.9 Hz), 122.2 (d, *J* = 170.0 Hz), 121.3, 120.4 (d, *J* = 7.0 Hz), 63.2 (d, *J* = 6.6 Hz), 16.3 (d, *J* = 5.8 Hz); ^{31}P NMR (161 MHz, $CDCl_3$) δ 10.00; IR (film): 3068, 2982, 1601, 1470, 1273, 1081, 1028, 943, 768, 618 cm^{-1}; HRMS (EI): *m/z* calcd for $C_{14}H_{12}ClO_3P$: 294.0213; found : 294.0210.

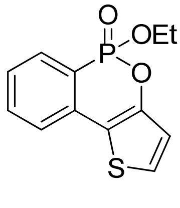

2n

5-Ethoxy-5*H*-benzo[*c*]thieno[2,3-*e*][1,2]oxaphosphorin-5-oxide (2n) : R_f = 0.4 (EtOAc: hexane = 1:1); Yellow solid (52%, 27.8 mg); m.p. 69-73 °C; ^{1}H NMR (400 MHz, $CDCl_3$) δ 7.92 (ddd, *J* = 14.1, 7.6, 0.7 Hz, 1H), 7.64-7.60 (m, 1H), 7.51-7.47 (m, 1H), 7.44-7.39 (m, 1H), 7.26-7.25 (m, 1H), 4.27-4.19 (m, 2H), 1.32 (t, *J* = 7.1 Hz, 3H); ^{13}C NMR (100 MHz, $CDCl_3$) δ 147.5 (d, *J* = 7.7 Hz), 134.5 (d, *J* = 7.3 Hz), 133.6 (d, *J* = 2.3 Hz), 130.4 (d, *J* = 9.4 Hz), 127.4 (d, *J* = 15.4 Hz), 124.4, 123.1 (d, *J* = 11.0 Hz), 120.3 (d, *J* = 6.6 Hz), 118.5 (d, *J* = 180.5 Hz), 118.5 (d, *J* = 13.6 Hz), 63.1 (d, *J* = 6.6 Hz), 16.3 (d, *J* = 6.0 Hz); ^{31}P NMR (161

MHz, $CDCl_3$) δ 13.26; IR (film): 3082, 2982, 2357, 1476, 1272, 1034, 1006, 765, 638 cm^{-1}; HRMS (EI): *m/z* calcd for $C_{12}H_{11}O_3PS$: 266.0167; found : 266.0167.

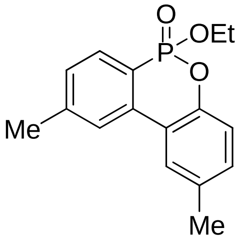

2o

6-Ethoxy-2,9-dimethyl-6*H*-dibenz[*c*,*e*]oxaphosphorin-6-oxide (4a) : R_f = 0.4 (EtOAc: hexane = 1:1); Yellow solid (72%, 41.3 mg); m.p. 112-120 °C; ^{1}H NMR (400 MHz, $CDCl_3$) δ 7.86 (dd, *J* = 14.4, 7.7 Hz, 1H), 7.76 (d, *J* = 6.0 Hz, 1H), 7.71 (d, *J* = 1.5 Hz, 1H), 7.33-7.30 (m, 1H), 7.17 (d, *J* = 8.3 Hz, 1H), 7.10 (d, *J* = 8.2 Hz, 1H), 4.21-4.14 (m, 2H), 2.50 (s, 3H), 2.41 (s, 3H), 1.26 (t, *J* = 7.1 Hz, 3H); ^{13}C NMR (100 MHz, $CDCl_3$) δ 148.0 (d, *J* = 7.9 Hz), 143.9 (d, *J* = 2.8 Hz), 137.1 (d, *J* = 7.3 Hz), 134.0, 131.0 130.2 (d, *J* =9.6 Hz), 129.1 (d, *J* = 16.0 Hz), 125.4, 124.5 (d, *J* = 12.5 Hz), 122.2 (d, *J* = 11.8 Hz), 119.8 (d, *J* = 6.6 Hz), 119.4 (d, *J* = 184.1 Hz), 62.7 (d, *J* = 6.6 Hz), 22.1, 21.0, 16.3 (d, *J* = 5.9 Hz); ^{31}P NMR (161 MHz, $CDCl_3$) δ 11.25; IR (film): 3047, 2981, 2357, 1497, 1271, 1216, 1032, 938, 748, 642 cm^{-1}; HRMS (EI): *m/z* calcd for $C_{16}H_{17}O_3P$: 288.0915; found : 288.0913.

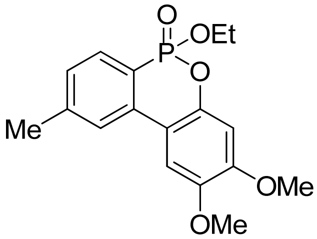

2p

6-Ethoxy-2,3-dimethoxy-9-methyl-6*H*-dibenz[*c*,*e*]oxaphosphorin-6-oxide (4b) : R_f = 0.3 (EtOAc: hexane = 2:1); Yellow solid (70%, 46.7 mg); m.p. 126-130 °C; ^{1}H NMR (400 MHz, $CDCl_3$) δ 7.83 (dd, *J* = 14.4, 7.7 Hz, 1H), 7.61 (d, *J* = 6.0 Hz, 1H), 7.31 (s, 1H), 7.28-7.26 (m, 1H), 6.74 (s, 1H), 4.23-4.15 (m, 2H), 3.99 (s, 3H), 3.93 (s, 3H), 2.50 (s, 3H), 1.28 (t, *J* = 7.1 Hz, 3H); ^{13}C NMR (100 MHz, $CDCl_3$) δ 150.8, 146.0, 144.6 (d, *J* = 7.5 Hz), 143.9 (d, *J* = 7.5

Hz), 143.9 (d, J = 2.6 Hz), 137.1 (d, J = 7.5 Hz), 130.2 (d, J = 9.6 Hz), 128.3 (d, J = 16.0 Hz), 123.7 (d, J = 12.5 Hz), 118.4 (d, J = 184.2 Hz), 114.2 (d, J = 11.9 Hz), 107.0, 103.4, (d, J = 7.1 Hz), 62.8 (d, J = 6.5 Hz), 56.5, 56.2, 22.5, 16.4 (d, J = 5.9 Hz); ^{31}P NMR (161 MHz, $CDCl_3$) δ 11.84; IR (film): 2977, 2937, 2240, 1520, 1272, 1214, 1038, 913, 733, 648 cm^{-1}; HRMS (EI): *m/z* calcd for $C_{17}H_{19}O_5P$: 334.0970; found : 334.0969.

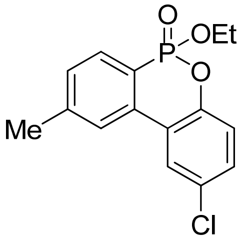

2q

6-Ethoxy-2-chloro-9-methyl-6*H*-dibenz[*c*,*e*]oxaphosphorin-6-oxide (4c) : R_f = 0.35 (EtOAc: hexane = 1:1); Yellow solid (64%, 39.7 mg); m.p. 97-100 °C; ^{1}H NMR (400 MHz, $CDCl_3$) δ 7.90-7.84 (m, 2H), 7.71 (d, J = 6.0 Hz, 1H), 7.39-7.36 (m, 1H), 7.17 (d, J = 8.7 Hz, 1H), 4.25-4.17 (m, 2H), 2.51 (s, 3H), 1.28 (t, J = 7.1 Hz, 3H); ^{13}C NMR (100 MHz, $CDCl_3$) δ 148.6 (d, J = 7.9 Hz), 144.3 (d, J = 2.4 Hz), 135.8 (d, J = 7.3 Hz), 130.3 (d, J = 9.5 Hz), 130.0, 129.9 (d, J = 15.9 Hz), 129.8, 125.1, 124.6 (d, J = 12.4 Hz), 121.5 (d, J = 6.7 Hz), 119.4 (d, J = 184.7 Hz), 63.1 (d, J = 6.6 Hz), 22.1, 16.3 (d, J = 5.9 Hz); ^{31}P NMR (161 MHz, $CDCl_3$) δ 10.67; IR (film): 3065, 2982, 2355, 1619, 1436, 1270, 1034, 771, 618 cm^{-1}; HRMS (EI): *m/z* calcd for $C_{15}H_{14}ClO_3P$: 308.0369; found : 308.0371.

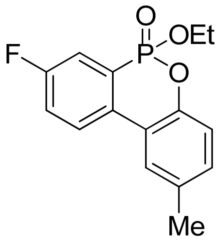

2r

6-Ethoxy-2-methyl-8-floro-6*H*-dibenz[*c*,*e*]oxaphosphorin-6-oxide (4e) : R_f = 0.35 (EtOAc: hexane = 1:1); White solid (57%, 33.4 mg); m.p. 102-105 °C; ^{1}H NMR (400 MHz, $CDCl_3$) δ 7.98-7.92 (m, 1H), 7.68-7.61 (m, 2H), 7.38 (td, J = 12.8, 2.8 Hz, 1H), 7.18 (d, J = 8.3 Hz, 1H), 7.12 (d, J = 8.3 Hz, 1H), 4.26-4.19 (m, 2H), 2.41 (s, 3H), 1.29 (t, J = 7.1 Hz, 3H); ^{13}C

NMR (100 MHz, $CDCl_3$) δ 161.9 (dd, J = 21.8, 252.1 Hz), 147.4 (d, J = 8.0 Hz), 134.4, 133.4 (dd, J = 6.4, 3.6 Hz), 131.1, 126.6 (dd, J = 14.3, 7.6 Hz), 125.4, 124.6 (dd, J = 182.0, 6.6 Hz), 121.6, 120.9 (dd, J = 21.8, 2.7 Hz), 119.9 (d, J = 6.8 Hz), 116.7 (dd, J = 22.8, 9.9 Hz), 63.2 (d, J = 6.6 Hz), 21.0, 16.3 (d, J = 5.8 Hz); ^{31}P NMR (161 MHz, $CDCl_3$) δ 8.56 (d, J = 7.9 Hz); IR (film): 3539, 3063, 2983, 2926, 1721, 1608, 1488, 1259, 1028, 828 cm^{-1}; HRMS (EI): *m/z* calcd for $C_{15}H_{14}FO_3P$: 292.0665; found : 292.0663.

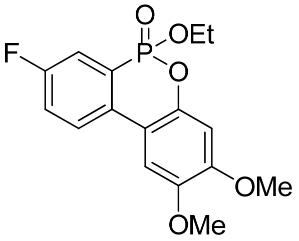

2s

6-Ethoxy-2,3-dimethoxy-8-floro-6*H*-dibenz[*c*,*e*]oxaphosphorin-6-oxide (4f) : R_f = 0.30 (EtOAc: hexane = 2:1); Yellow solid (63%, 42.7 mg); m.p. 126-130 °C; ^{1}H NMR (400 MHz, $CDCl_3$) δ 7.85-7.80 (m, 1H), 7.63 (ddd, J = 15.7, 7.6, 2.7 Hz, 1H), 7.38 (td, J = 12.8, 2.6 Hz, 1H), 7.26 (s, 1H), 6.75 (s, 1H), 4.28-4.20 (m, 2H), 4.00 (s, 3H), 3.93 (s, 3H), 1.31 (t, J = 7.1 Hz, 3H); ^{13}C NMR (100 MHz, $CDCl_3$) δ 161.4 (dd, J =21.8, 251.4 Hz), 150.9, 146.3, 143.9 (d, J = 7.6 Hz), 133.5 (dd, J = 6.1, 3.5 Hz), 125.8 (dd, J = 14.2, 7.5 Hz), 123.5 (dd, J = 182.4, 6.5 Hz), 120.8 (dd, J = 22.0, 2.8 Hz), 116.7 (dd, J = 22.8, 9.7 Hz), 113.6 (d, J = 11.8 Hz), 107.0, 103.5 (d, J = 7.1 Hz), 63.2 (d, J = 6.5 Hz), 56.4 (d, J = 24.9 Hz), 16.4 (d, J = 5.7 Hz); ^{31}P NMR (161 MHz, $CDCl_3$) δ 9.15 (d, J = 7.7 Hz); IR (film): 3065, 2981, 2245, 1618, 1488, 1273, 1241, 1028, 895, 737 cm^{-1}; HRMS (EI): *m/z* calcd for $C_{16}H_{16}FO_5P$: 338.0719; found : 338.0721.

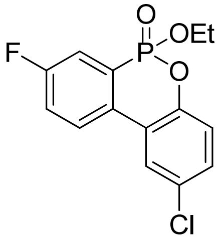

2t

6-Ethoxy-2-chloro-8-floro-6*H*-dibenz[*c*,*e*]oxaphosphorin-6-oxide (2t) : R_f = 0.40 (EtOAc: hexane = 1:1); White solid (50%, 31.3 mg); m.p. 127-132 °C; ^{1}H NMR (400 MHz, $CDCl_3$) δ

7.94-7.89 (m, 1H), 7.84 (d, J = 2.4 Hz, 1H), 7.68 (ddd, J = 15.7, 10.2, 2.8 Hz, 1H), 7.42 (td, J = 12.8, 2.8 Hz, 1H), 7.36-7.33 (m, 1H), 7.20 (d, J = 8.7 Hz, 1H), 4.30-4.22 (m, 2H), 1.31 (t, J = 7.1 Hz, 3H); ^{13}C NMR (100 MHz, $CDCl_3$) δ 162.4 (dd, J = 21.4, 253.8 Hz), 147.9 (d, J = 8.2 Hz), 132.0 (dd, J = 6.0, 3.6 Hz), 130.3, 130.1, 126.9 (dd, J = 14.2, 7.8 Hz), 125.0, 124.8 (dd, J = 182.6, 6.6 Hz), 123.5 (d, J = 11.6 Hz), 121.2, 121.1 (dd, J = 22.0, 2.7 Hz), 116.9 (dd, J = 23.0, 9.7 Hz), 63.5 (d, J = 6.6 Hz), 16.3 (d, J = 5.8 Hz); ^{31}P NMR (161 MHz, $CDCl_3$) δ 7.96 (d, J = 7.9 Hz); IR (film): 3568, 3069, 2984, 2315, 1725, 1606, 1477, 1255, 1027, 829 cm^{-1}; HRMS (EI): m/z calcd for $C_{14}H_{11}ClFO_3P$: 312.0118; found : 312.0117.

1.7. Studies with isotopically labelled compounds

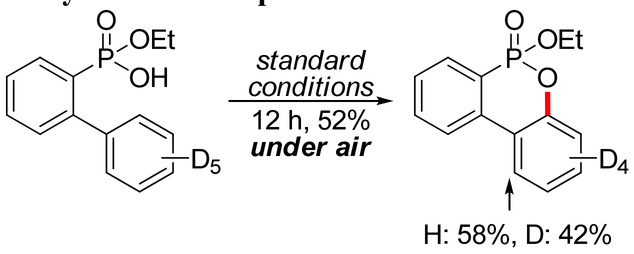

1a-[D$_5$] **2a-[D$_4$]**

2',3',4',5',6'-Pentadeuterio-1,1'-biphenyl phosphonic acid monoethyl ester **1a-[D$_5$]** (53.4 mg, 0.2 mmol), $Pd(OAc)_2$ (4.49 mg, 0.02 mmol), *N*-acetyl-L-leucine (10.4 mg, 0.06 mmol), $PhI(OAc)_2$ (128.8 mg, 0.4 mmol), KOAc (39.3 mg, 0.4 mmol) and *t*-BuOH (2.5 mL) were added in a 2.5 mL screw-top v-vial. The reaction mixture was stirred at 100 ^{o}C for 12 h under air atmosphere. After celite filtration and evaporation of the solvents in vacuo, the crude product was purified by column chromatography on silica gel (EtOAc/hexane = 1/1) to yield **2a-[d$_4$]** (27.6 mg,) as a yellow oil.

1.8. Intermolecular competition

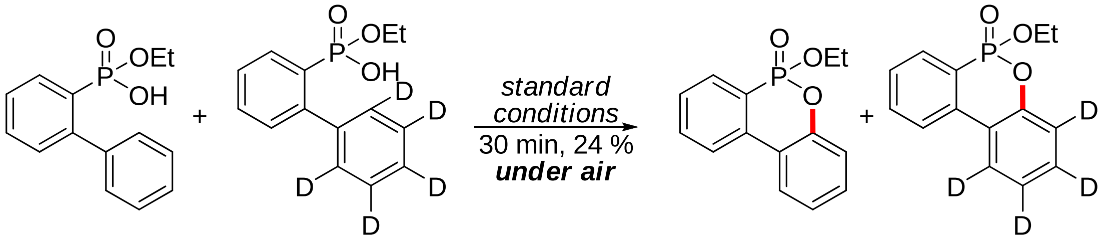

1a **1a-[D$_5$]** **2a** **2a-[D$_4$]**

k_H/k_D = 1.0

1,1'-Biphenylphosphonic acid monoethyl ester **1a** (26.2mg, 0.10 mmol) and 2',3',4',5',6'-pentadeuterio-1,1'-biphenylphosphonic acid monoethyl ester **1a-[D$_5$]** (26.7 mg, 0.1 mmol), $Pd(OAc)_2$ (4.49 mg, 0.02 mmol), *N*-acetyl-L-leucine (10.4 mg, 0.06 mmol), $PhI(OAc)_2$ (128.8 mg, 0.4 mmol), KOAc (39.3 mg, 0.4 mmol) and *t*-BuOH (2.5 mL) were added in a 2.5 mL screw-top v-vial. The reaction mixture was stirred at 100 ^oC for 30 min under air atmosphere. After celite filtration and evaporation of the solvents in vacuo, the crude product was purified by column chromatography on silica gel (EtOAc/hexane = 1/1) to yield **2a** and **2a-[d$_4$]** (12.4 mg, 24%) as a yellow oil. The ratio of **2a** and **2a-[D$_4$]** was determined to be 0.5 / 0.5 (KIE = 1.0) by ^{1}H-NMR spectroscopy.

1.9. Intramolecular competition

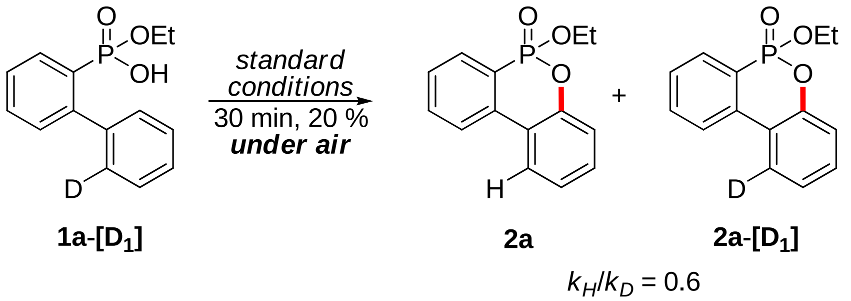

2'-Deuterio-1,1'-biphenylphosphonic acid monoethyl ester **1a-[D$_1$]** (52.7 mg, 0.20 mmol), $Pd(OAc)_2$ (4.49 mg, 0.02 mmol), *N*-acetyl-L-leucine (10.4 mg, 0.06 mmol), $PhI(OAc)_2$ (128.8 mg, 0.4 mmol), KOAc (39.3 mg, 0.4 mmol) and *t*-BuOH (2.5 mL) were added in a 2.5 mL screw-top v-vial. The reaction mixture was stirred at 100 ^oC for 30 min under air atmosphere. After celite filtration and evaporation of the solvents in vacuo, the crude product was purified by column chromatography on silica gel (EtOAc/hexane = 1/1) to yield **2a** and **2a-[d$_1$]** (10.3 mg, 20%) as a yellow oil. The ratio of **2a** / **2a-[D$_1$]** was determined to be 0.375 / 0.625 (KIE = 0.6) by ^{1}H-NMR spectroscopy.

2. References

1. Zhang, H.-Y.; Yi, H.-M.; Wang, G.-W.; Wang, B.; Yang, S.-D. *Org. Lett.* **2013,** *15,* 6186. doi:10.1021/ol403028a

2. Bonnaventure, I.; Charette, A. B. *J. Org. Chem.* **2008,** *73,* 6330. doi:10.1021/jo800969x

3. Chowdhury, S.; Muni, N. J.; Greenwood, N. P.; Pepperberg, D. R.; Standaert, R. F. *Bioorg.*

Med. Chem. Lett. **2007,** *17,* 3745. doi:10.1016/j.bmcl.2007.04.026

4. Hu, R.-B.; Zhang, H.; Zhang, X.-Y.; Yang, S.-D. *Chem. Commun.* **2014,** *50,* 2193. doi:10.1039/c3cc49050e

5. Becht, J.-M.; Ngouela, S.; Wagner, A.; Mioskowski, C. *Tetrahedron* **2004,** *60,* 6583.

6. Ahmed, A.; Dhara, S.; Ray, J. K. *Tetrahedron Lett.* **2013,** *54,* 1673. doi:10.1016/j.tetlet.2013.01.062

7. Leroux, F. R.; Bonnafoux, L.; Heiss, C.; Colobert, F.; Lanfranchi, D. A. *Adv. Synth. Catal.* **2007,** *349,* 2705. doi:10.1002/adsc.200700211

3. ^{1}H and ^{13}C NMR spectra

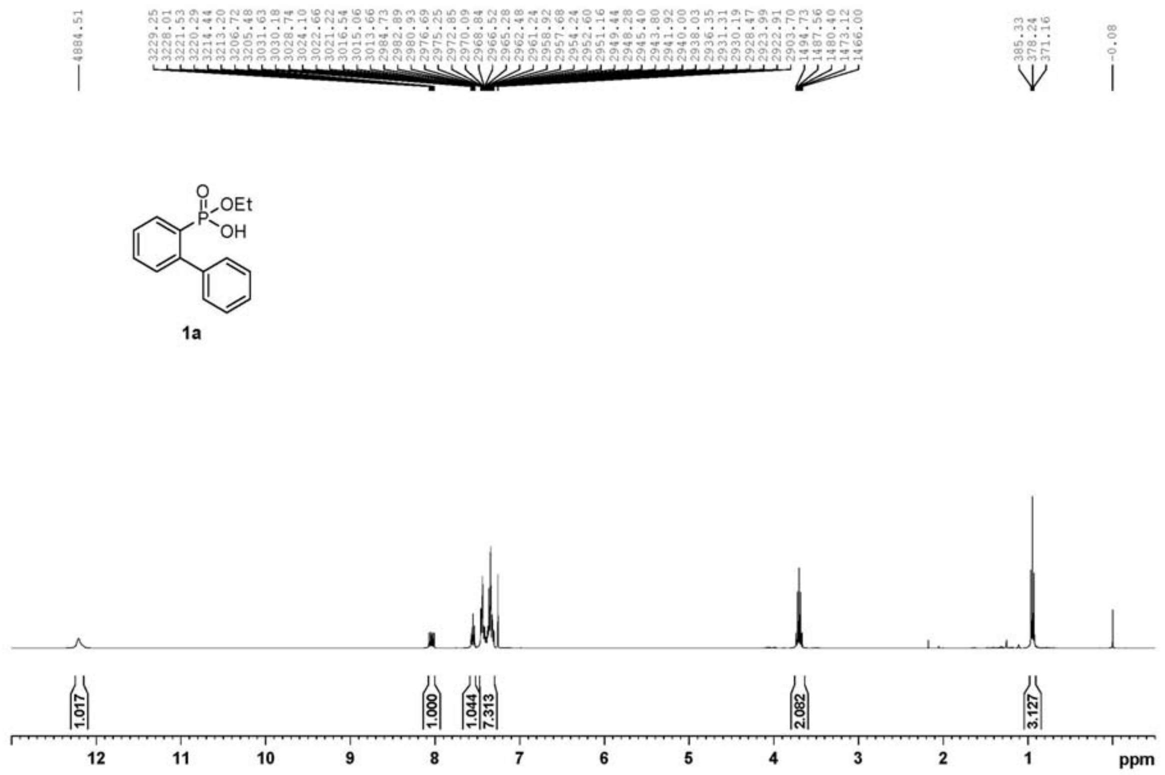

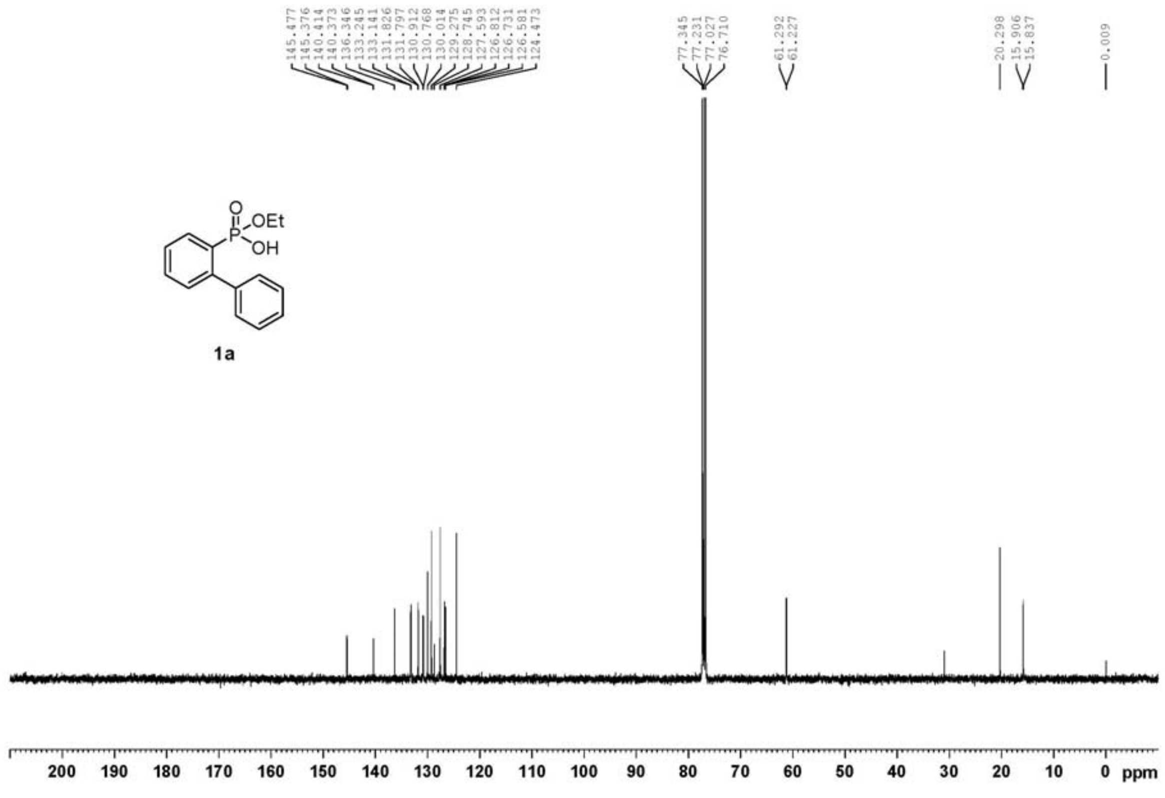

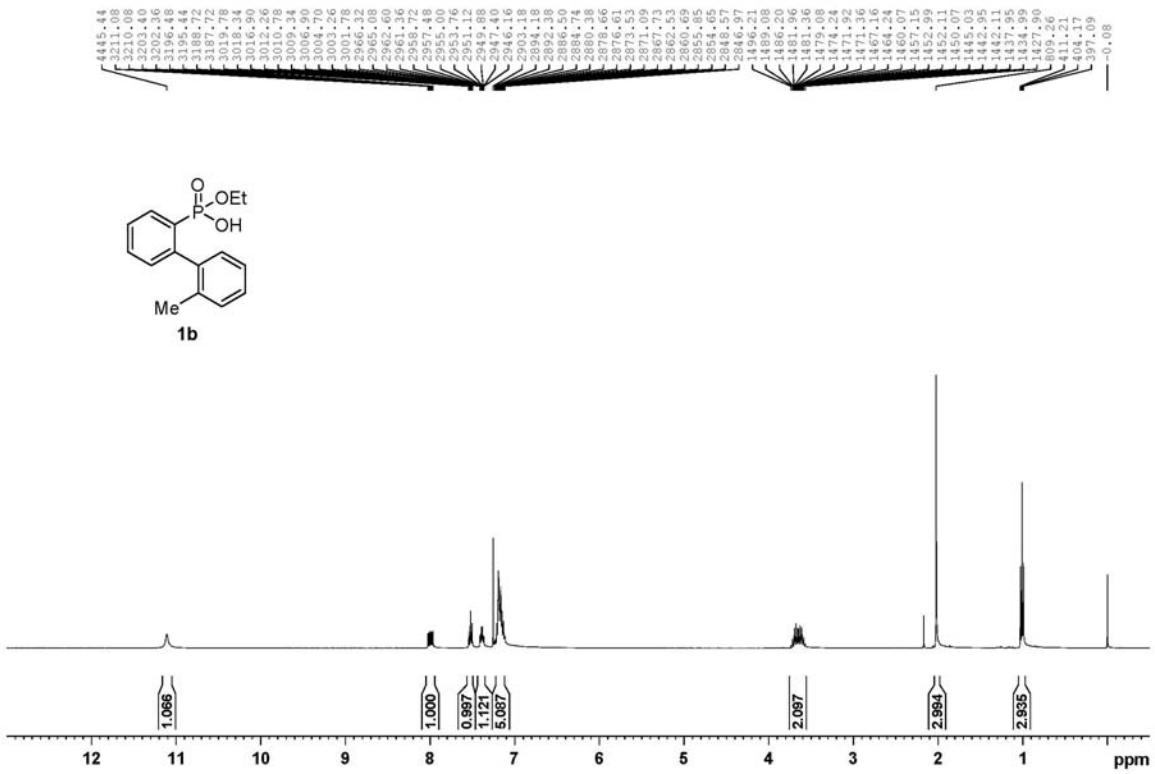
4445.44
3211.08
3210.08
3203.40
3202.36
3196.48
3195.44
3188.72
3187.72
3019.78
3018.34
3016.90
3012.26
3010.78
3009.34
3006.90
3004.70
3003.26
3001.78
2966.32
2965.08
2962.60
2961.36
2958.72
2957.48
2955.00
2953.76
2951.12
2949.88
2947.40
2946.16
2903.18
2894.18
2892.38
2886.50
2884.74
2880.38
2878.66
2876.61
2873.53
2871.09
2867.73
2862.53
2860.69
2855.85
2854.65
2848.57
2846.97
1496.21
1489.08
1486.20
1481.96
1481.36
1479.08
1474.24
1471.92
1471.36
1467.16
1464.24
1460.07
1457.15
1452.99
1452.11
1450.07
1445.03
1442.95
1442.11
1437.95
1434.99
1427.90
809.26
411.21
404.17
397.09
-0.08
O
P
OEt
OH
Me
1b
1.066
1.000
0.997
1.121
5.087
2.097
2.994
2.935
12
11
10
9
8
7
6
5
4
3
2
1
ppm

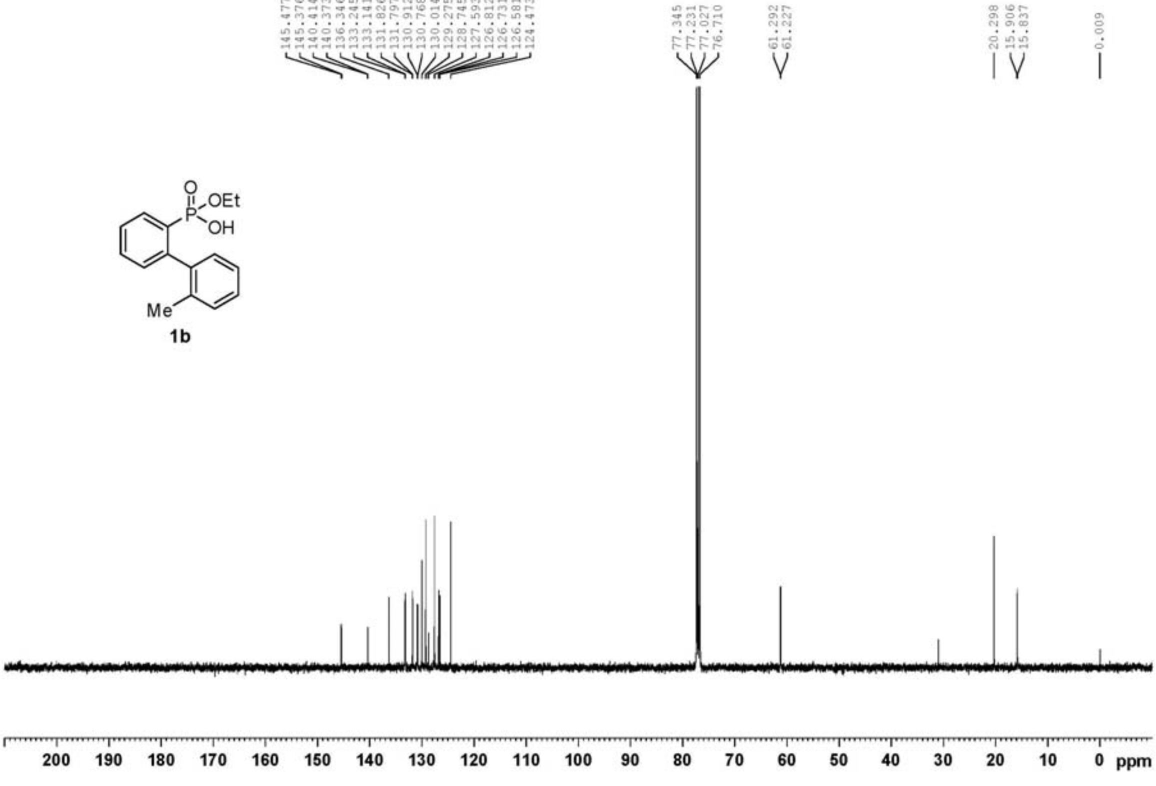
145.477
145.376
140.414
140.373
136.346
133.245
133.141
131.826
131.797
130.912
130.768
130.014
129.275
128.745
127.593
126.812
126.731
126.581
124.473
77.345
77.231
77.027
76.710
61.292
61.227
20.298
15.906
15.837
0.009
O
P
OEt
OH
Me
1b
200
190
180
170
160
150
140
130
120
110
100
90
80
70
60
50
40
30
20
10
0
ppm

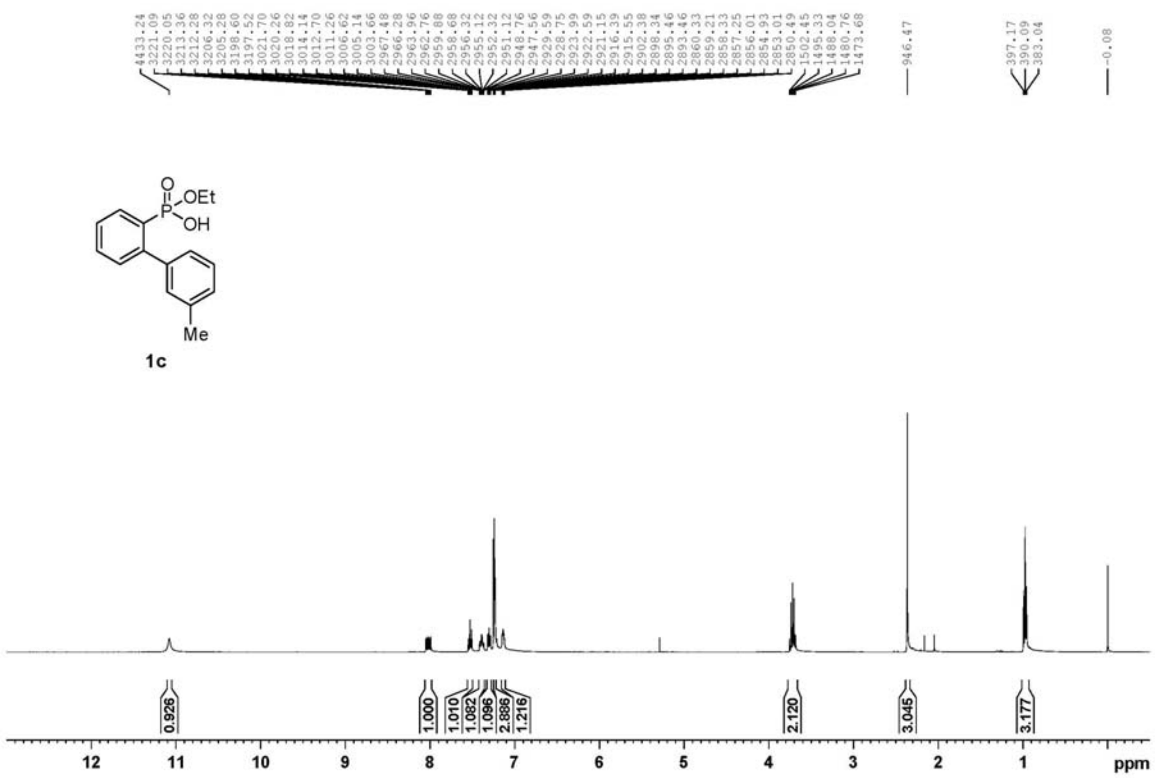
4433.24
3221.09
3220.05
3213.36
3212.28
3206.32
3205.28
3198.60
3197.52
3021.70
3020.26
3018.82
3014.14
3012.70
3011.26
3006.62
3005.14
3003.66
2967.48
2966.28
2963.96
2962.76
2959.88
2958.68
2956.32
2955.12
2952.32
2951.12
2948.76
2947.56
2929.59
2928.75
2923.99
2922.59
2921.15
2916.39
2915.55
2902.38
2898.34
2895.46
2893.46
2860.33
2859.21
2858.33
2857.25
2856.01
2854.93
2853.01
2850.49
1502.45
1495.33
1488.04
1480.76
1473.68
946.47
397.17
390.09
383.04
-0.08
OEt
OH
Me
1c
0.926
1.000
1.010
1.082
1.096
2.886
1.216
2.120
3.045
3.177
12
11
10
9
8
7
6
5
4
3
2
1
ppm

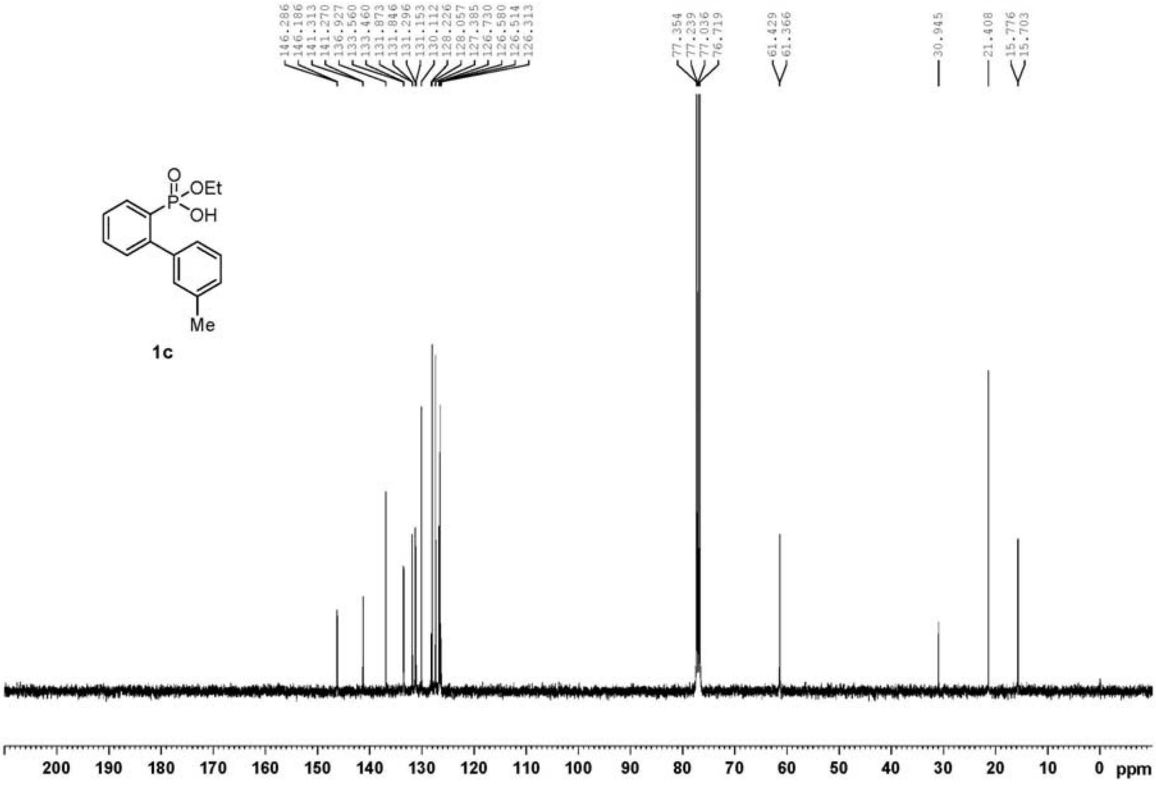
146.286
146.186
141.313
141.270
136.927
133.560
133.460
131.873
131.846
131.296
131.153
130.112
128.226
128.057
127.385
126.730
126.580
126.514
126.313
77.354
77.239
77.036
76.719
61.429
61.366
30.945
21.408
15.776
15.703
OEt
OH
Me
1c
200
190
180
170
160
150
140
130
120
110
100
90
80
70
60
50
40
30
20
10
0
ppm

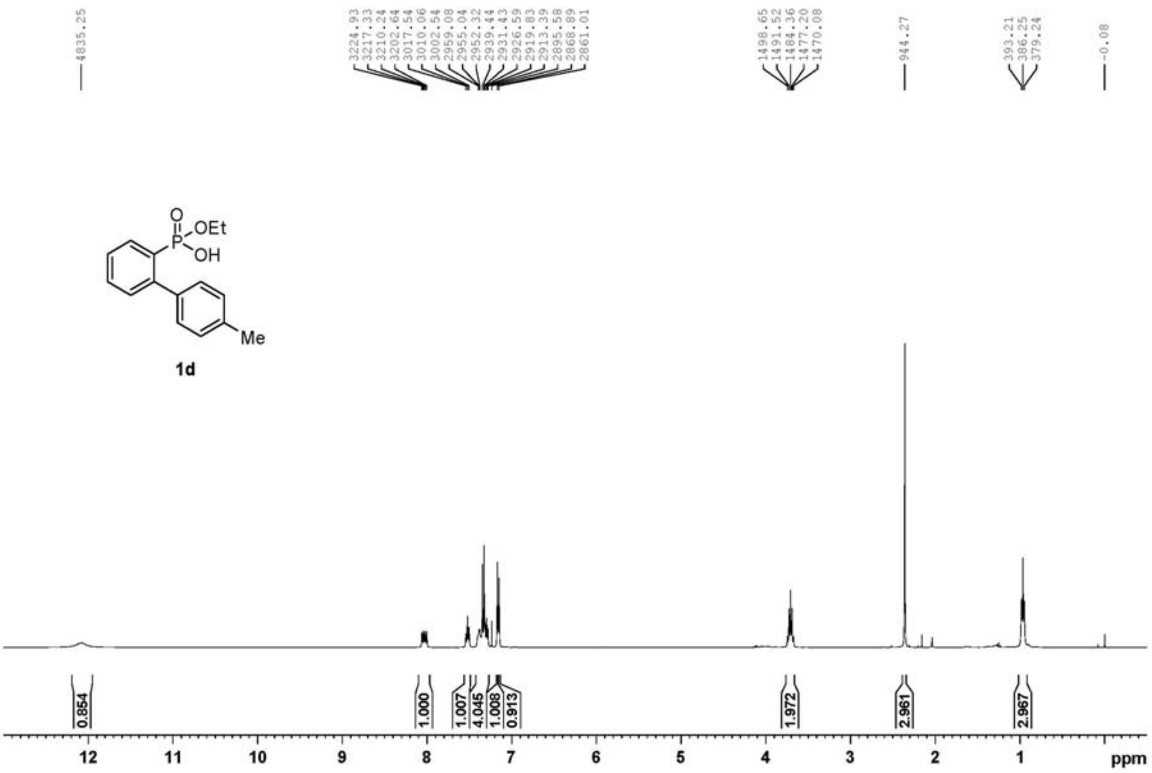
OEt
OH
Me
1d
ppm

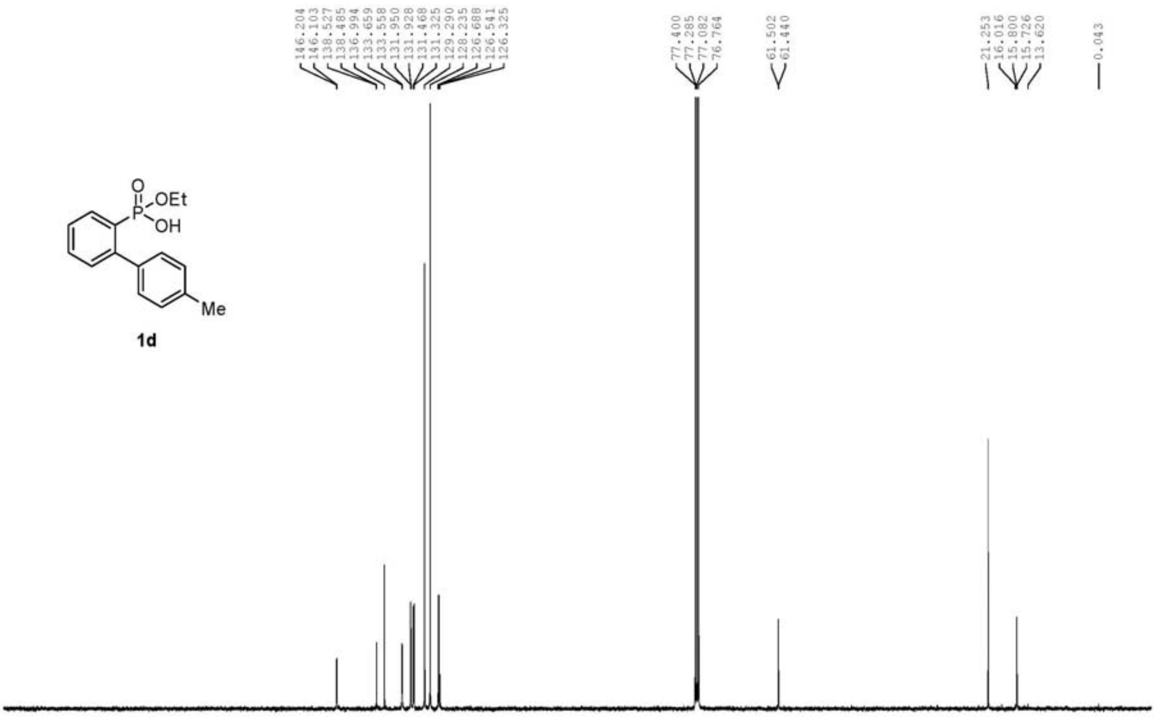
OEt
OH
Me
1d
ppm

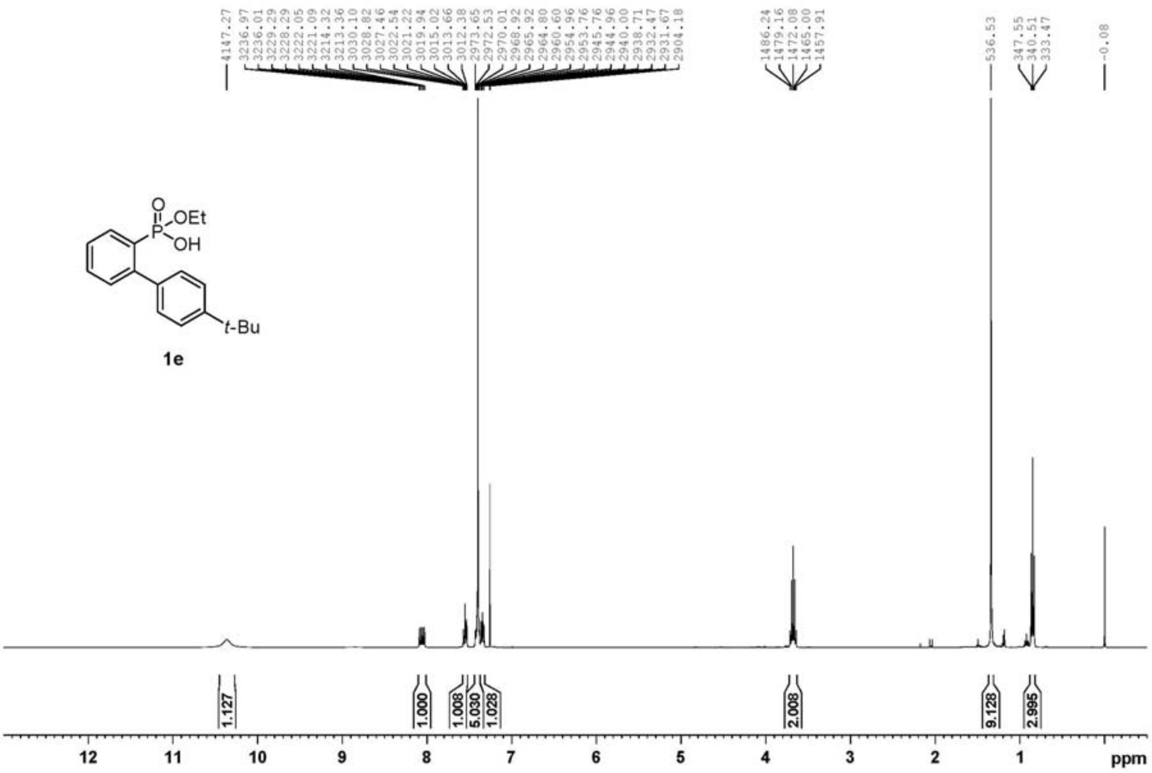
4147.27
3236.97
3236.01
3229.29
3228.29
3222.05
3221.09
3214.32
3213.36
3030.10
3028.82
3027.46
3022.54
3021.22
3019.94
3015.02
3013.66
3012.38
2973.65
2972.53
2970.01
2968.92
2965.92
2964.80
2960.60
2954.96
2953.76
2945.76
2944.96
2940.00
2938.71
2932.47
2931.67
2904.18
1486.24
1479.16
1472.08
1465.00
1457.91
536.53
347.55
340.51
333.47
-0.08
OEt
OH
t-Bu
1e
1.127
1.000
1.008
5.030
1.028
2.008
9.128
2.995
12
11
10
9
8
7
6
5
4
3
2
1
ppm

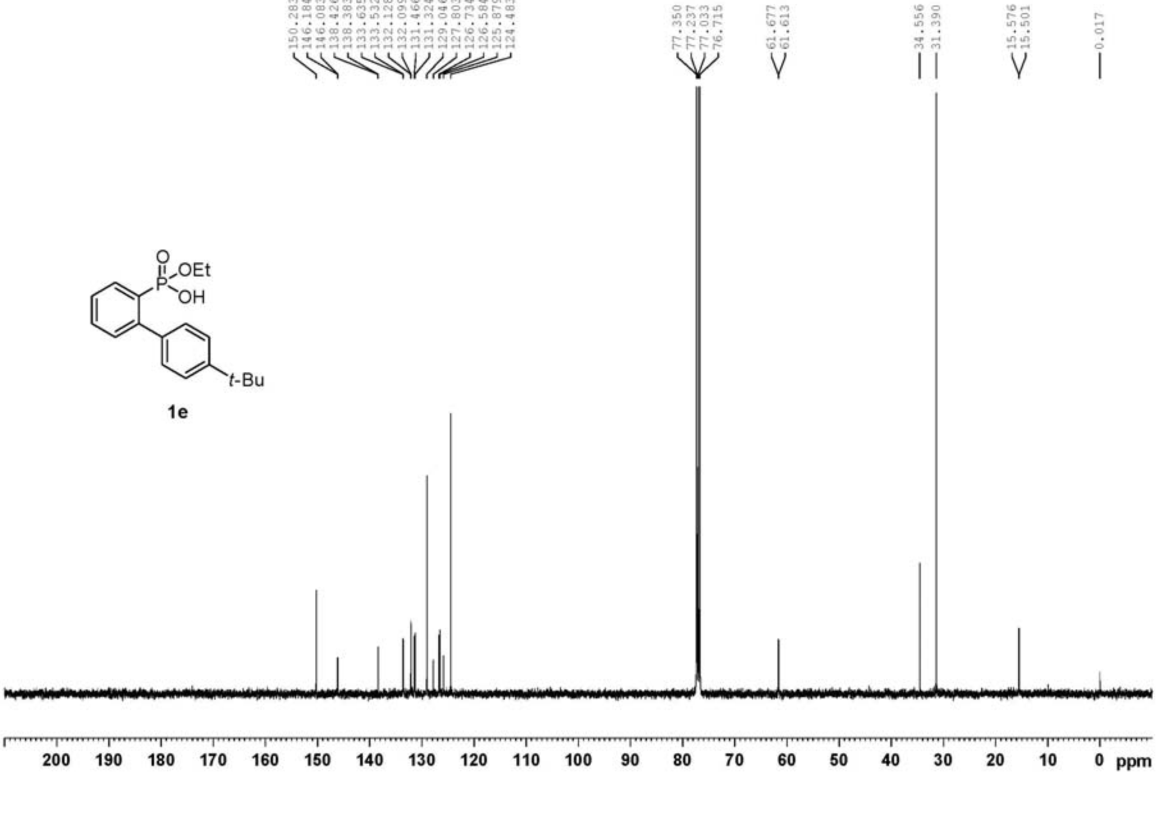
150.283
146.184
146.083
138.426
138.383
133.635
133.532
132.128
132.099
131.466
131.324
129.046
127.803
126.734
126.584
125.879
124.483
77.350
77.237
77.033
76.715
61.677
61.613
34.556
31.390
15.576
15.501
0.017
OEt
OH
t-Bu
1e
200
190
180
170
160
150
140
130
120
110
100
90
80
70
60
50
40
30
20
10
0
ppm

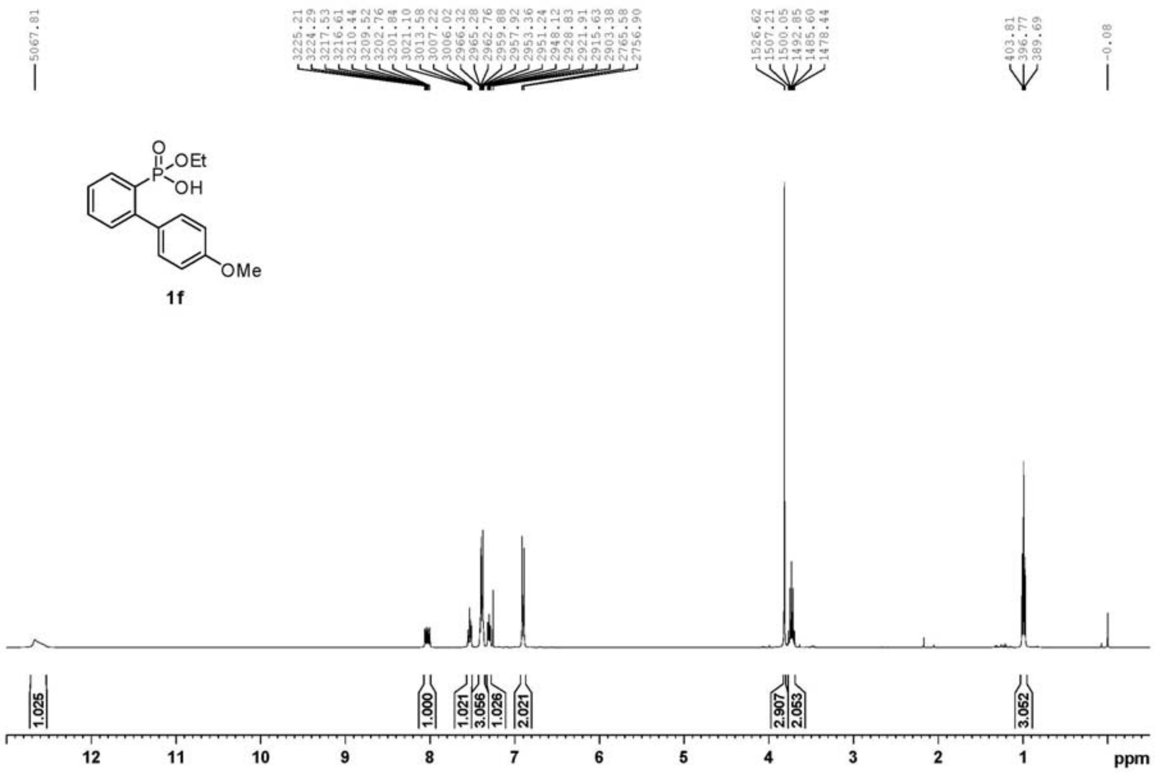

5067.81
3225.21
3224.29
3217.53
3216.61
3210.44
3209.52
3202.76
3201.84
3021.10
3013.58
3007.22
3006.02
2966.32
2965.28
2962.76
2959.88
2957.92
2953.36
2951.24
2948.12
2928.83
2921.91
2915.63
2903.38
2765.58
2756.90
1526.62
1507.21
1500.05
1492.85
1485.60
1478.44
403.81
396.77
389.69
-0.08
OEt
OH
OMe
1f
1.025
1.000
1.021
3.056
1.026
2.021
2.907
2.053
3.052
12 11 10 9 8 7 6 5 4 3 2 1 ppm

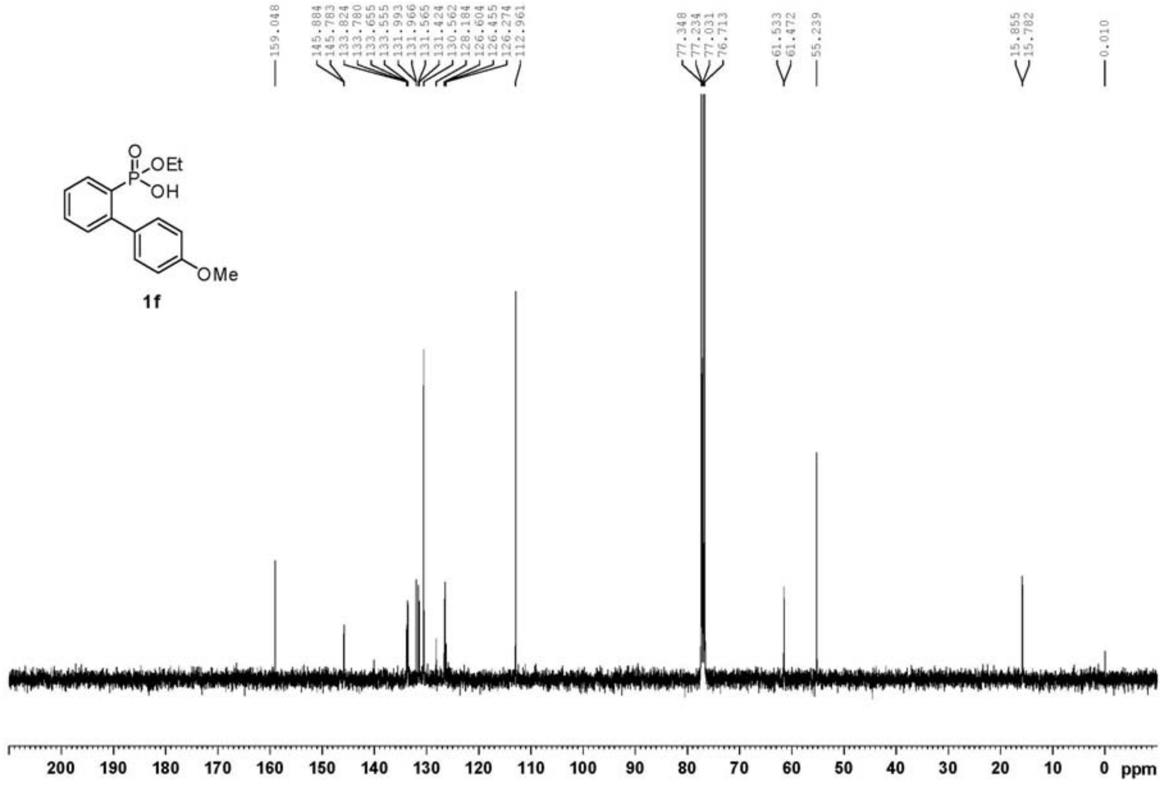

159.048
145.884
145.783
133.824
133.780
133.655
133.555
131.993
131.966
131.565
131.424
130.562
128.184
126.604
126.455
126.274
112.961
77.348
77.234
77.031
76.713
61.533
61.472
55.239
15.855
15.782
0.010
OEt
OH
OMe
1f
200 190 180 170 160 150 140 130 120 110 100 90 80 70 60 50 40 30 20 10 0 ppm

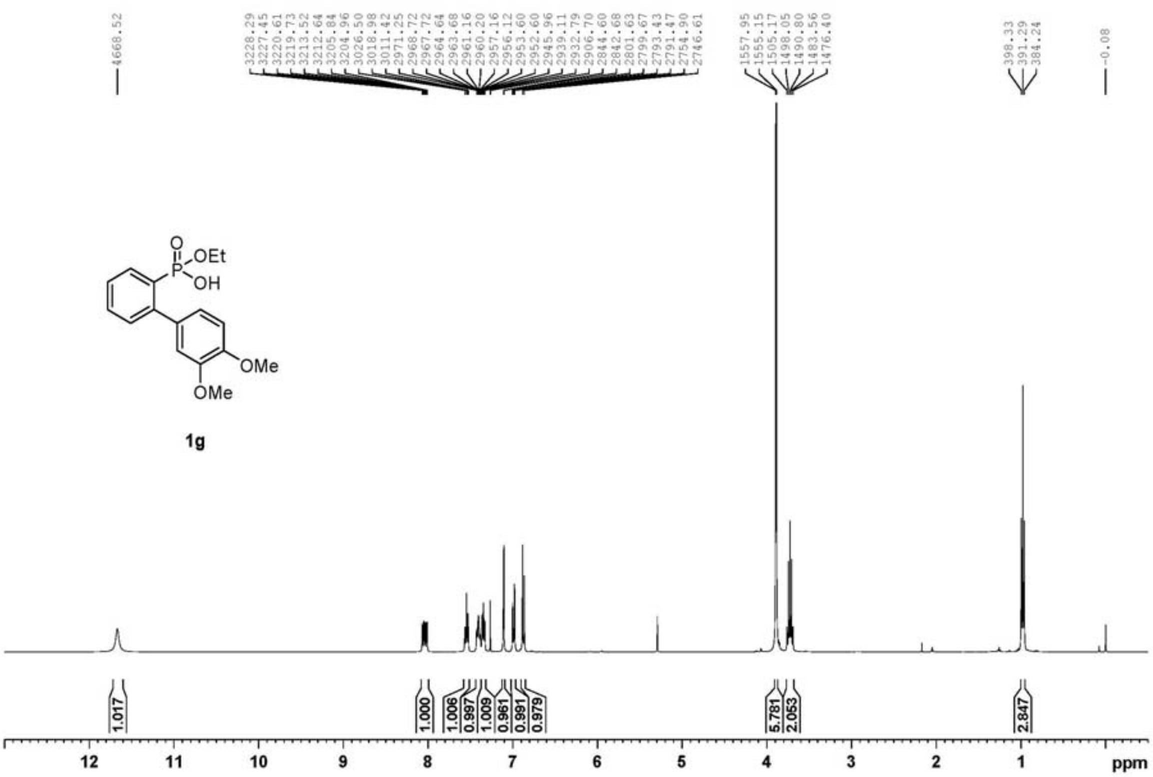

OEt
OH
OMe
OMe
1g
4668.52
3228.29
3227.45
3220.61
3219.73
3213.52
3212.64
3205.84
3204.96
3026.50
3018.98
3011.42
2971.25
2968.72
2967.72
2964.64
2963.68
2961.16
2960.20
2957.16
2956.12
2953.60
2952.60
2945.96
2939.11
2932.79
2906.70
2844.60
2842.68
2801.63
2799.67
2793.43
2791.47
2754.90
2746.61
1557.95
1555.15
1505.17
1498.05
1490.80
1483.56
1476.40
398.33
391.29
384.24
-0.08
1.017
1.000
1.006
0.997
1.009
0.961
0.991
0.979
5.781
2.053
2.847
ppm

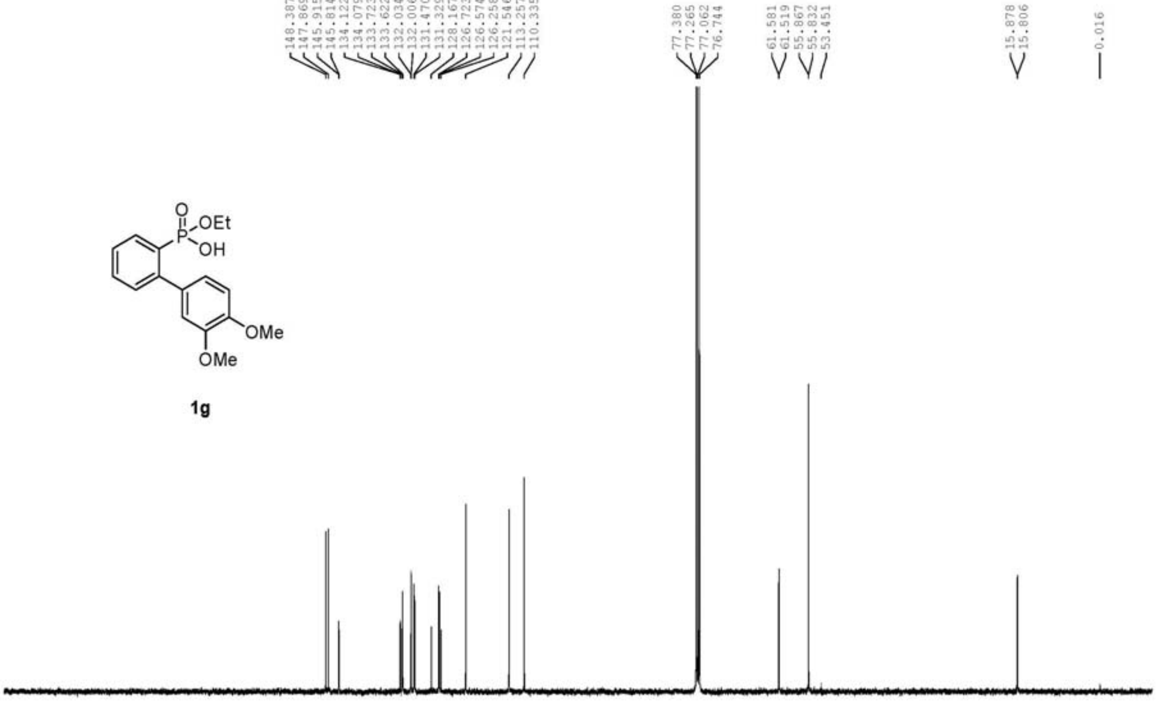

OEt
OH
OMe
OMe
1g
148.387
147.869
145.915
145.814
134.122
134.079
133.723
133.622
132.034
132.006
131.470
131.329
128.167
126.723
126.574
126.258
121.546
113.257
110.335
77.380
77.265
77.062
76.744
61.581
61.519
55.867
55.832
53.451
15.878
15.806
0.016
ppm

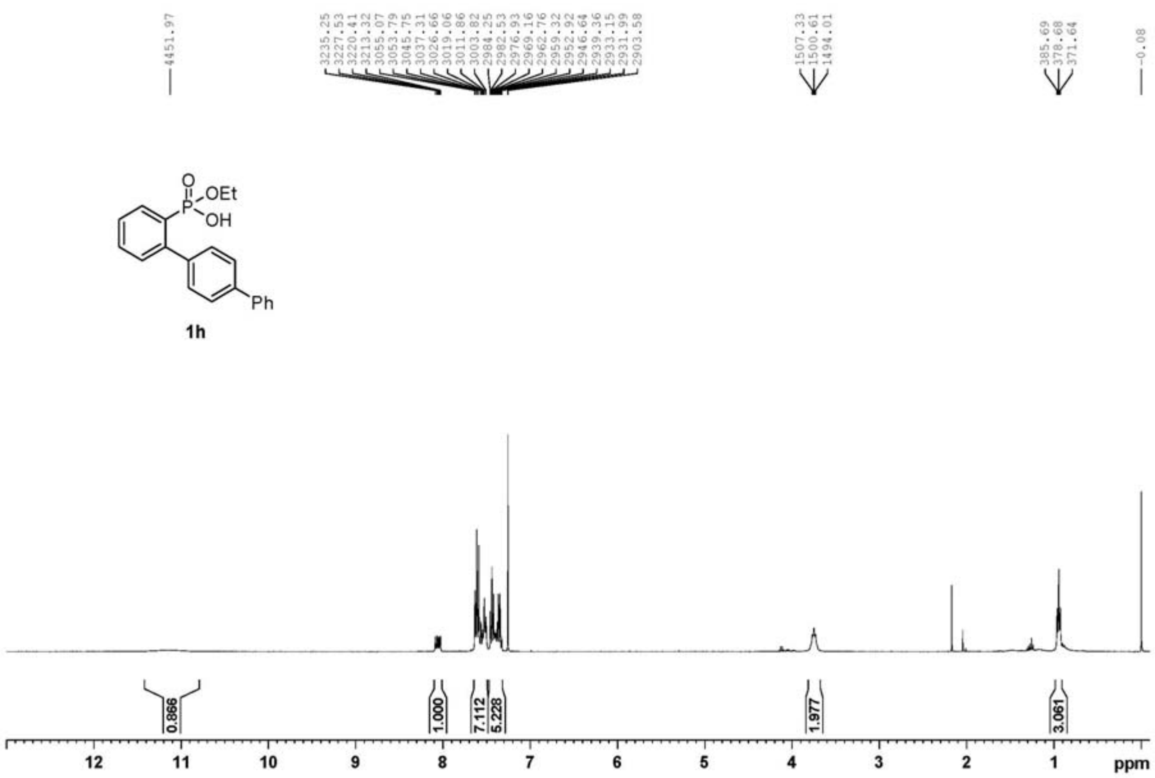
4451.97
3235.25 3227.53 3220.41 3213.32 3055.07 3053.79 3045.75 3037.31 3026.66 3019.06 3011.86 3003.82 2984.25 2982.53 2976.93 2969.16 2962.76 2959.32 2952.92 2946.64 2939.36 2933.15 2931.99 2903.58
1507.33 1500.61 1494.01
385.69 378.68 371.64
-0.08
OEt
OH
P
O
Ph
1h
0.866
1.000
7.112
5.228
1.977
3.061
12 11 10 9 8 7 6 5 4 3 2 1 ppm

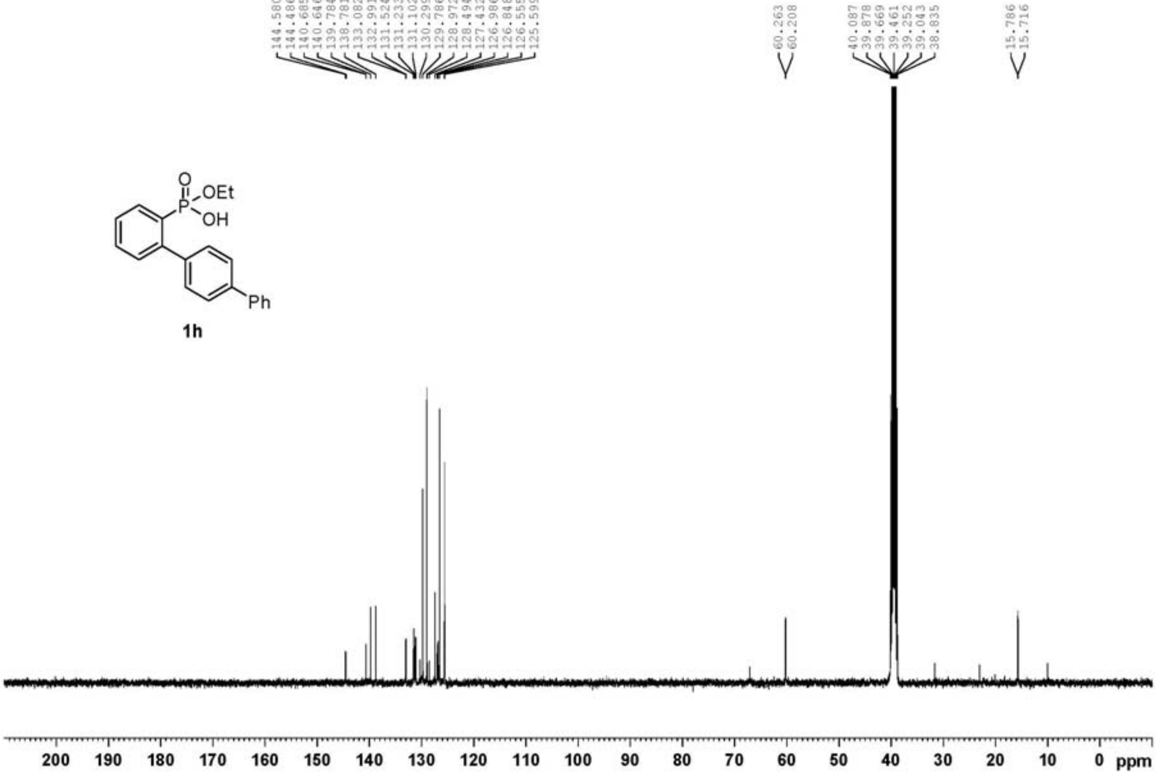
144.580 144.486 140.685 140.646 139.784 138.781 133.082 132.991 131.524 131.233 131.102 130.299 129.786 128.972 128.494 127.432 126.986 126.848 126.555 125.599
60.263 60.208
40.087 39.878 39.669 39.461 39.252 39.043 38.835
15.786 15.716
OEt
OH
P
O
Ph
1h
200 190 180 170 160 150 140 130 120 110 100 90 80 70 60 50 40 30 20 10 0 ppm

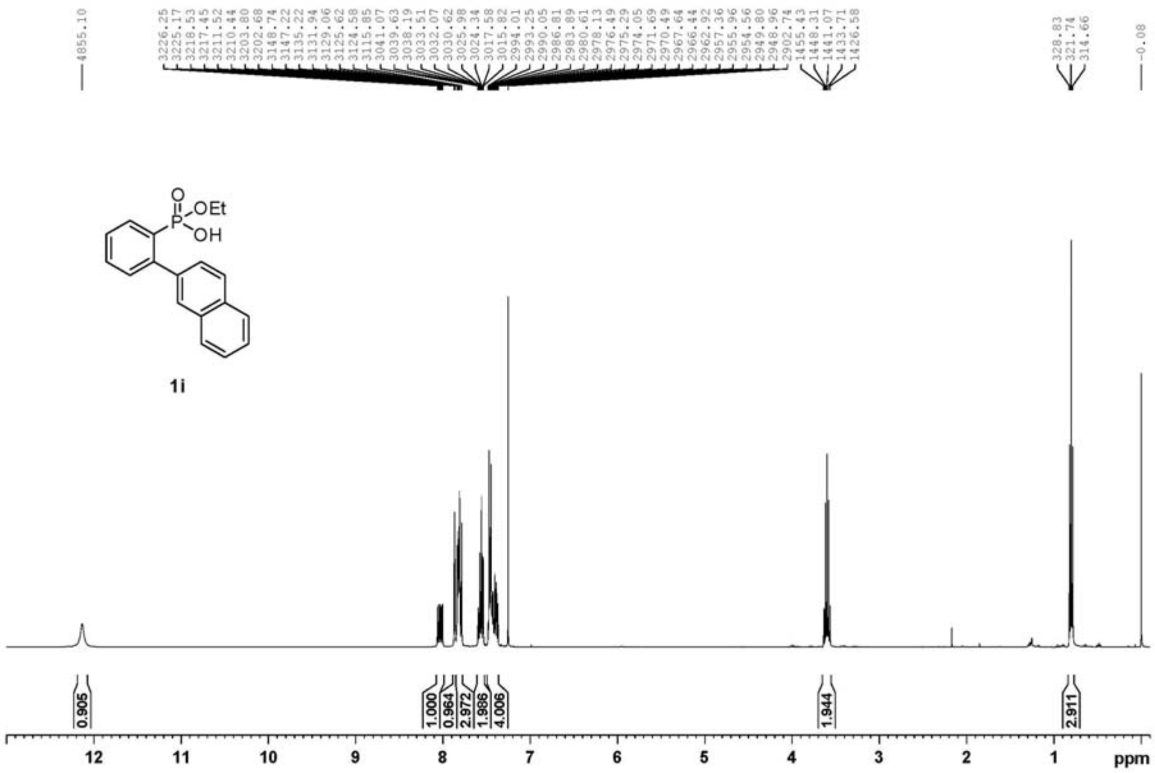
4855.10
3226.25 3225.17 3218.53 3217.45 3211.52 3210.44 3203.80 3202.68 3148.74 3147.22 3135.22 3131.94 3129.06 3125.62 3124.58 3115.85 3041.07 3039.63 3038.19 3033.51 3032.07 3030.62 3025.98 3024.34 3017.58 3015.82 2994.01 2993.25 2990.05 2986.81 2983.89 2980.61 2978.13 2976.49 2975.29 2974.05 2971.69 2970.49 2967.64 2966.44 2962.92 2957.36 2955.96 2954.56 2949.80 2948.96 2902.74
1455.43 1448.31 1441.07 1433.71 1426.58
328.83 321.74 314.66
-0.08
OEt
OH
1i
0.905
1.000
0.964
2.972
1.986
4.006
1.944
2.911
ppm

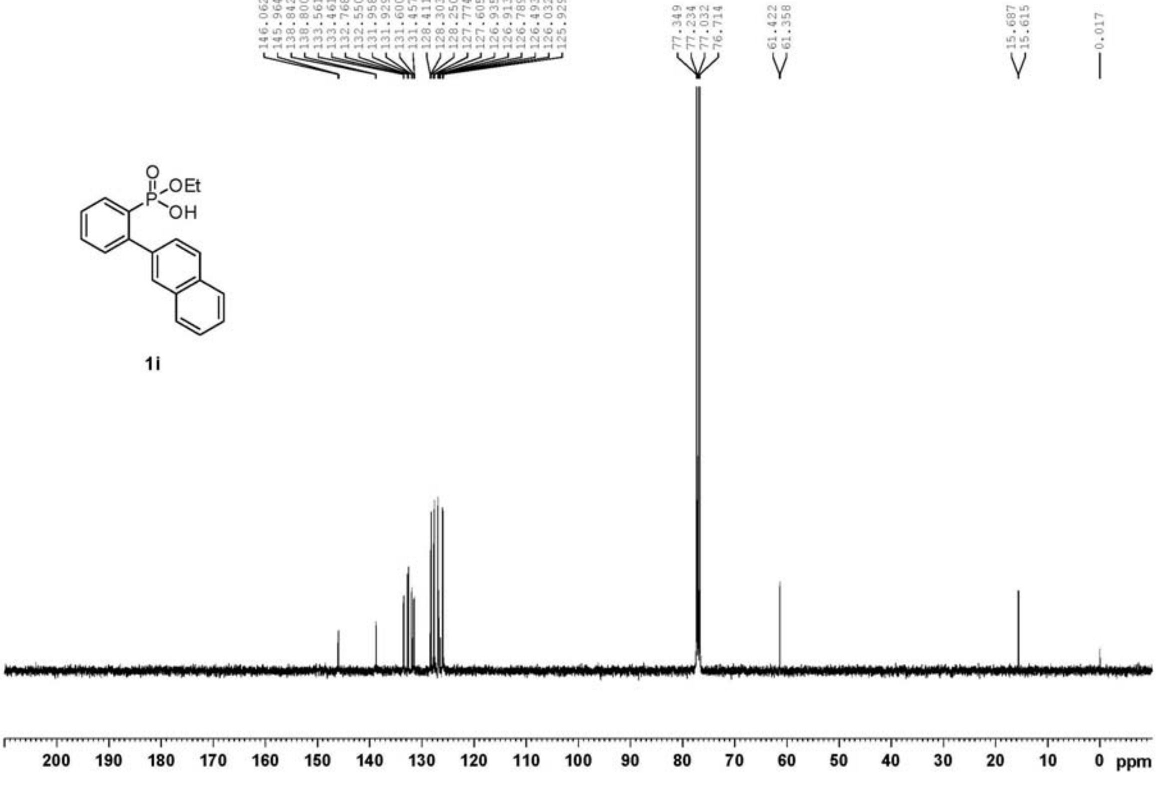
146.062 145.964 138.842 138.800 133.561 133.461 132.768 132.550 131.958 131.929 131.600 131.457 128.411 128.303 128.250 127.774 127.605 126.935 126.913 126.789 126.493 126.032 125.929
77.349 77.234 77.032 76.714
61.422 61.358
15.687 15.615
0.017
OEt
OH
1i
ppm

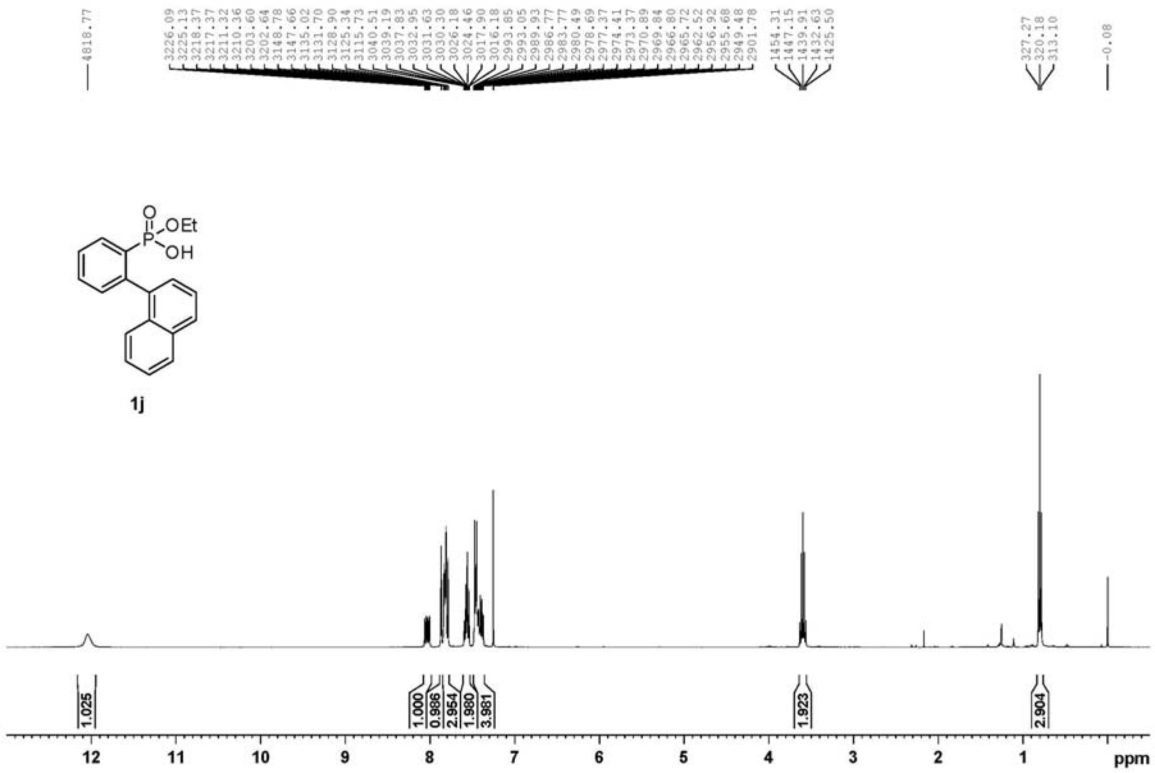
4818.77
3226.09
3225.13
3218.37
3217.37
3211.32
3210.36
3203.60
3202.64
3148.78
3147.66
3135.02
3131.70
3128.90
3125.34
3115.73
3040.51
3039.19
3037.83
3032.95
3031.63
3030.30
3026.18
3024.46
3017.90
3016.18
2993.85
2993.05
2989.93
2986.77
2983.77
2980.49
2978.69
2977.37
2974.41
2973.37
2970.89
2969.84
2966.80
2965.72
2962.52
2956.92
2955.68
2949.48
2901.78
1454.31
1447.15
1439.91
1432.63
1425.50
327.27
320.18
313.10
-0.08
OEt
OH
P
O
1j
1.025
1.000
0.986
2.954
1.980
3.981
1.923
2.904
12
11
10
9
8
7
6
5
4
3
2
1
0
ppm

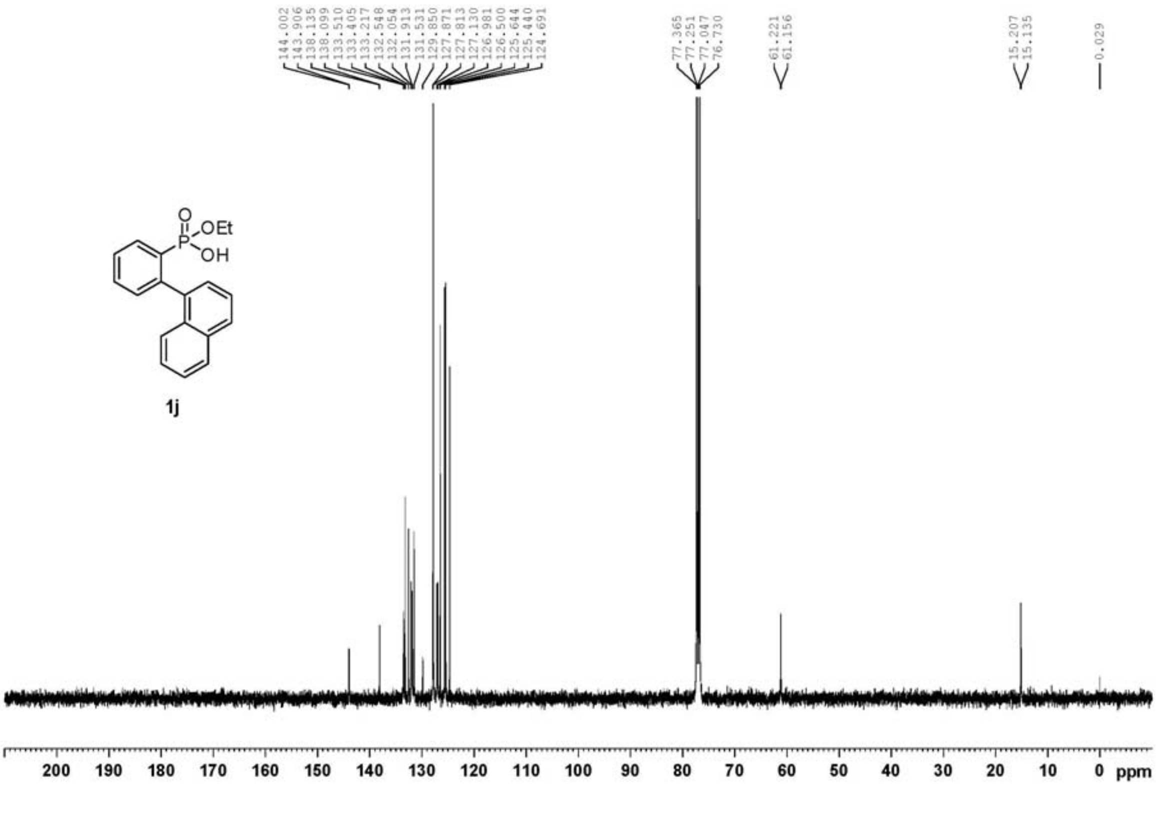
144.002
143.906
138.135
138.099
133.510
133.405
133.217
132.548
132.054
131.913
131.531
129.850
127.871
127.813
127.130
126.981
126.500
125.644
125.440
124.691
77.365
77.251
77.047
76.730
61.221
61.156
15.207
15.135
0.029
OEt
OH
P
O
1j
200
190
180
170
160
150
140
130
120
110
100
90
80
70
60
50
40
30
20
10
0
ppm

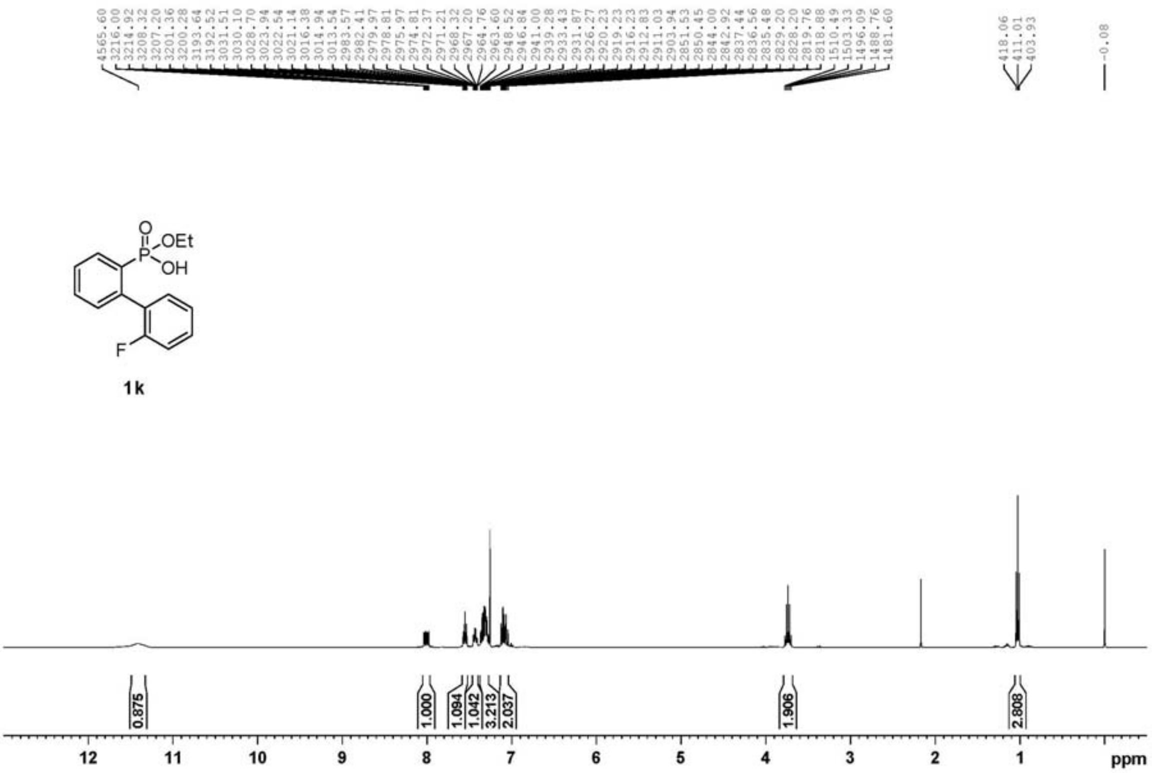
4565.60
3216.00
3214.92
3208.32
3207.20
3201.36
3200.28
3193.64
3192.52
3031.51
3030.10
3028.70
3023.94
3022.54
3021.14
3016.38
3014.94
3013.54
2983.57
2982.41
2979.97
2978.81
2975.97
2974.81
2972.37
2971.21
2968.32
2967.20
2964.76
2963.60
2948.52
2946.84
2941.00
2933.28
2933.43
2931.87
2926.27
2920.23
2919.23
2916.23
2912.83
2911.03
2903.94
2851.53
2850.45
2844.00
2842.92
2837.44
2836.56
2835.48
2829.20
2828.20
2819.76
2818.88
1510.49
1503.33
1496.09
1488.76
1481.60
418.06
411.01
403.93
-0.08
OEt
OH
F
1k
0.875
1.000
1.094
1.042
3.213
2.037
1.906
2.808
12
11
10
9
8
7
6
5
4
3
2
1
ppm

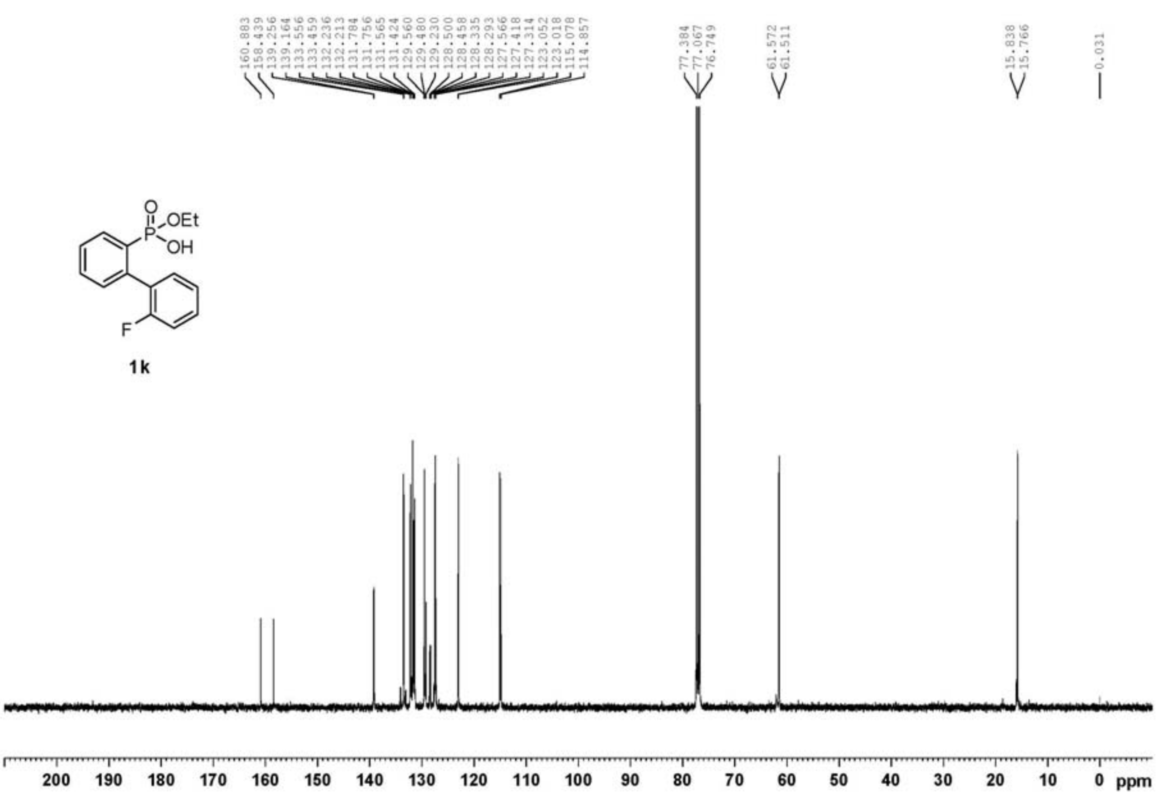
160.883
158.439
139.256
139.164
133.556
133.459
132.236
132.213
131.784
131.756
131.565
131.424
129.560
129.480
129.230
128.500
128.458
128.335
128.293
127.566
127.418
127.314
123.052
123.018
115.078
114.857
77.384
77.067
76.749
61.572
61.511
15.838
15.766
0.031
OEt
OH
F
1k
200
190
180
170
160
150
140
130
120
110
100
90
80
70
60
50
40
30
20
10
0
ppm

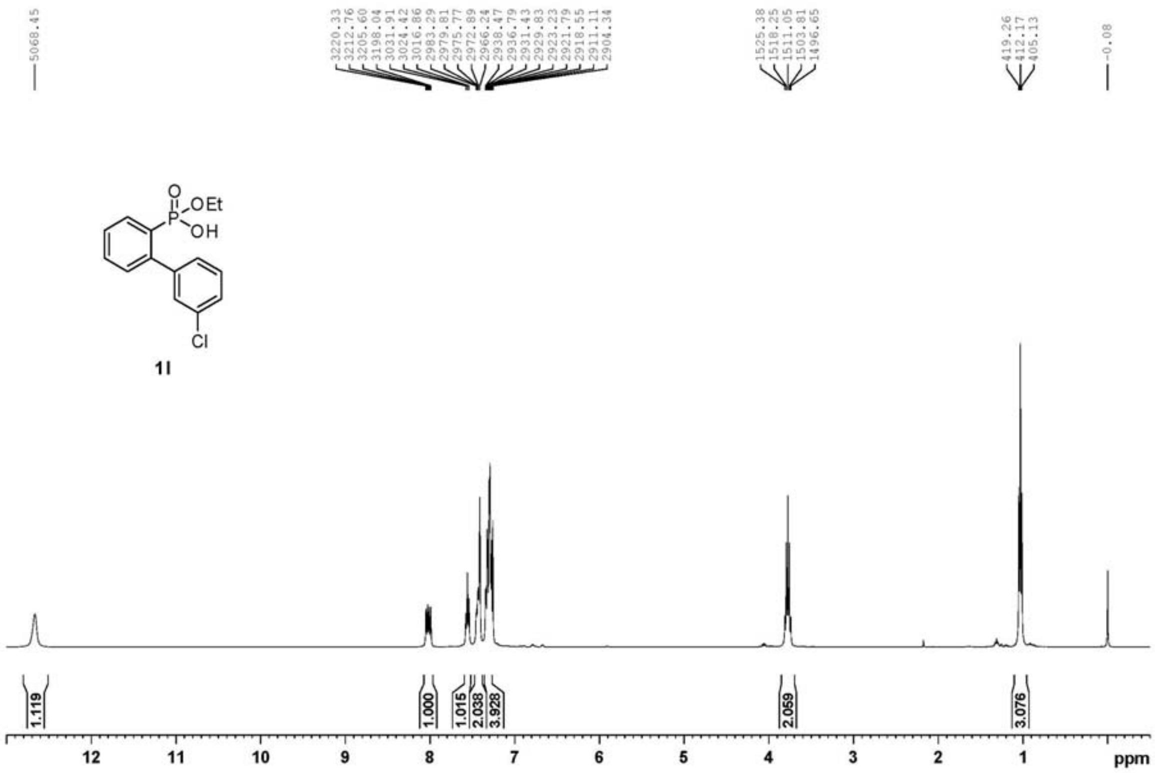
5068.45
3220.33 3212.76 3205.60 3198.04 3031.91 3024.42 3016.86 2983.29 2979.81 2975.77 2972.89 2966.24 2938.47 2936.79 2931.43 2929.83 2923.23 2921.79 2918.55 2911.11 2904.34
1525.38 1518.25 1511.05 1503.81 1496.65
419.26 412.17 405.13
-0.08
OEt
OH
P
O
Cl
1l
1.119
1.000
1.015
2.038
3.928
2.059
3.076
12 11 10 9 8 7 6 5 4 3 2 1 ppm

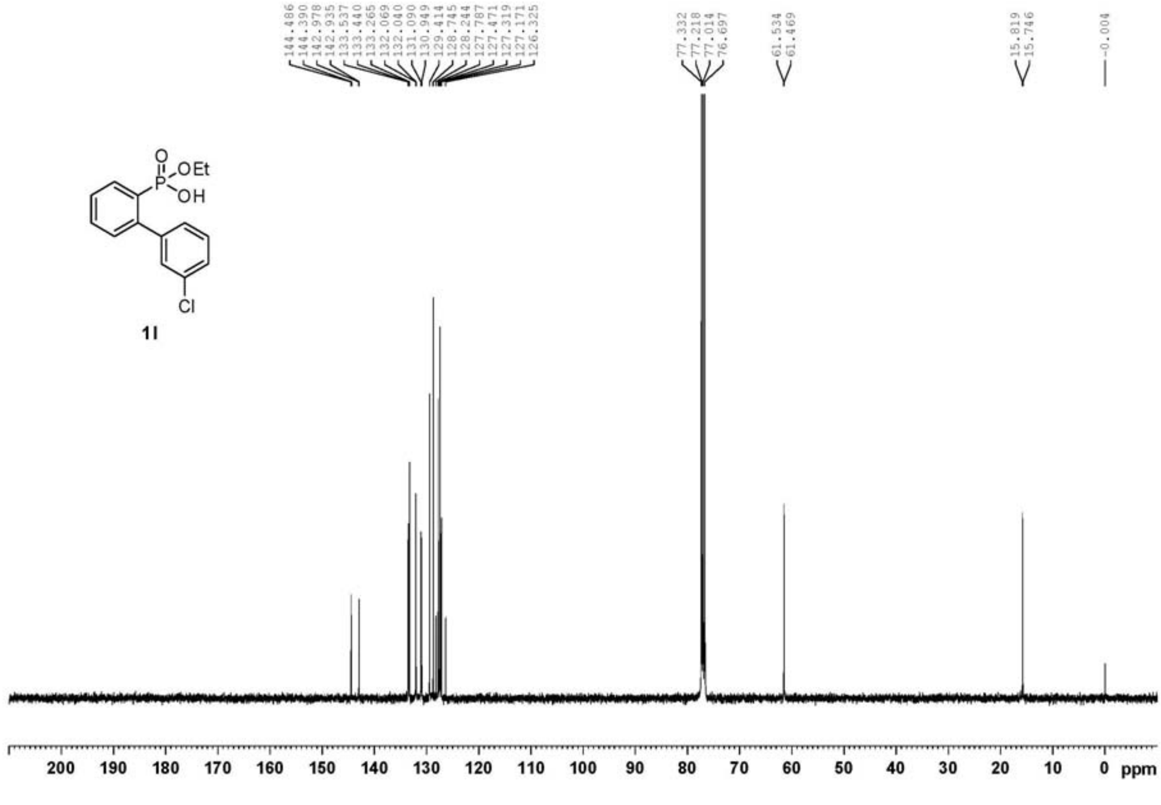
144.486 144.390 142.978 142.935 133.537 133.440 133.265 132.069 132.040 131.090 130.949 129.414 128.745 128.244 127.787 127.471 127.319 127.171 126.325
77.332 77.218 77.014 76.697
61.534 61.469
15.819 15.746
-0.004
OEt
OH
P
O
Cl
1l
200 190 180 170 160 150 140 130 120 110 100 90 80 70 60 50 40 30 20 10 0 ppm

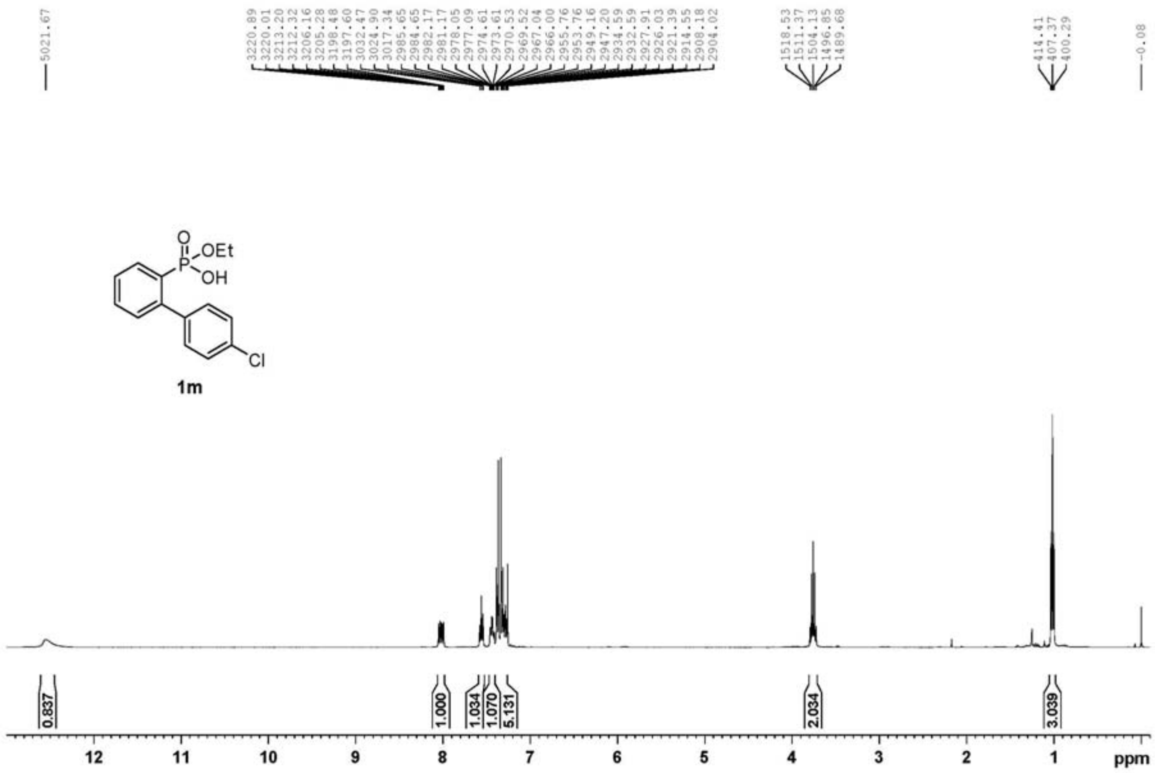
OEt
OH
Cl
1m
ppm

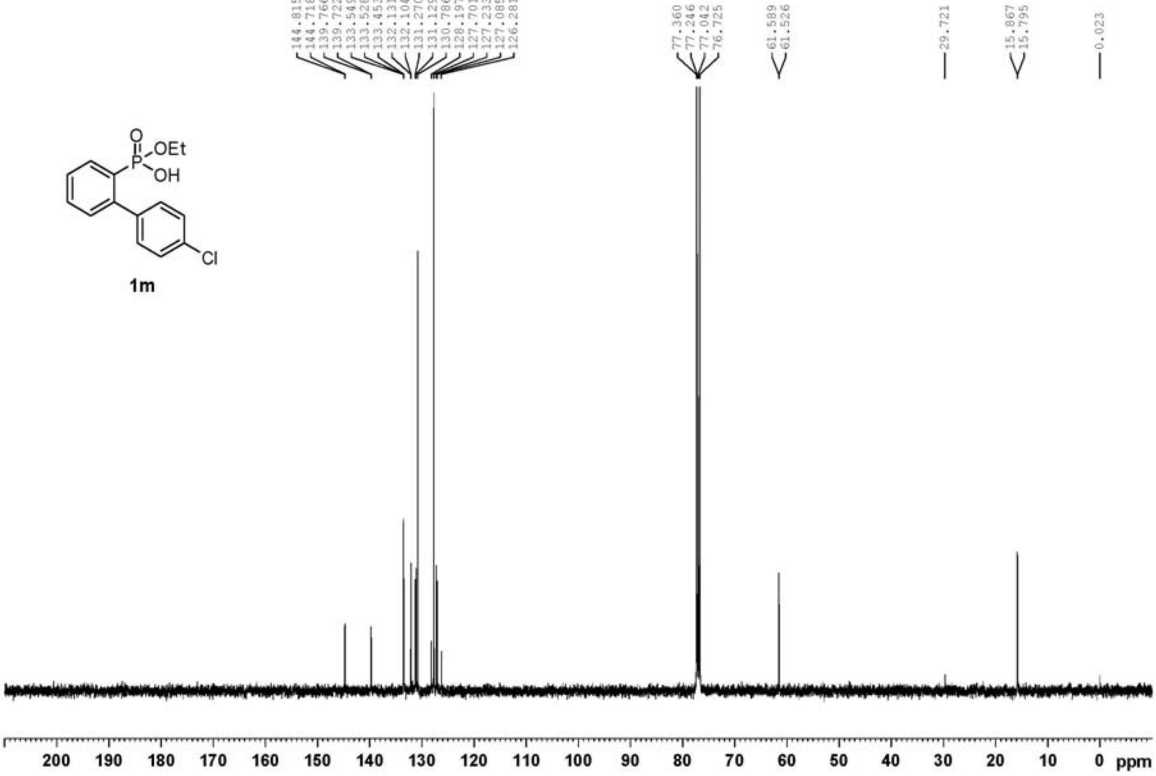
144.815
144.718
139.766
139.722
133.549
133.528
133.453
132.131
132.104
131.270
131.129
130.786
128.197
127.701
127.233
127.085
126.281
77.360
77.246
77.042
76.725
61.589
61.526
29.721
15.867
15.795
0.023
OEt
OH
Cl
1m
ppm

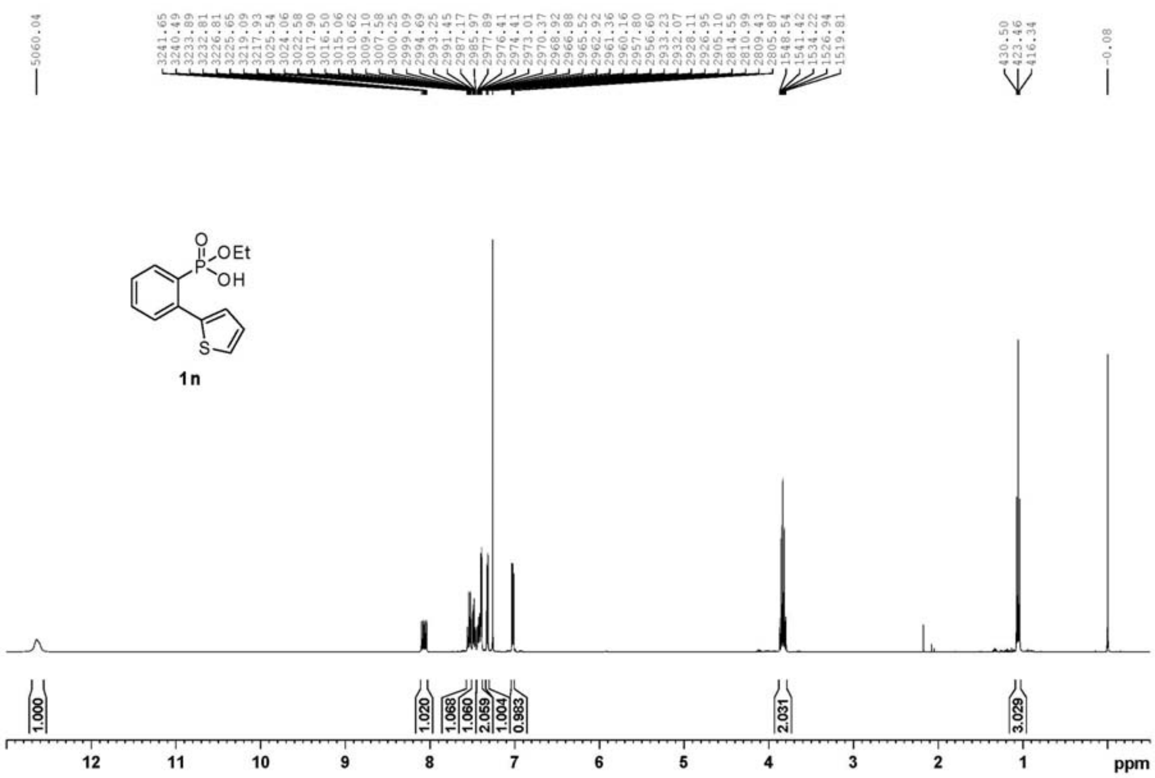
5060.04
3241.65
3240.49
3233.89
3232.81
3226.81
3225.65
3219.09
3217.93
3025.54
3024.06
3022.58
3017.90
3016.50
3015.06
3010.62
3009.10
3007.58
3000.25
2999.09
2994.69
2993.25
2991.45
2987.17
2985.97
2977.89
2976.41
2974.41
2973.01
2970.37
2968.92
2966.88
2965.52
2962.92
2961.36
2960.16
2957.80
2956.60
2933.23
2932.07
2928.11
2926.95
2905.10
2814.55
2810.99
2809.43
2805.87
1548.54
1541.42
1534.22
1526.94
1519.81
430.50
423.46
416.34
-0.08
OEt
OH
S
1n
1.000
1.020
1.068
1.060
2.059
1.004
0.983
2.031
3.029
12
11
10
9
8
7
6
5
4
3
2
1
ppm

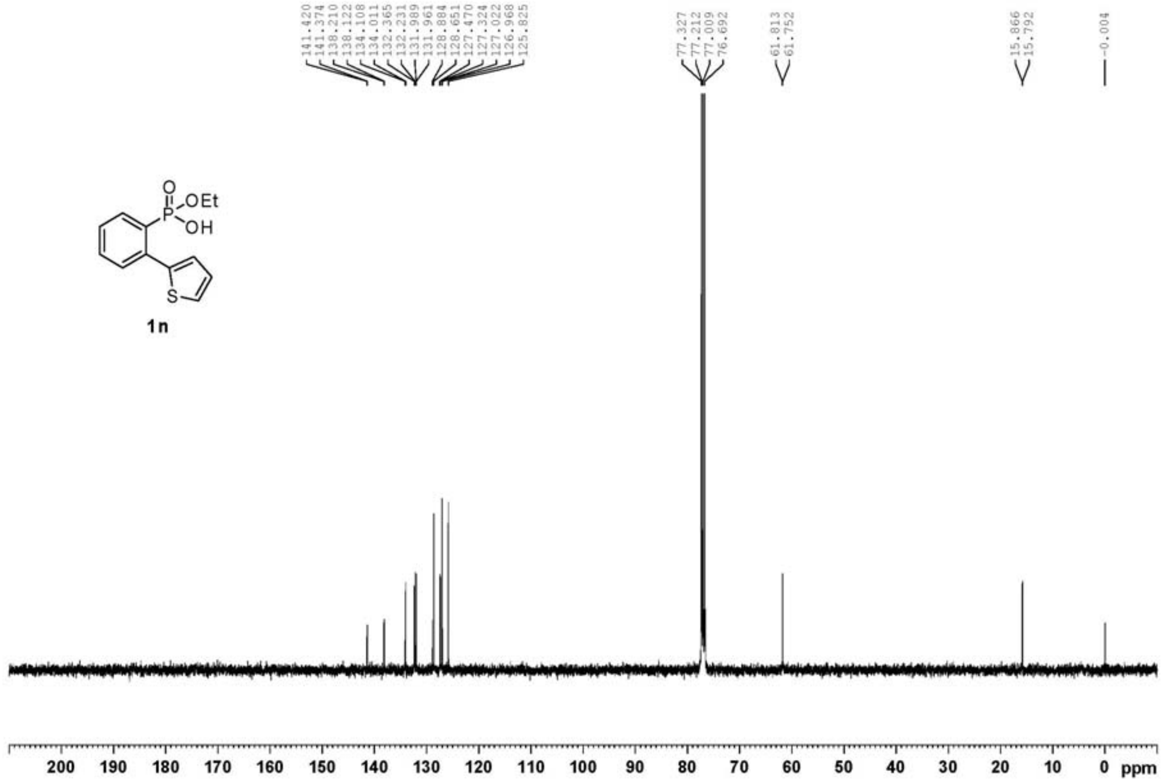
141.420
141.374
138.210
138.122
134.108
134.011
132.365
132.231
131.989
131.961
128.884
128.651
127.470
127.324
127.022
126.968
125.825
77.327
77.212
77.009
76.692
61.813
61.752
15.866
15.792
-0.004
OEt
OH
S
1n
200
190
180
170
160
150
140
130
120
110
100
90
80
70
60
50
40
30
20
10
0
ppm

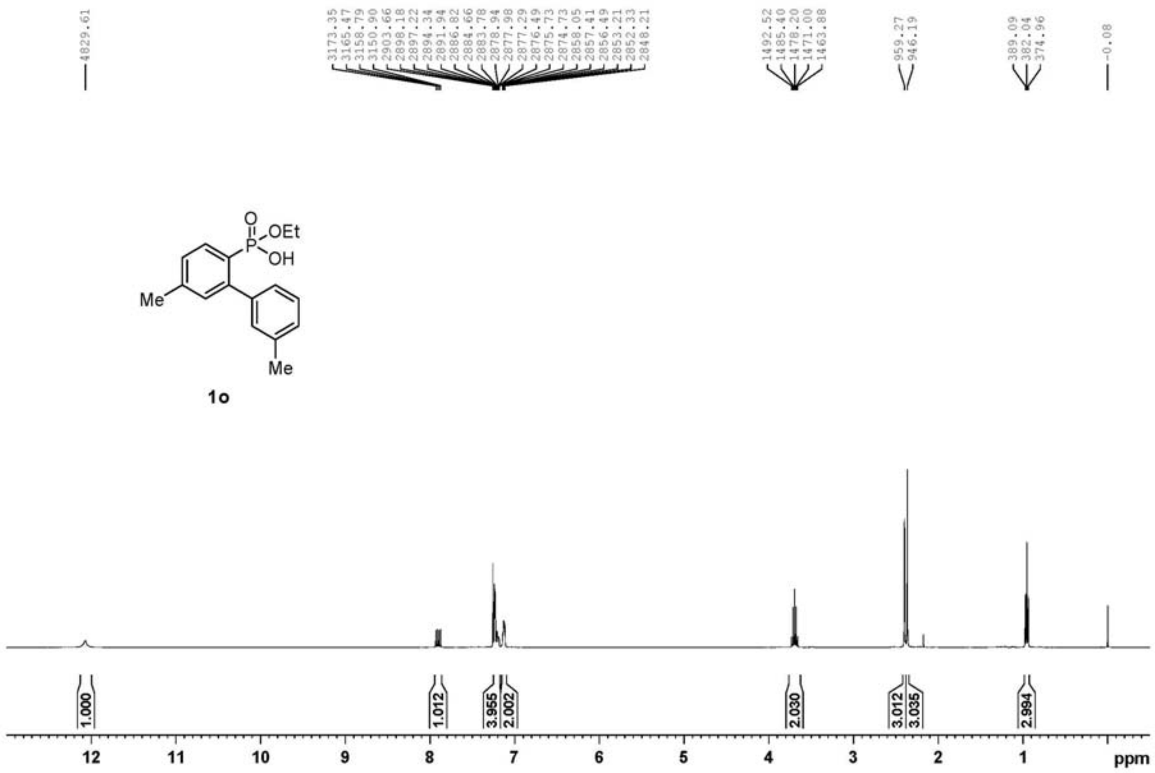
4829.61
3173.35
3165.47
3158.79
3150.90
2903.66
2898.18
2897.22
2894.34
2891.94
2886.82
2884.66
2883.78
2878.94
2877.98
2877.29
2876.49
2875.73
2874.73
2858.05
2857.41
2856.49
2853.21
2852.33
2848.21
1492.52
1485.40
1478.20
1471.00
1463.88
959.27
946.19
389.09
382.04
374.96
-0.08
O
P
OEt
OH
Me
Me
1o
1.000
1.012
3.955
2.002
2.030
3.012
3.035
2.994
12 11 10 9 8 7 6 5 4 3 2 1 ppm

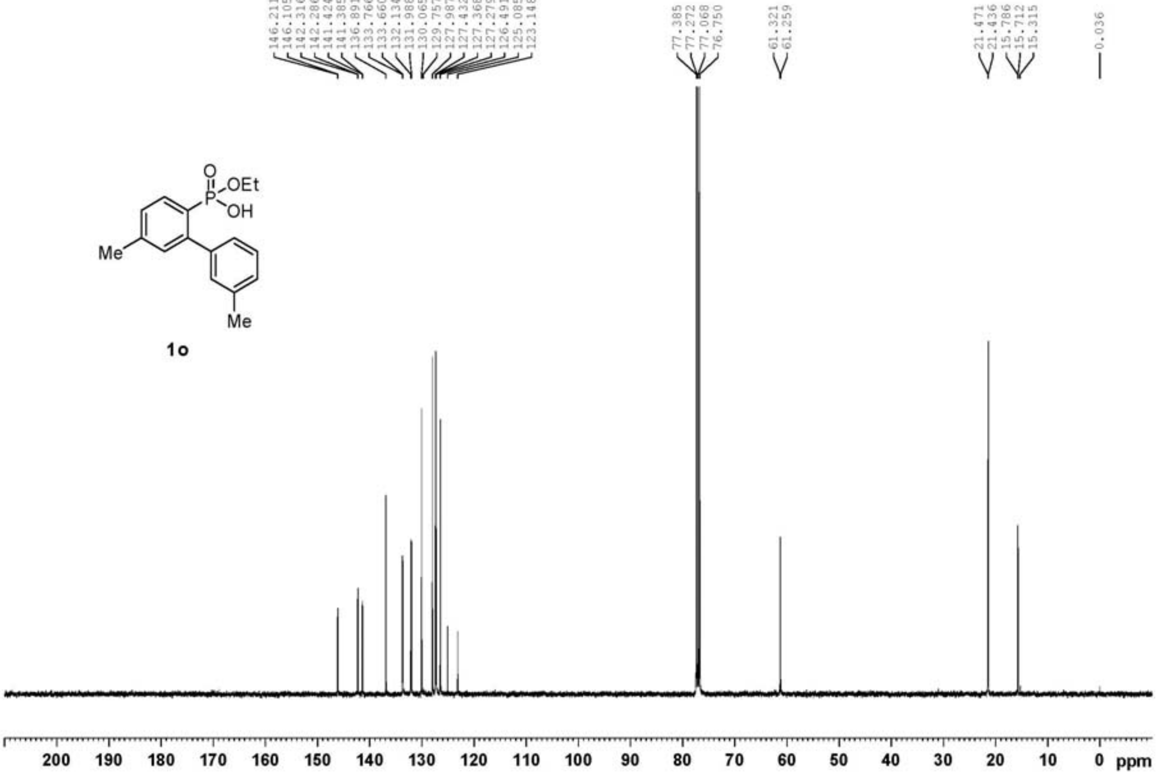
146.211
146.105
142.316
142.286
141.424
141.385
136.891
133.766
133.660
132.134
131.988
130.065
129.757
127.987
127.432
127.368
127.279
126.491
125.085
123.148
77.385
77.272
77.068
76.750
61.321
61.259
21.471
21.436
15.786
15.712
15.315
0.036
O
P
OEt
OH
Me
Me
1o
200 190 180 170 160 150 140 130 120 110 100 90 80 70 60 50 40 30 20 10 0 ppm

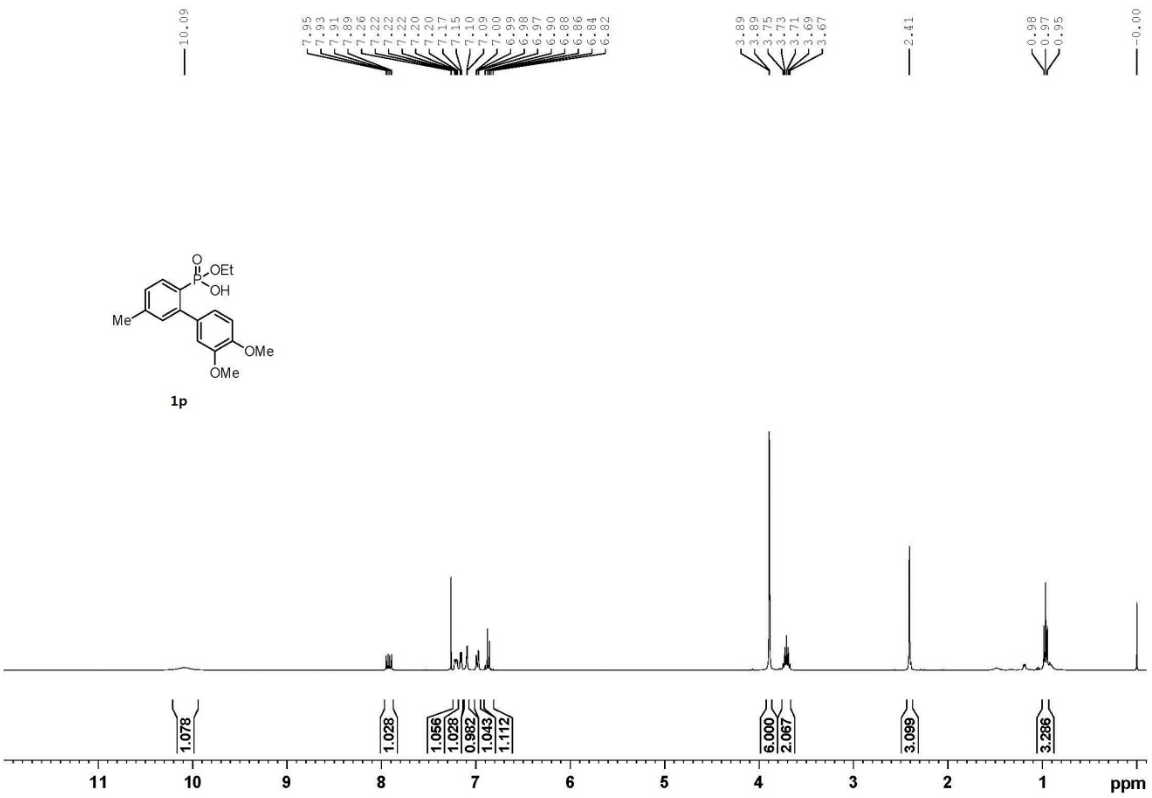
10.09
7.95 7.93 7.91 7.89 7.26 7.22 7.22 7.22 7.20 7.20 7.17 7.15 7.10 7.09 7.00 6.99 6.98 6.97 6.90 6.88 6.86 6.84 6.82
3.89 3.89 3.75 3.73 3.71 3.69 3.67
2.41
0.98 0.97 0.95
-0.00
O
P
OEt
OH
Me
OMe
OMe
1p
1.078
1.028
1.056 1.028 0.982 1.043 1.112
6.000 2.067
3.099
3.286
11 10 9 8 7 6 5 4 3 2 1 ppm

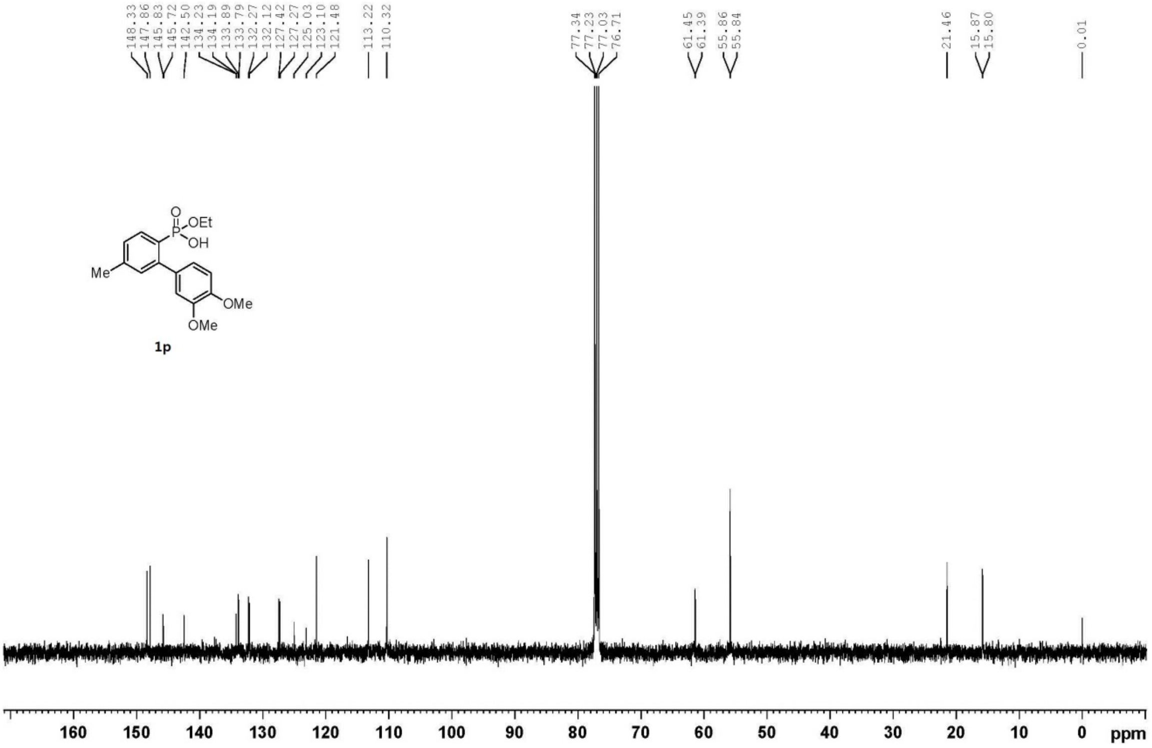
148.33 147.86 145.83 145.72 142.50 134.23 134.19 133.89 133.79 132.27 132.12 127.42 127.27 125.03 123.10 121.48
113.22 110.32
77.34 77.23 77.03 76.71
61.45 61.39 55.86 55.84
21.46 15.87 15.80
0.01
O
P
OEt
OH
Me
OMe
OMe
1p
160 150 140 130 120 110 100 90 80 70 60 50 40 30 20 10 0 ppm

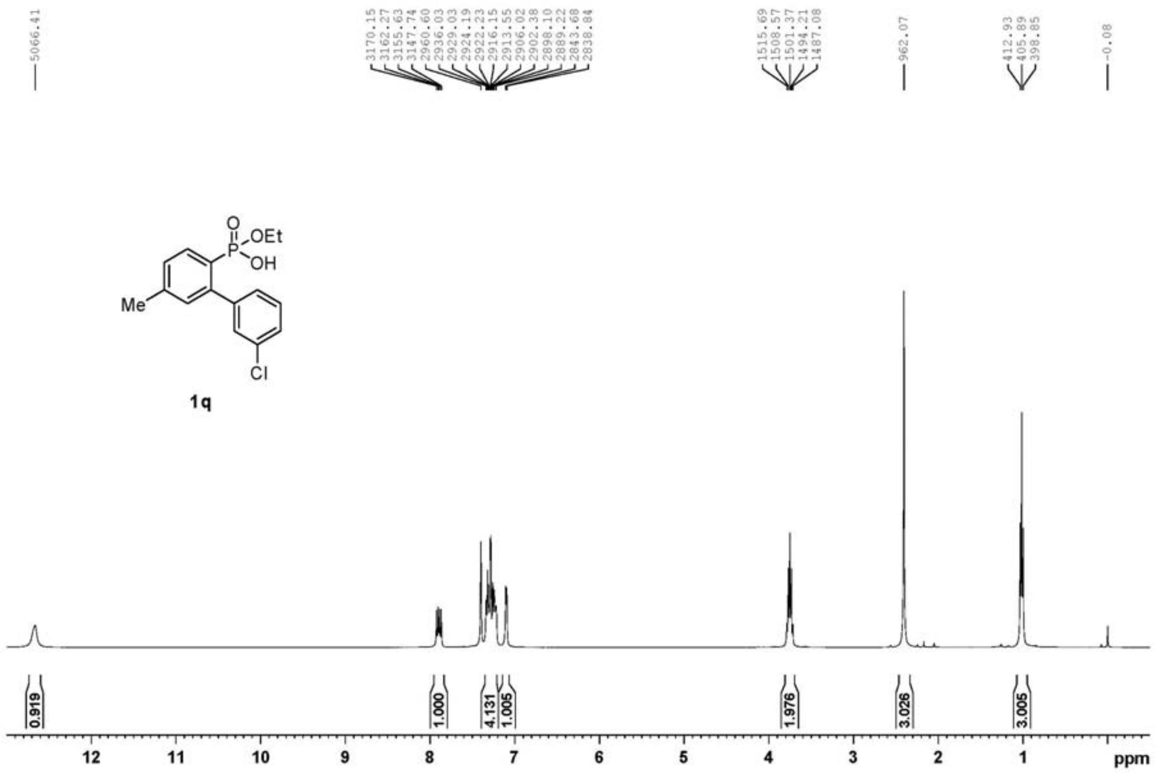
5066.41
3170.15
3162.27
3155.63
3147.74
2960.60
2936.03
2929.03
2924.19
2922.23
2916.15
2913.55
2906.02
2902.38
2898.10
2889.22
2843.68
2838.84
1515.69
1508.57
1501.37
1494.21
1487.08
962.07
412.93
405.89
398.85
-0.08
OEt
OH
Me
Cl
1q
0.919
1.000
4.131
1.005
1.976
3.026
3.005
12 11 10 9 8 7 6 5 4 3 2 1 ppm

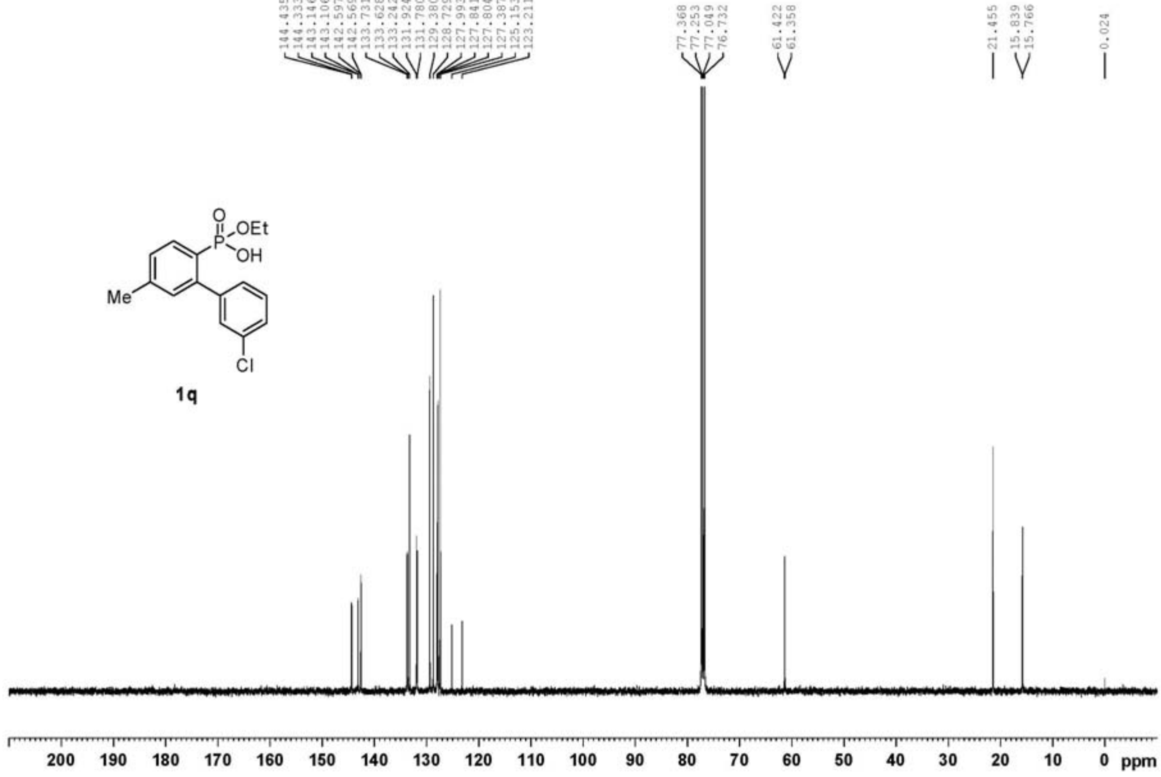
144.435
144.333
143.146
143.106
142.597
142.569
133.731
133.628
133.242
131.924
131.780
129.380
128.729
127.993
127.841
127.804
127.387
125.153
123.211
77.368
77.253
77.049
76.732
61.422
61.358
21.455
15.839
15.766
0.024
OEt
OH
Me
Cl
1q
200 190 180 170 160 150 140 130 120 110 100 90 80 70 60 50 40 30 20 10 0 ppm

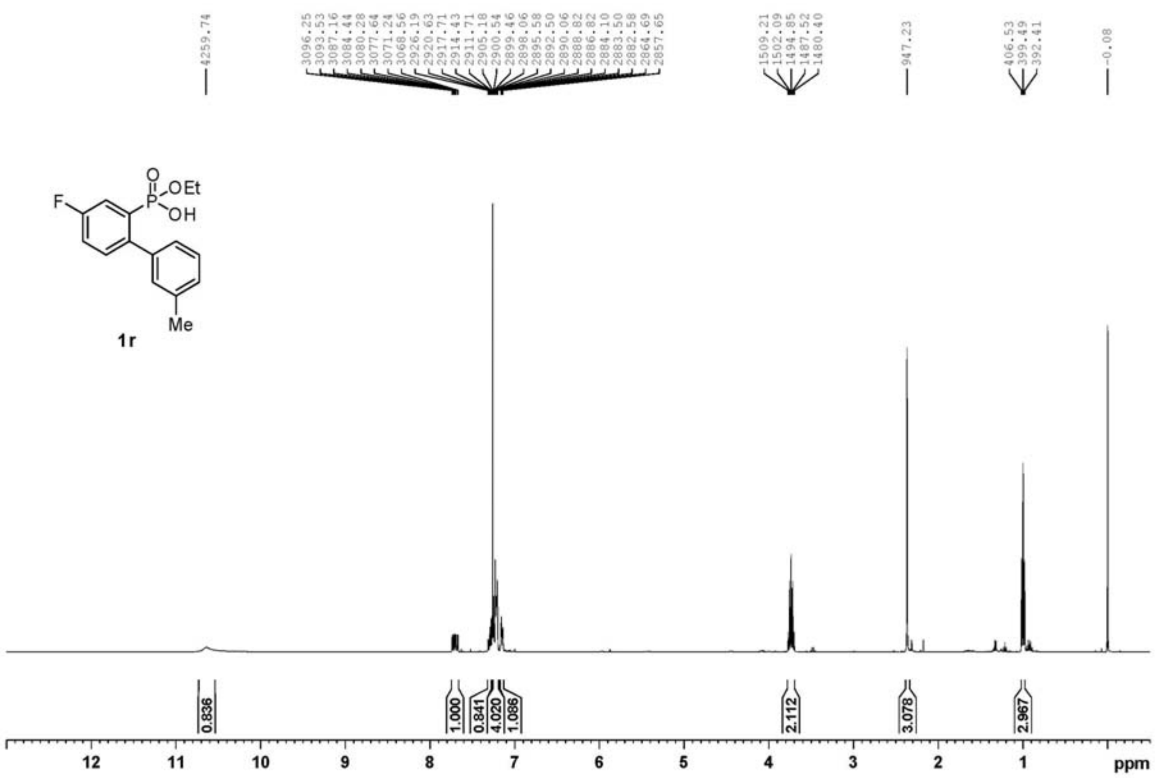
F
O
P
OEt
OH
Me
1r
ppm

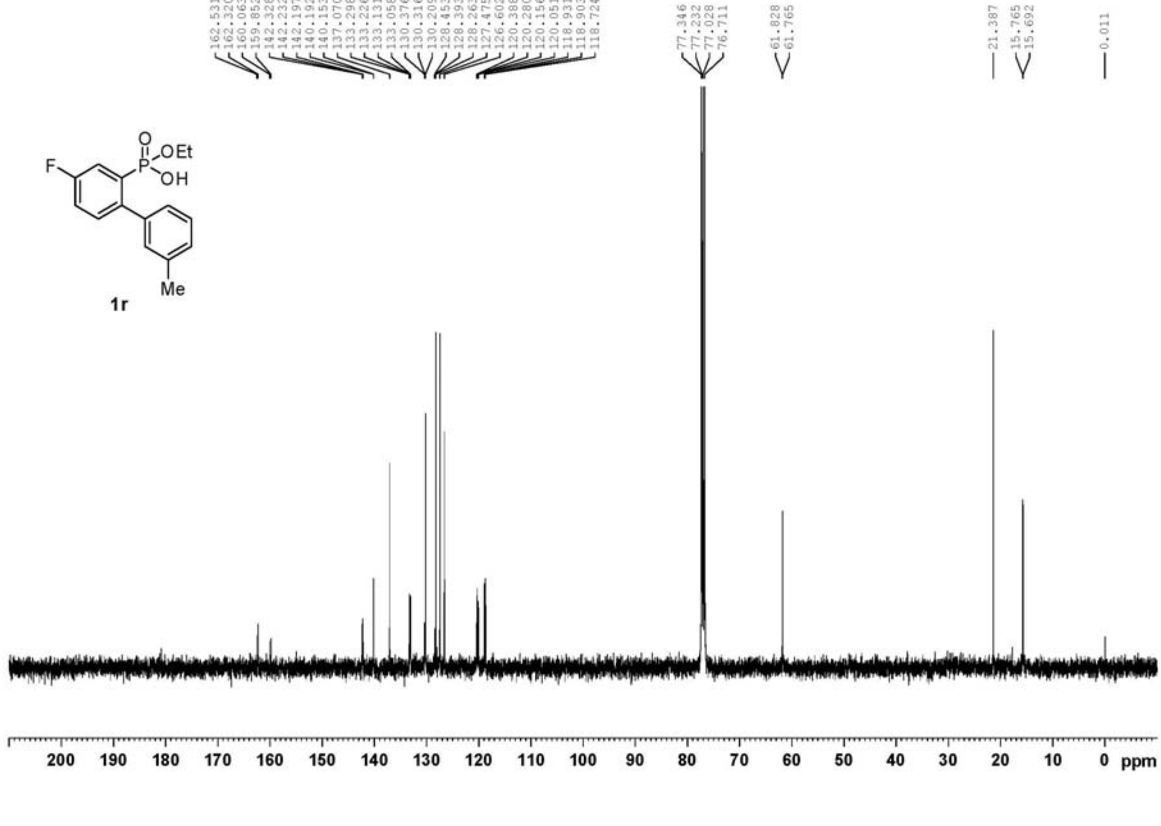
F
O
P
OEt
OH
Me
1r
ppm

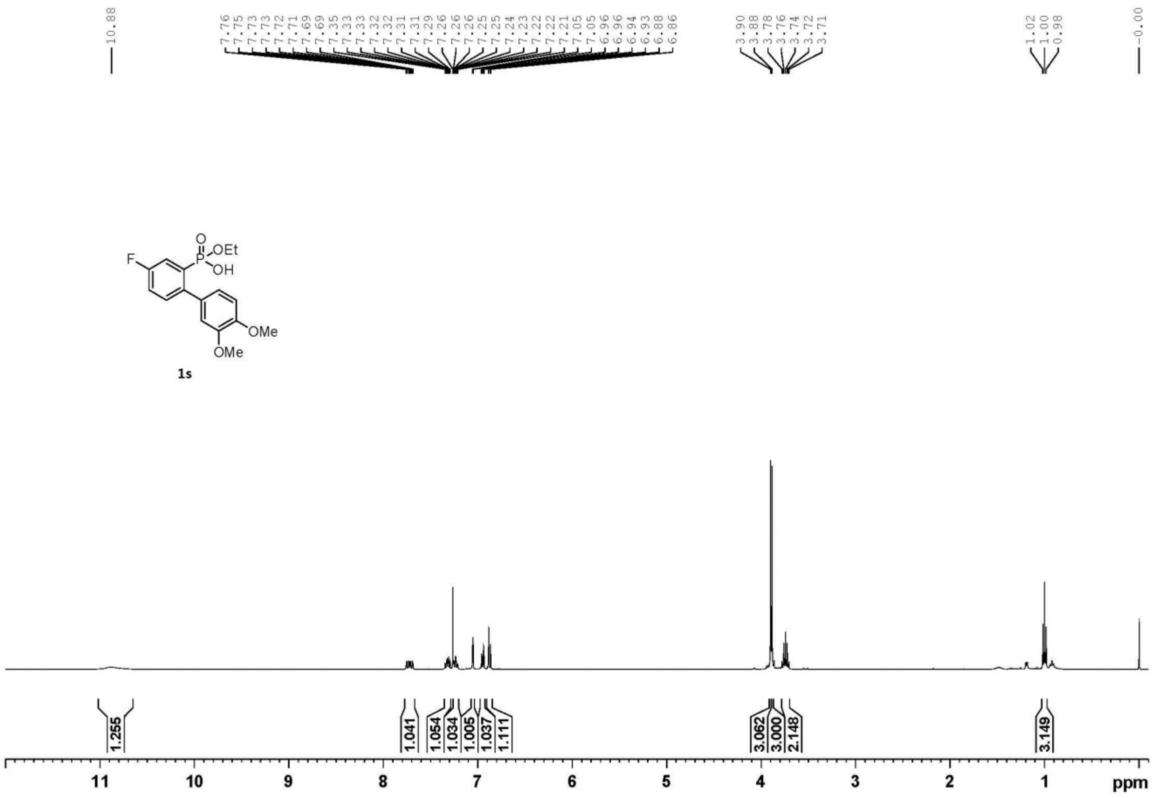
10.88
7.76 7.75 7.73 7.73 7.72 7.71 7.69 7.69 7.35 7.33 7.33 7.32 7.32 7.31 7.31 7.29 7.26 7.26 7.26 7.25 7.25 7.24 7.23 7.22 7.22 7.21 7.05 7.05 6.96 6.94 6.93 6.88 6.86
3.90 3.88 3.78 3.76 3.74 3.72 3.71
1.02 1.00 0.98
-0.00
F
O
P
OEt
OH
OMe
OMe
1s
1.255
1.041
1.054
1.034
1.005
1.037
1.111
3.062
3.000
2.148
3.149
11 10 9 8 7 6 5 4 3 2 1 ppm

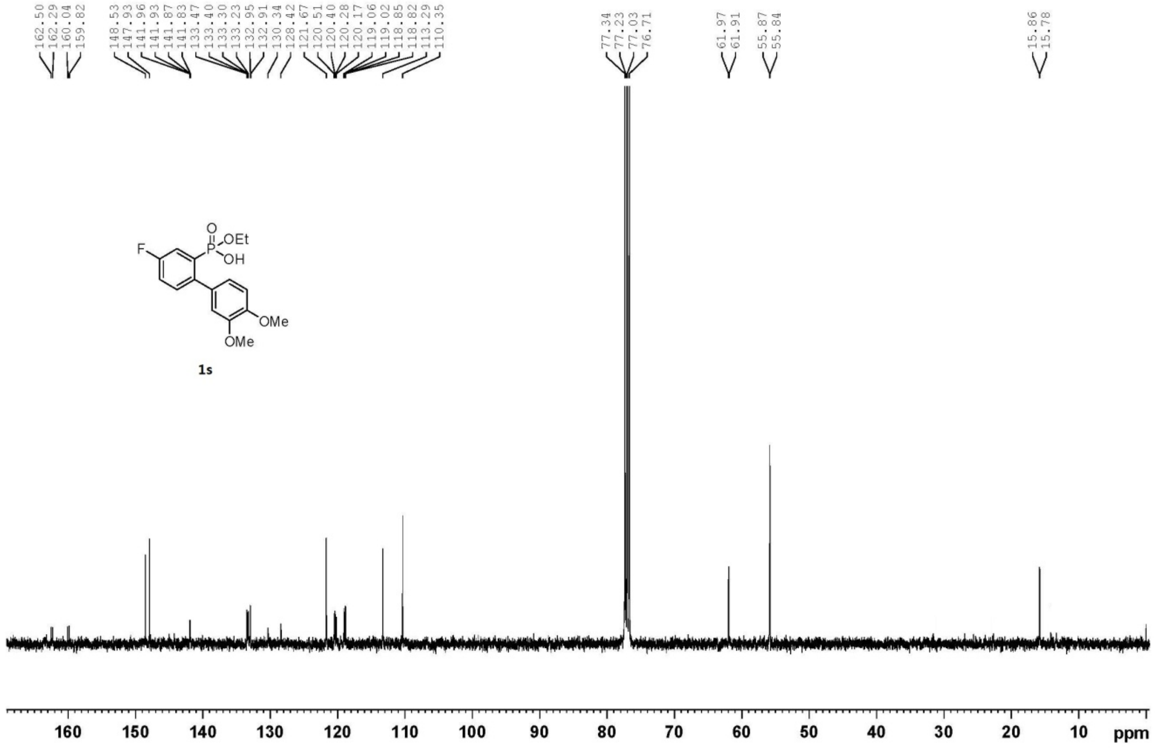
162.50 162.29 160.04 159.82
148.53 147.93 141.96 141.93 141.87 141.83 133.47 133.40 133.30 133.23 132.95 132.91 130.34 128.42 121.67 120.51 120.40 120.28 120.17 119.06 119.02 118.85 118.82 113.29 110.35
77.34 77.23 77.03 76.71
61.97 61.91
55.87 55.84
15.86 15.78
F
O
P
OEt
OH
OMe
OMe
1s
160 150 140 130 120 110 100 90 80 70 60 50 40 30 20 10 ppm

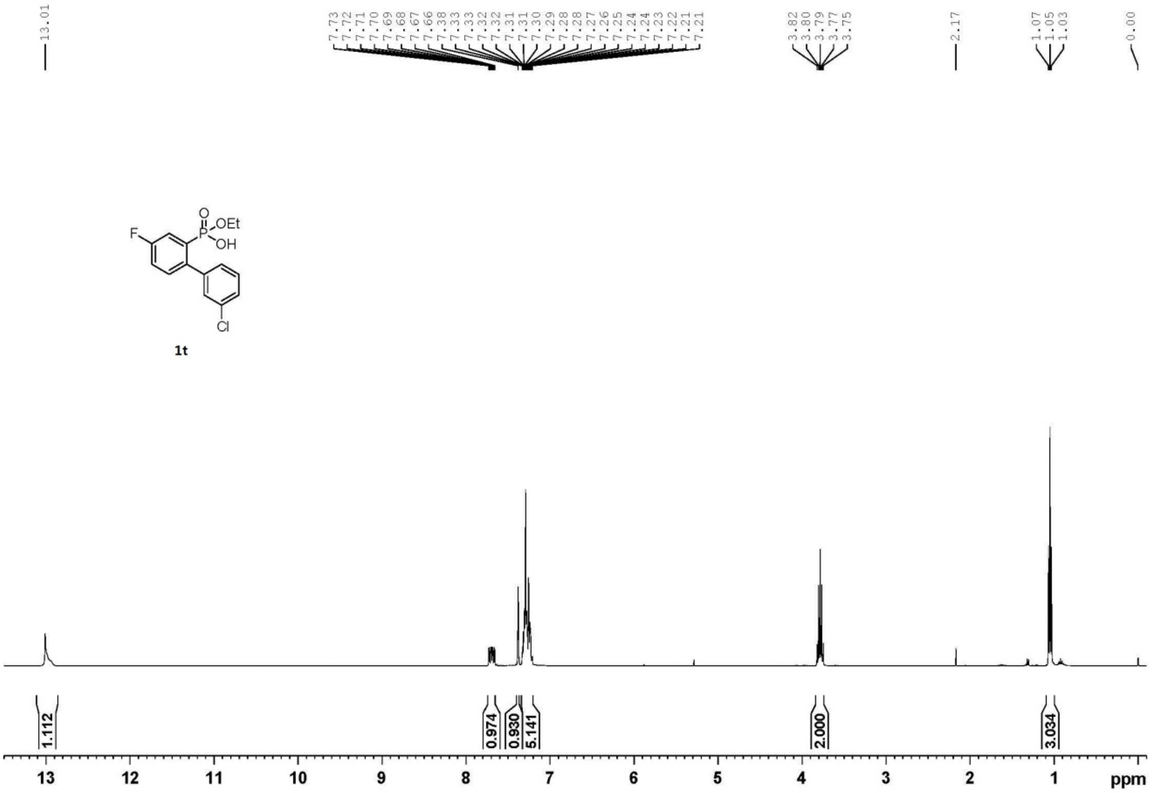
13.01
7.73 7.72 7.71 7.70 7.69 7.68 7.67 7.66 7.38 7.33 7.33 7.32 7.32 7.31 7.31 7.30 7.29 7.28 7.28 7.27 7.26 7.25 7.24 7.24 7.23 7.22 7.21 7.21
3.82 3.80 3.79 3.77 3.75
2.17
1.07 1.05 1.03
0.00
F
O
P
OEt
OH
Cl
1t
1.112
0.974
0.930
5.141
2.000
3.034
13 12 11 10 9 8 7 6 5 4 3 2 1 ppm

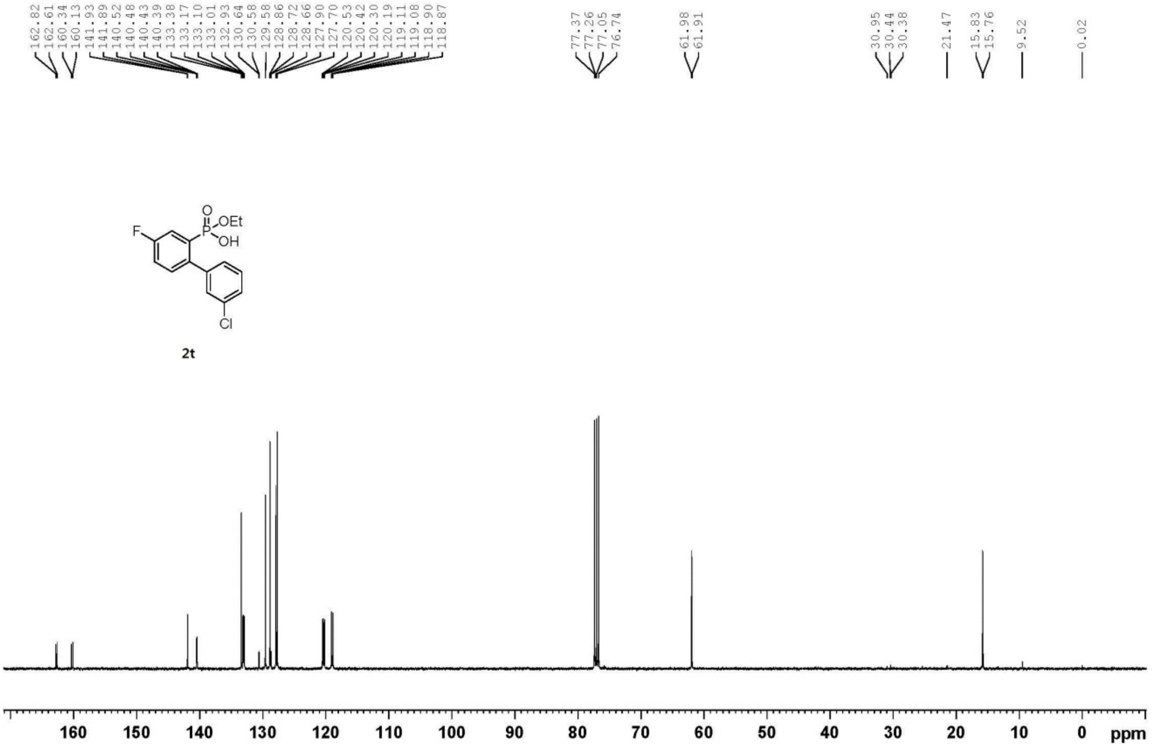
162.82 162.61 160.34 160.13 141.93 141.89 140.52 140.48 140.43 140.39 133.38 133.17 133.10 133.01 132.93 130.64 130.58 129.58 128.86 128.72 128.66 127.90 127.70 120.53 120.42 120.30 120.19 119.11 119.08 118.90 118.87
77.37 77.26 77.05 76.74
61.98 61.91
30.95 30.44 30.38
21.47
15.83 15.76
9.52
0.02
F
O
P
OEt
OH
Cl
2t
160 150 140 130 120 110 100 90 80 70 60 50 40 30 20 10 0 ppm

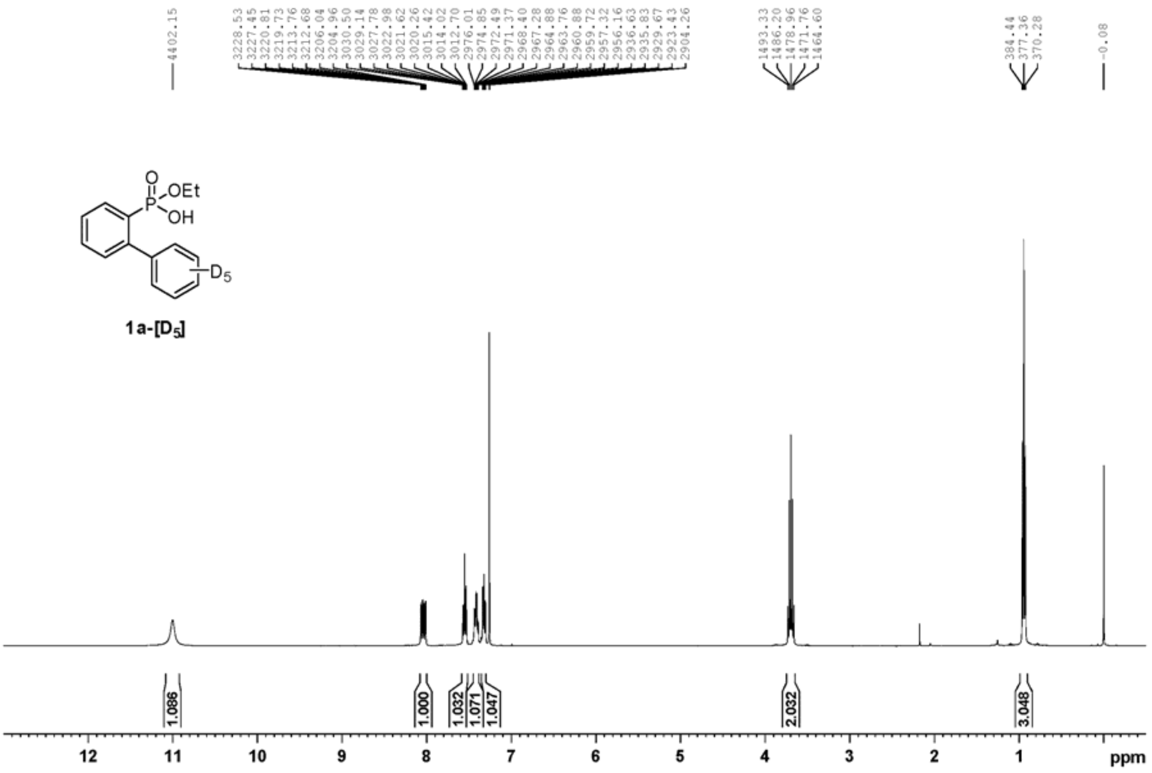
1a-[D5]
ppm

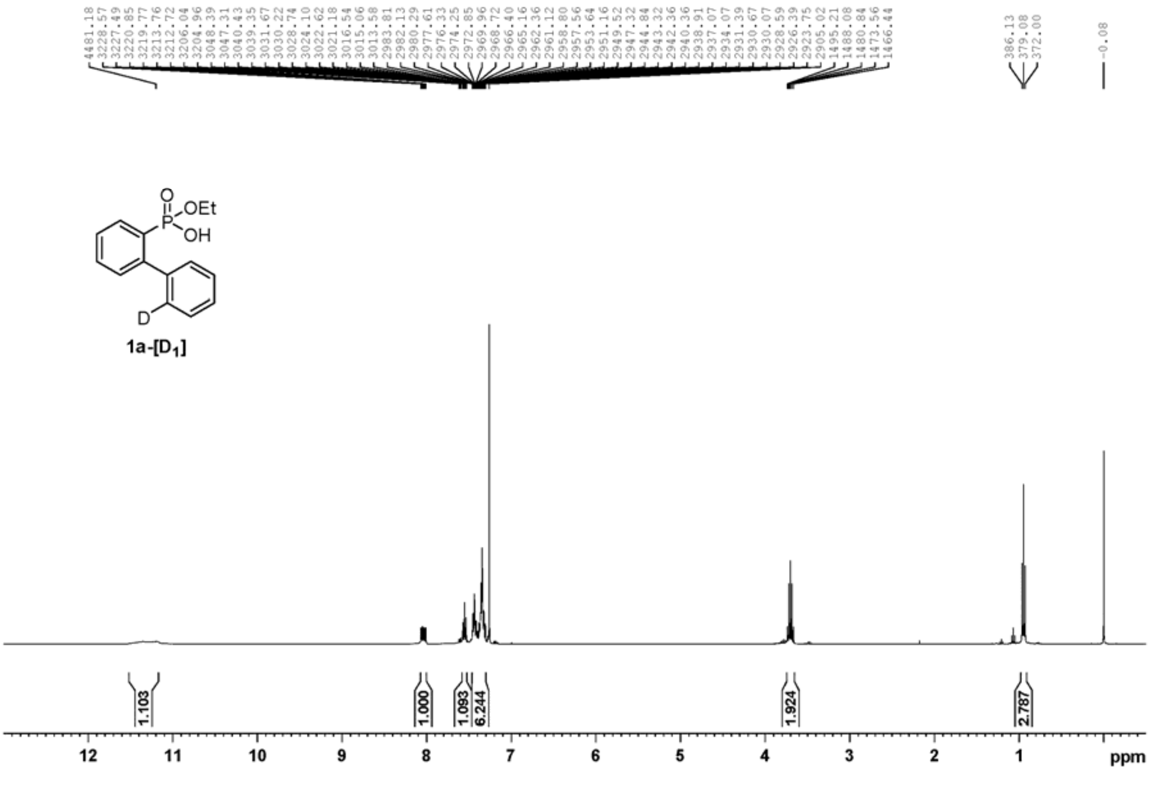
1a-[D1]
ppm

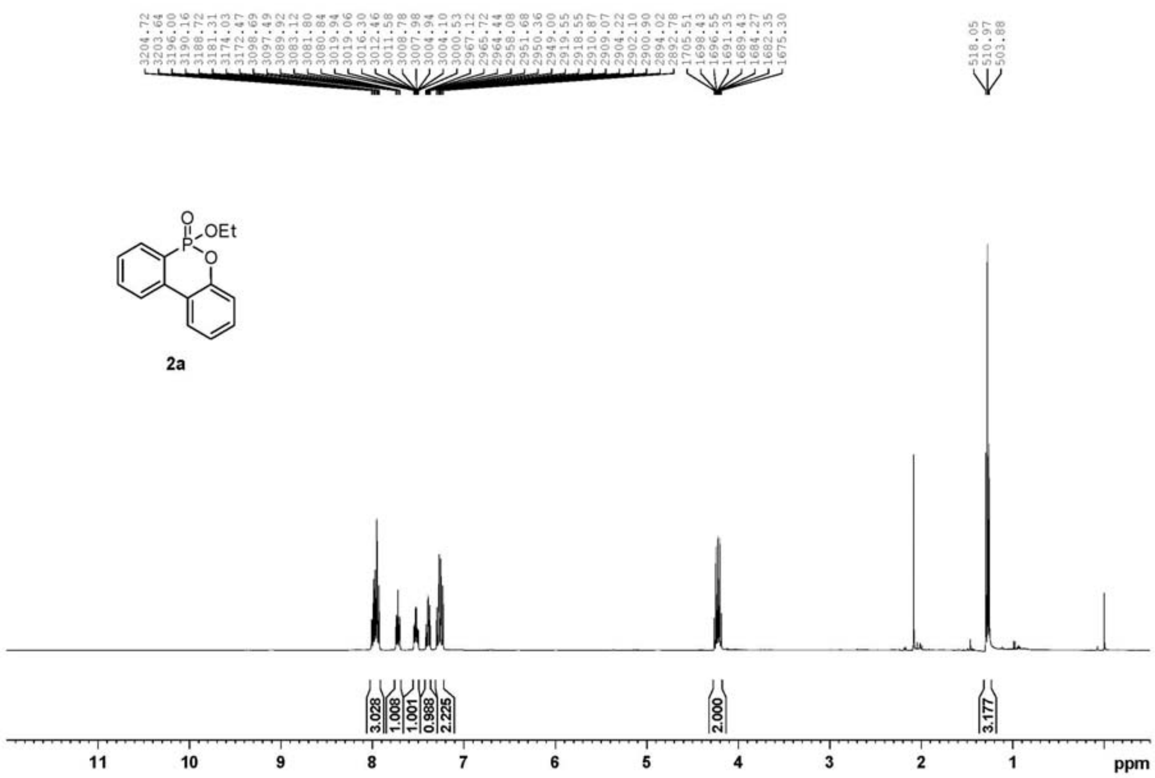
3204.72
3203.64
3196.00
3190.16
3188.72
3181.31
3174.03
3172.47
3098.69
3097.49
3089.92
3083.12
3081.80
3080.84
3019.94
3019.06
3016.30
3012.46
3011.58
3008.78
3007.98
3004.94
3004.10
3000.53
2967.12
2965.72
2964.44
2958.08
2951.68
2950.36
2949.00
2919.55
2918.55
2910.87
2909.07
2904.22
2902.10
2900.90
2894.02
2892.78
1705.51
1698.43
1696.55
1691.35
1689.43
1684.27
1682.35
1675.30
518.05
510.97
503.88
OEt
P
O
O
2a
3.028
1.008
1.001
0.988
2.225
2.000
3.177
11
10
9
8
7
6
5
4
3
2
1
ppm

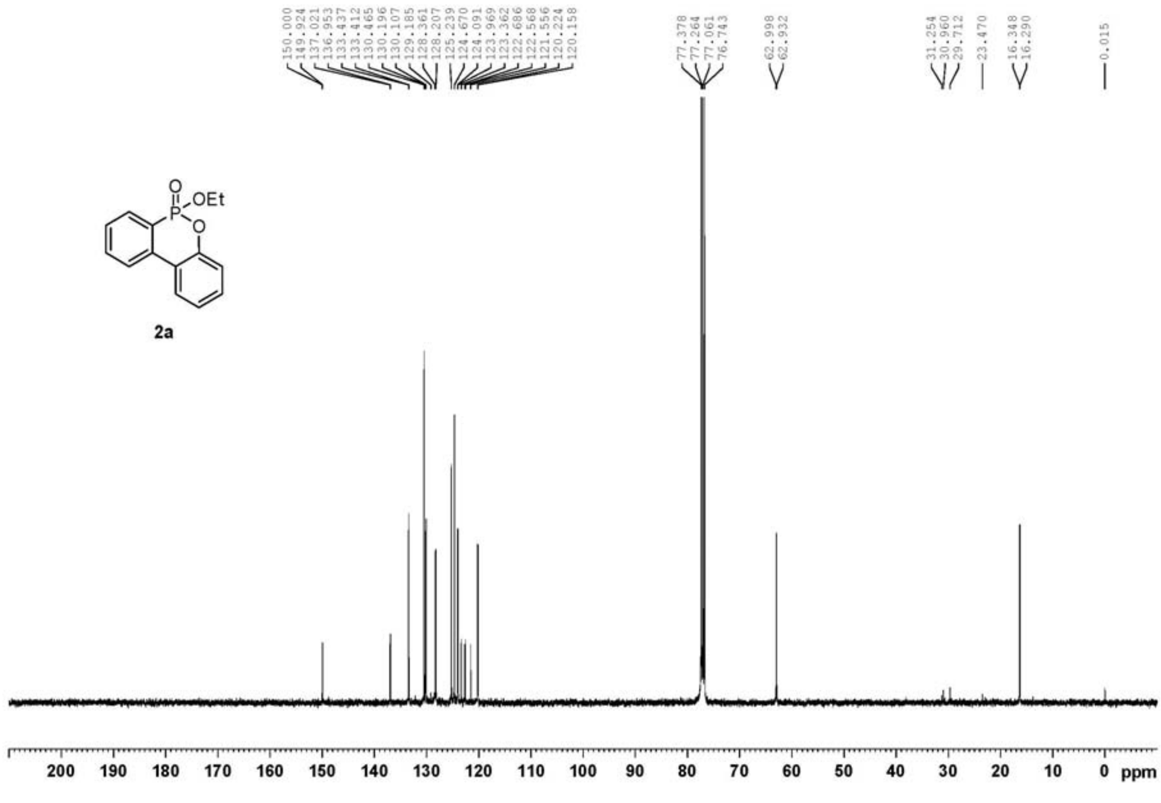
150.000
149.924
137.021
136.953
133.437
133.412
130.465
130.196
130.107
129.185
128.361
128.207
125.239
124.670
124.091
123.969
123.362
122.686
122.568
121.556
120.224
120.158
77.378
77.264
77.061
76.743
62.998
62.932
31.254
30.960
29.712
23.470
16.348
16.290
0.015
OEt
P
O
O
2a
200
190
180
170
160
150
140
130
120
110
100
90
80
70
60
50
40
30
20
10
0
ppm

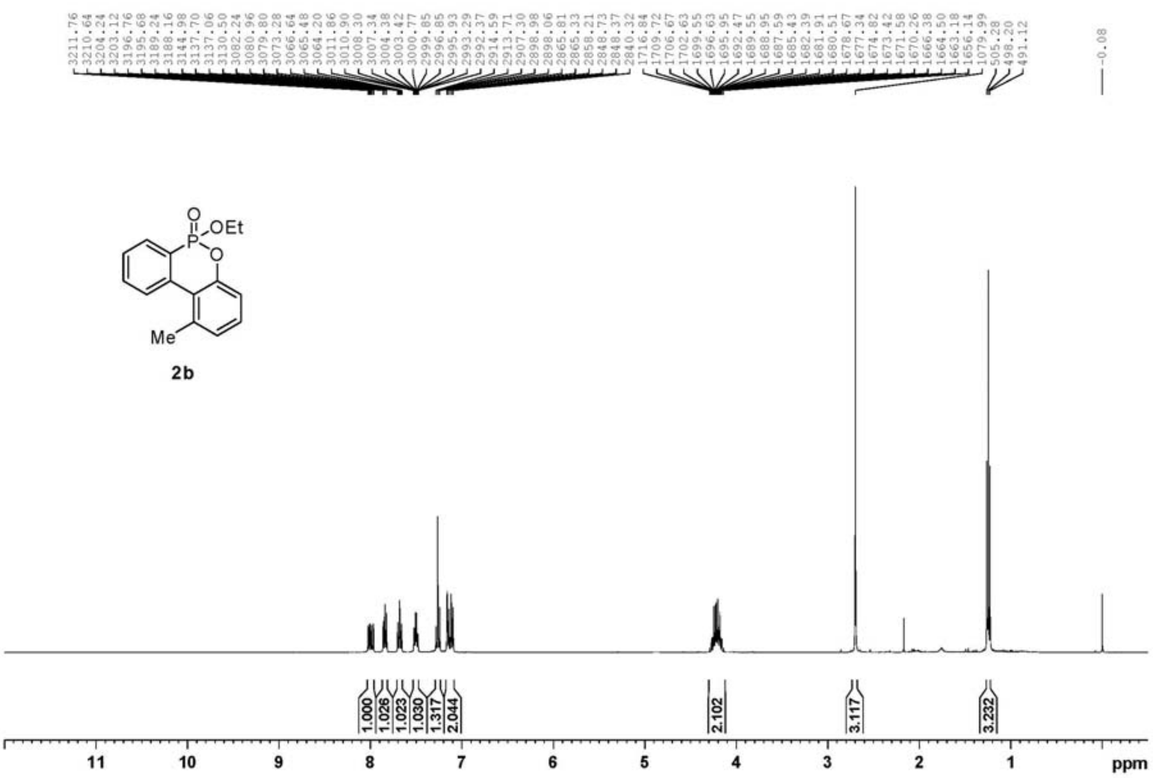
ppm

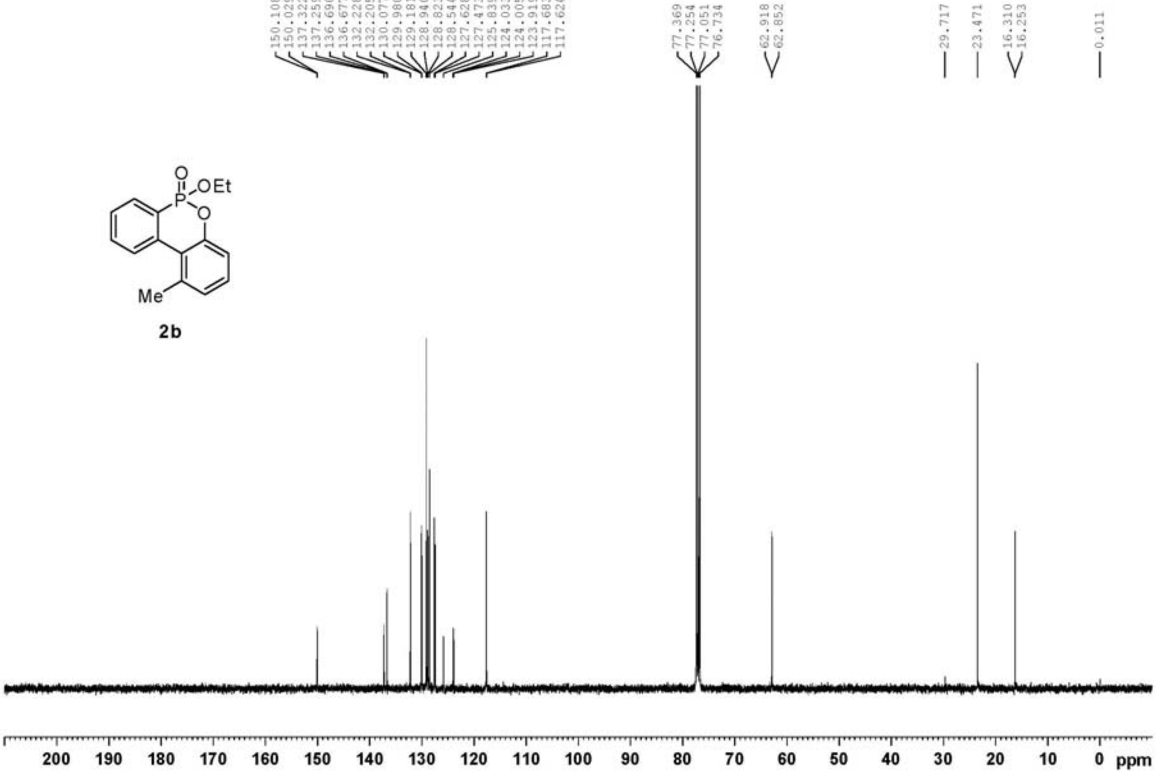
ppm

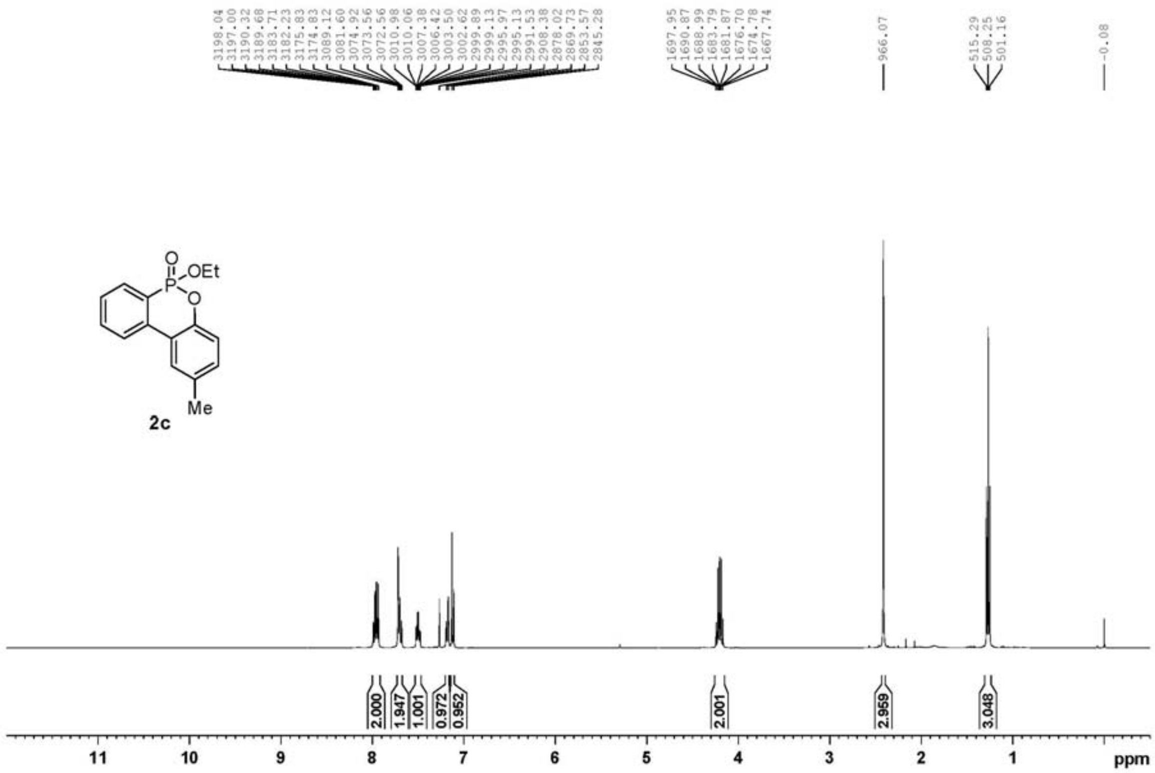
3198.04
3197.00
3190.32
3189.68
3183.71
3182.23
3175.83
3174.83
3089.12
3081.60
3074.92
3073.56
3072.56
3010.98
3010.06
3007.38
3006.42
3003.50
3002.62
2999.89
2999.13
2995.97
2995.13
2991.53
2908.38
2878.02
2869.73
2853.57
2845.28
1697.95
1690.87
1688.99
1683.79
1681.87
1676.70
1674.78
1667.74
966.07
515.29
508.25
501.16
-0.08
OEt
Me
2c
2.000
1.947
1.001
0.972
0.952
2.001
2.959
3.048
ppm

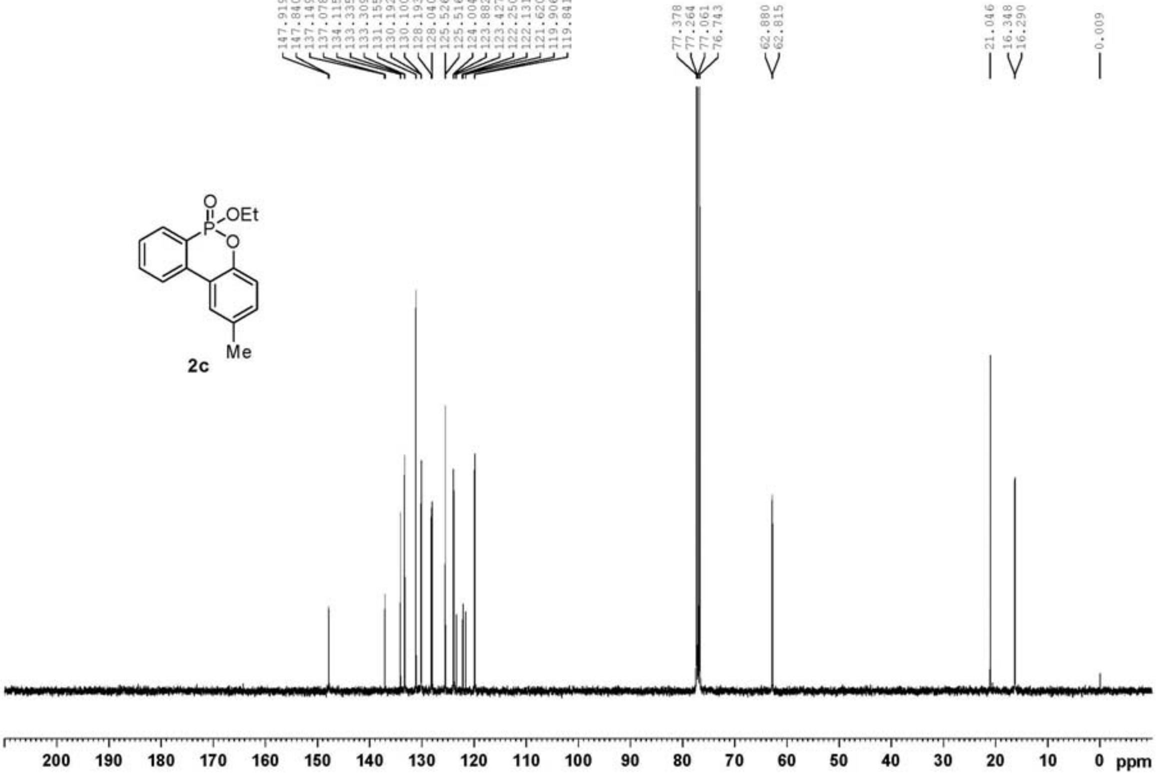
147.919
147.840
137.149
137.078
134.115
133.335
133.309
131.155
130.192
130.100
128.193
128.040
125.526
125.516
124.004
123.882
123.427
122.250
122.131
121.620
119.906
119.841
77.378
77.264
77.061
76.743
62.880
62.815
21.046
16.348
16.290
0.009
OEt
Me
2c
ppm

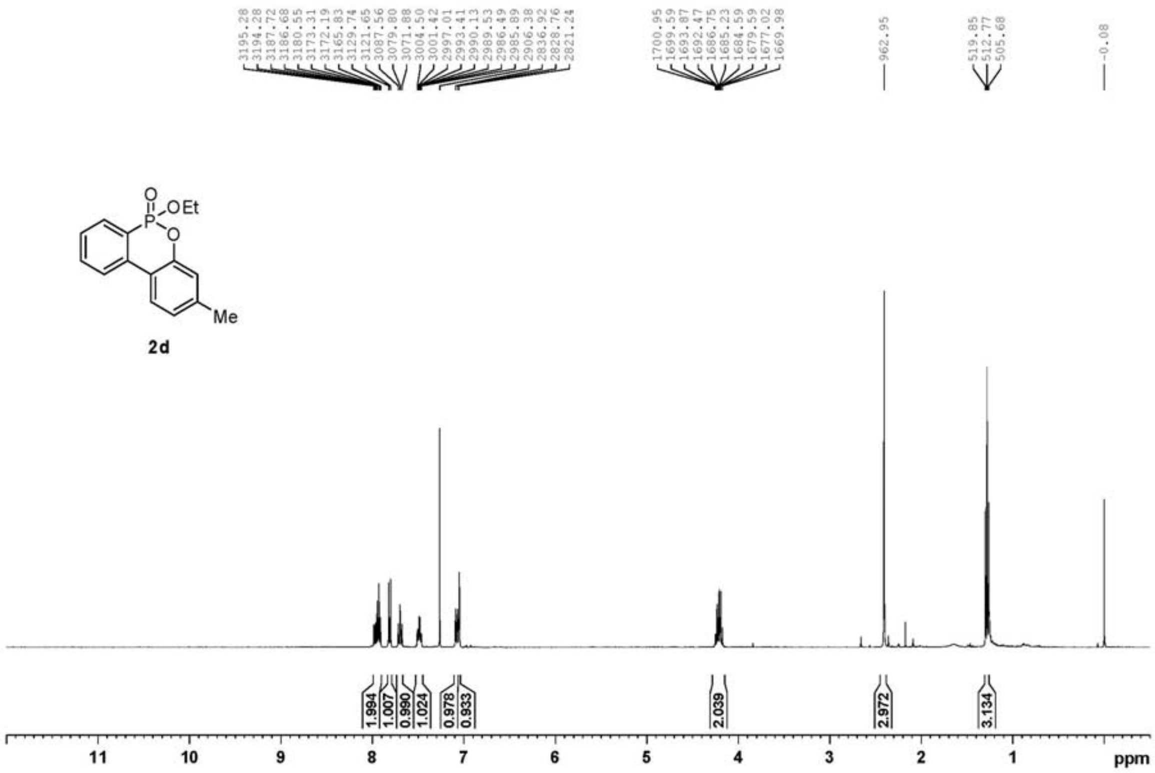
3195.28
3194.28
3187.72
3186.68
3180.55
3173.31
3172.19
3165.83
3129.74
3121.65
3087.56
3079.80
3071.88
3004.50
3001.42
2997.01
2993.41
2990.13
2989.53
2986.49
2985.89
2906.38
2836.92
2828.76
2821.24
1700.95
1699.59
1693.87
1692.47
1686.75
1685.23
1684.59
1679.59
1677.02
1669.98
962.95
519.85
512.77
505.68
-0.08
OEt
O
P
O
Me
2d
1.994
1.007
0.990
1.024
0.978
0.933
2.039
2.972
3.134
11
10
9
8
7
6
5
4
3
2
1
ppm

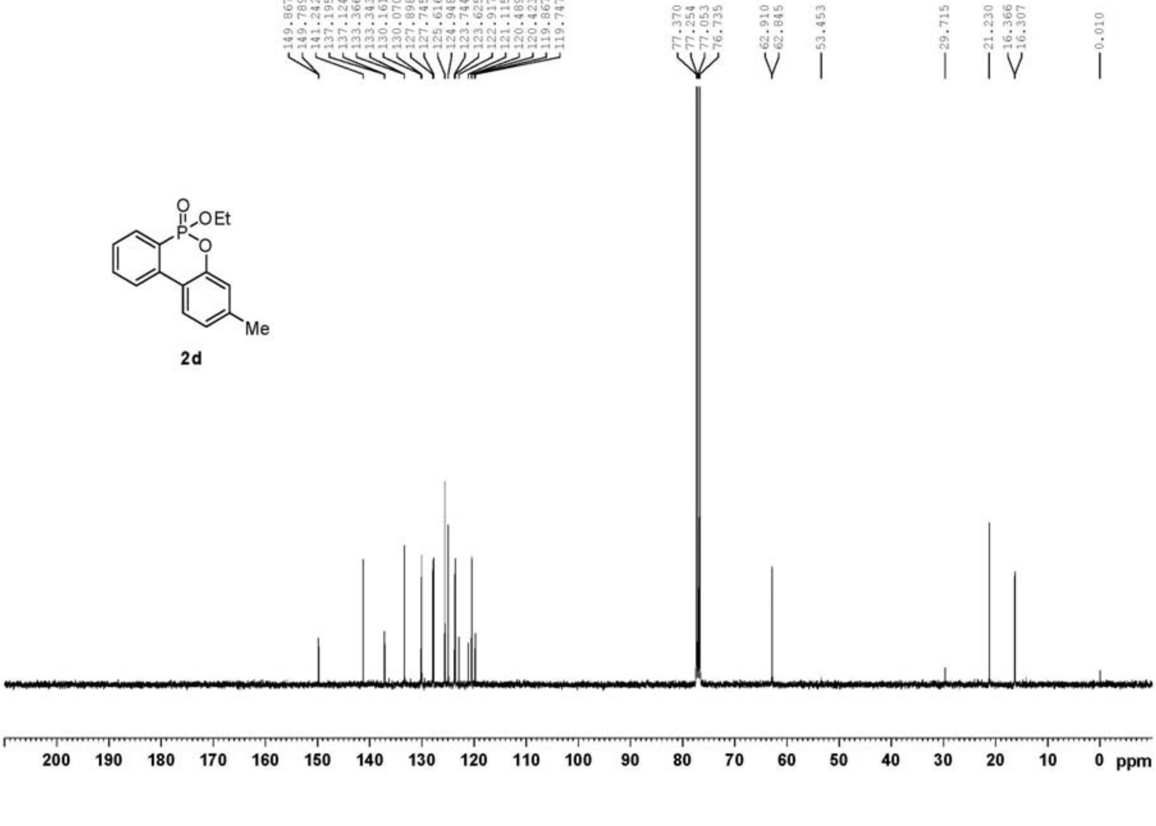
149.867
149.789
141.242
137.195
137.124
133.366
133.343
130.161
130.070
127.898
127.745
125.616
124.948
123.744
123.625
122.917
121.115
120.489
120.423
119.867
119.747
77.370
77.254
77.053
76.735
62.910
62.845
53.453
29.715
21.230
16.366
16.307
0.010
OEt
O
P
O
Me
2d
200
190
180
170
160
150
140
130
120
110
100
90
80
70
60
50
40
30
20
10
0
ppm

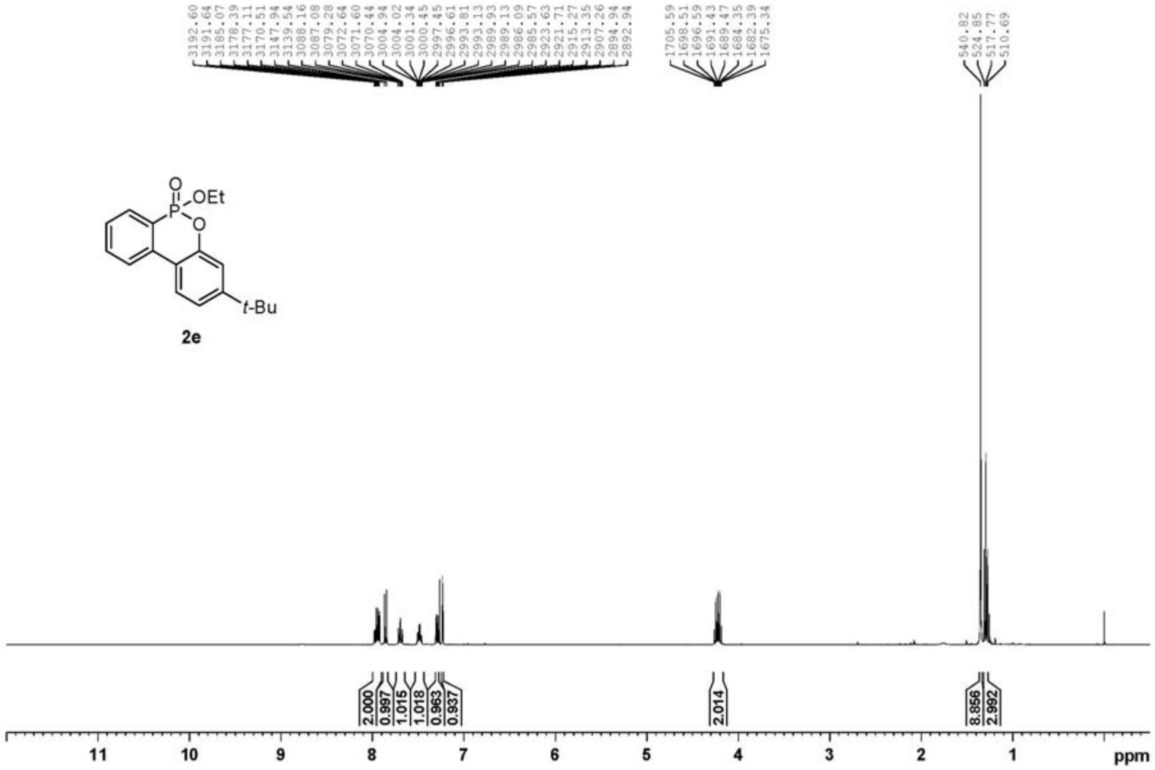
3192.60
3191.64
3185.07
3178.39
3177.11
3170.51
3147.94
3139.54
3088.16
3087.08
3079.28
3072.64
3071.60
3070.44
3004.94
3004.02
3001.34
3000.45
2997.45
2996.61
2993.81
2993.13
2989.93
2989.13
2986.09
2985.57
2923.63
2921.71
2915.27
2913.35
2907.26
2894.94
2892.94
1705.59
1698.51
1696.59
1691.43
1689.47
1684.35
1682.39
1675.34
540.82
524.85
517.77
510.69
OEt
O
P
O
t-Bu
2e
2.000
0.997
1.015
1.018
0.963
0.937
2.014
8.856
2.992
11
10
9
8
7
6
5
4
3
2
1
ppm

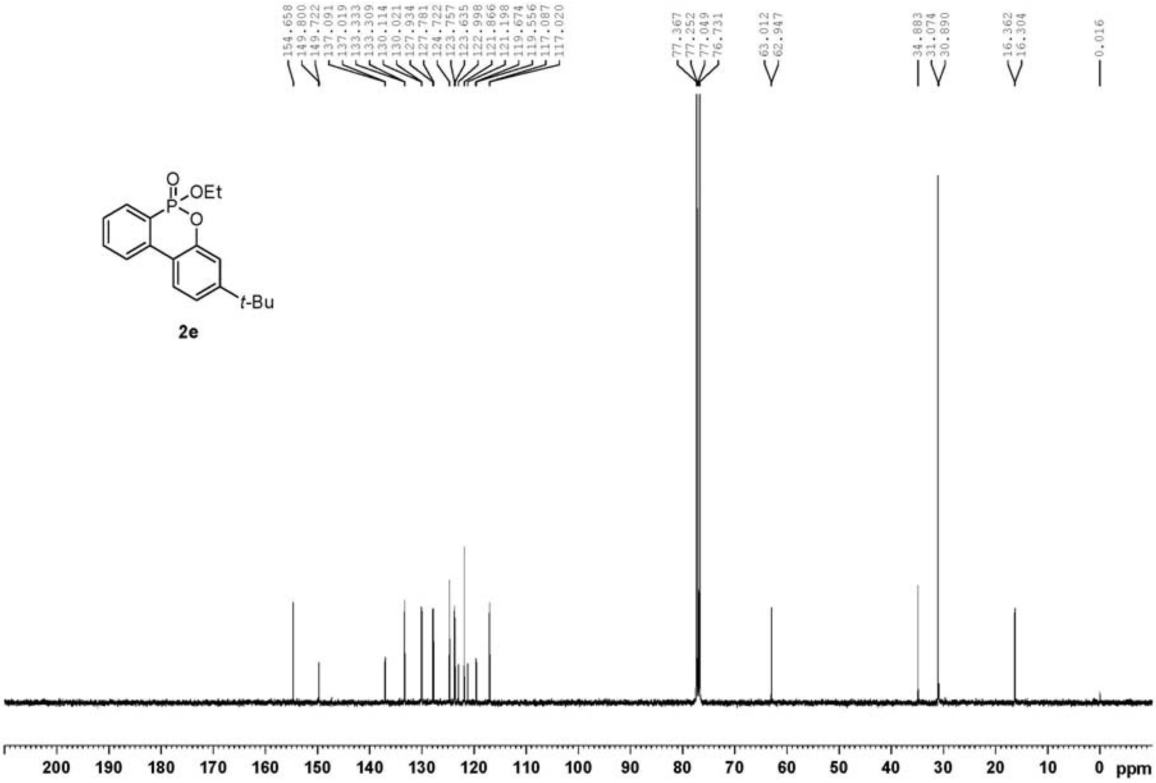
154.658
149.800
149.722
137.091
137.019
133.333
133.309
130.114
130.021
127.934
127.781
124.722
123.757
123.635
122.998
121.866
121.198
119.674
119.556
117.087
117.020
77.367
77.252
77.049
76.731
63.012
62.947
34.883
31.074
30.890
16.362
16.304
0.016
OEt
O
P
O
t-Bu
2e
200
190
180
170
160
150
140
130
120
110
100
90
80
70
60
50
40
30
20
10
0
ppm

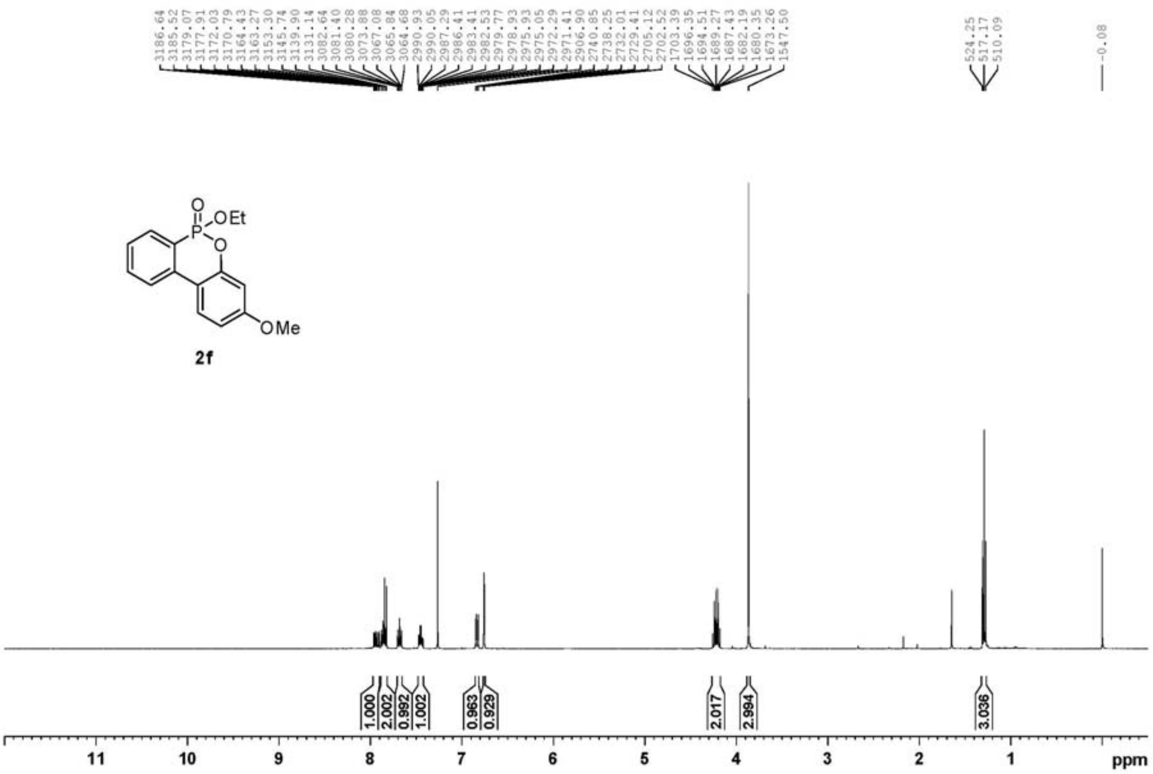

3186.64
3185.52
3179.07
3177.91
3172.03
3170.79
3164.43
3163.27
3153.30
3145.74
3139.90
3131.14
3082.64
3081.40
3080.28
3073.88
3067.08
3065.84
3064.68
2990.93
2990.05
2987.29
2986.41
2983.41
2982.53
2979.77
2978.93
2975.93
2975.05
2972.29
2971.41
2906.90
2740.85
2738.25
2732.01
2729.41
2705.12
2702.52
1703.39
1696.35
1694.51
1689.27
1687.43
1682.19
1680.35
1673.26
1547.50
524.25
517.17
510.09
-0.08
OEt
O
P
O
OMe
2f
1.000
2.002
0.992
1.002
0.963
0.929
2.017
2.994
3.036
11
10
9
8
7
6
5
4
3
2
1
ppm

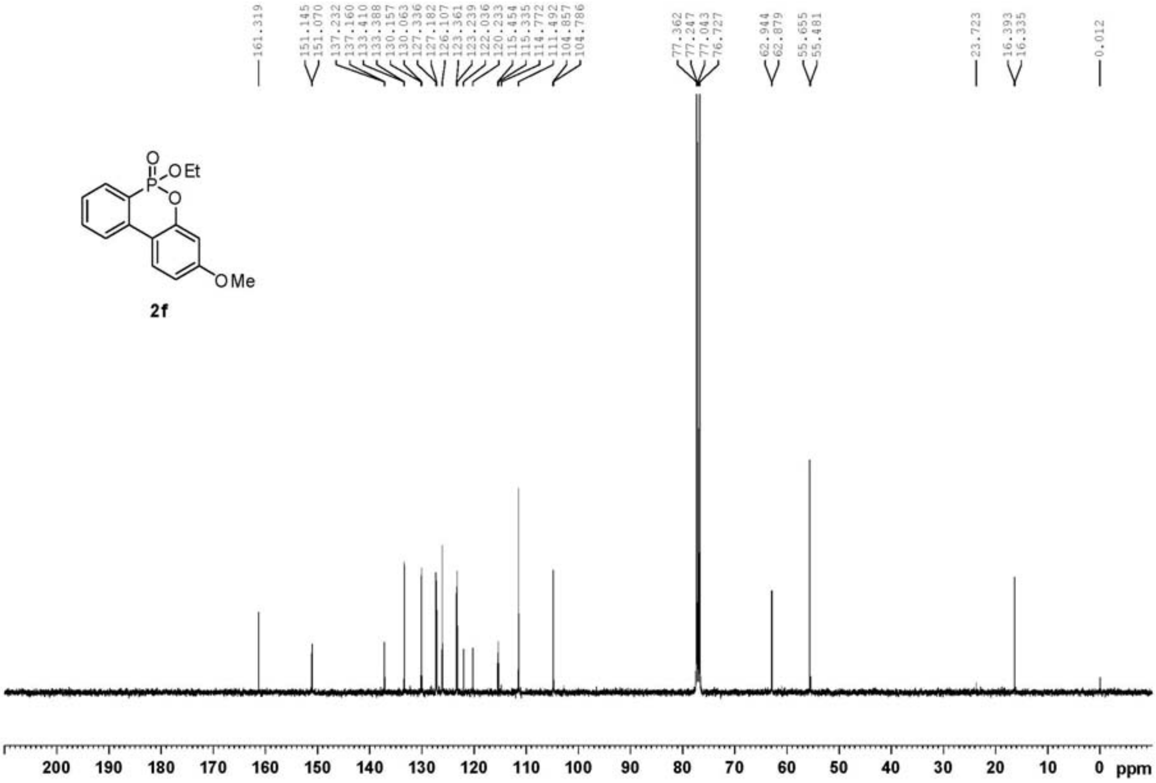

161.319
151.145
151.070
137.232
137.160
133.410
133.388
130.157
130.063
127.336
127.182
126.107
123.361
123.239
122.036
120.233
115.454
115.335
114.772
111.492
104.857
104.786
77.362
77.247
77.043
76.727
62.944
62.879
55.655
55.481
23.723
16.393
16.335
0.012
OEt
O
P
O
OMe
2f
200
190
180
170
160
150
140
130
120
110
100
90
80
70
60
50
40
30
20
10
0
ppm

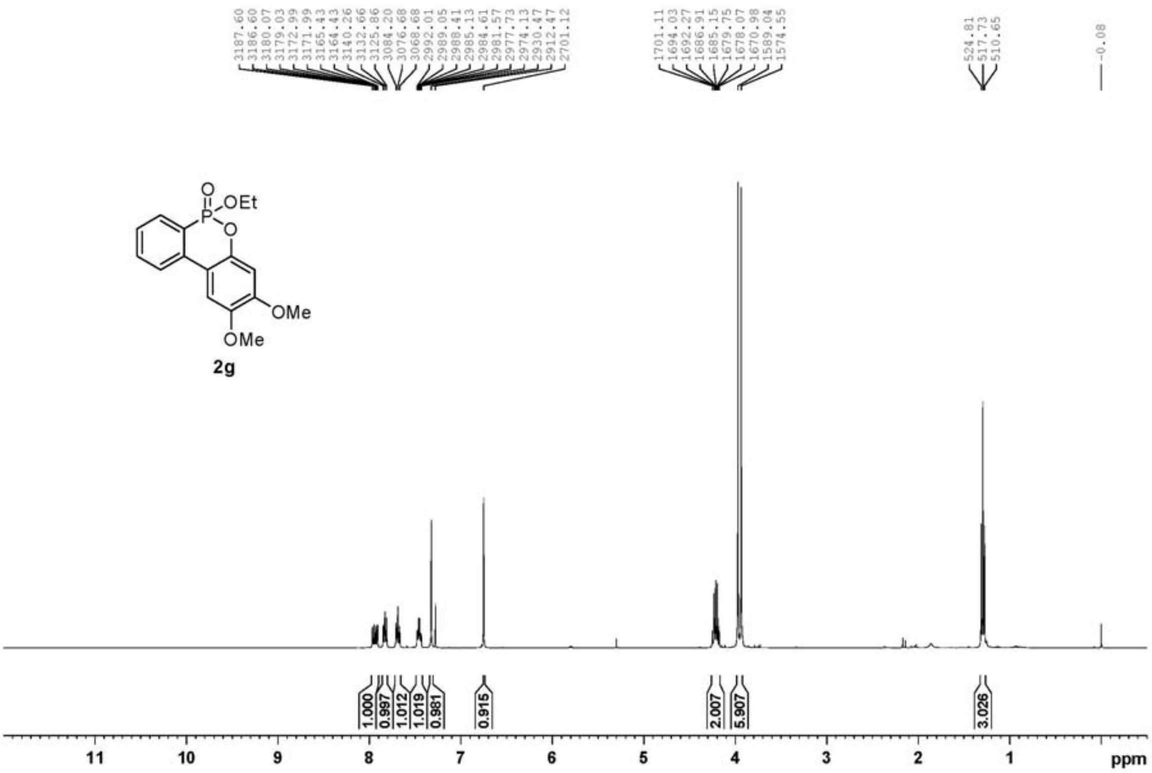
3187.60
3186.60
3180.07
3179.03
3172.99
3171.99
3165.43
3164.43
3140.26
3132.66
3125.86
3084.20
3076.68
3068.68
2992.01
2989.05
2988.41
2985.13
2984.61
2981.57
2977.73
2974.13
2930.47
2912.47
2701.12
1701.11
1694.03
1692.27
1686.91
1685.15
1679.75
1678.07
1670.98
1589.04
1574.55
524.81
517.73
510.65
-0.08
OEt
OMe
OMe
2g
1.000
0.997
1.012
1.019
0.981
0.915
2.007
5.907
3.026
11 10 9 8 7 6 5 4 3 2 1 ppm

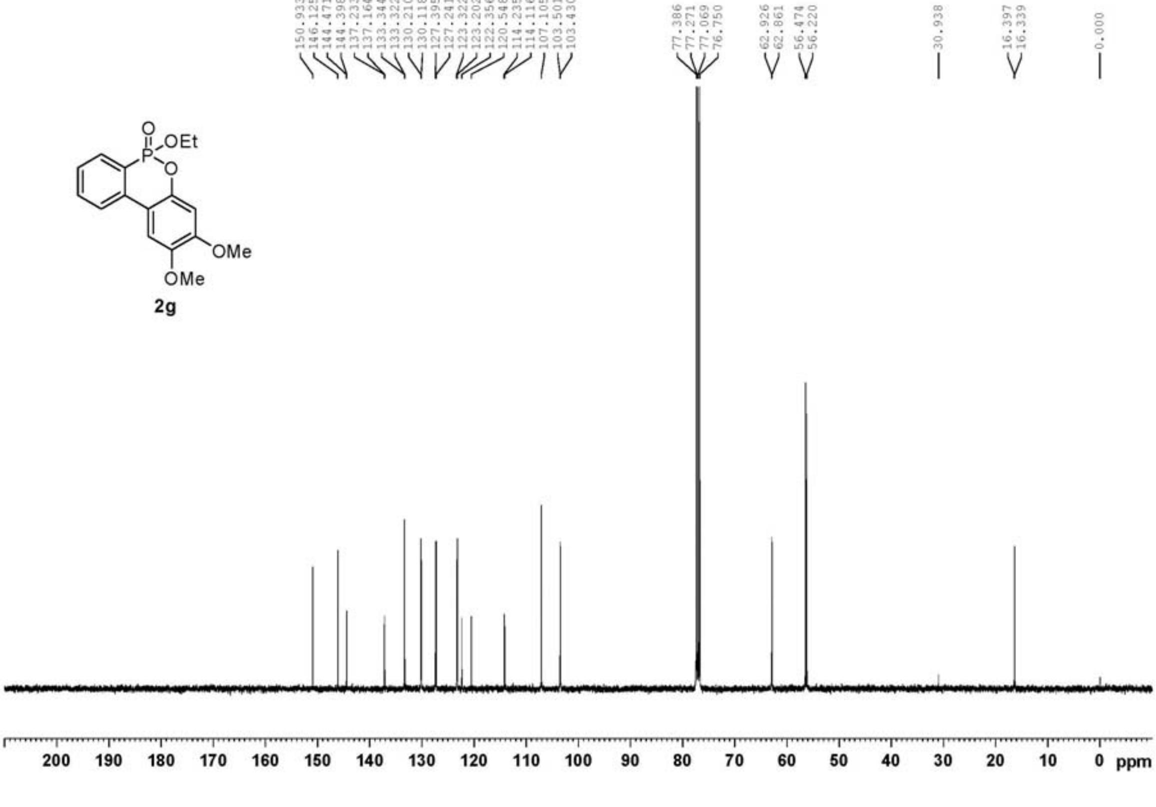
150.933
146.125
144.471
144.398
137.233
137.164
133.344
133.322
130.210
130.118
127.395
127.241
123.322
123.202
122.356
120.548
114.235
114.116
107.105
103.501
103.430
77.386
77.271
77.069
76.750
62.926
62.861
56.474
56.220
30.938
16.397
16.339
0.000
OEt
OMe
OMe
2g
200 190 180 170 160 150 140 130 120 110 100 90 80 70 60 50 40 30 20 10 0 ppm

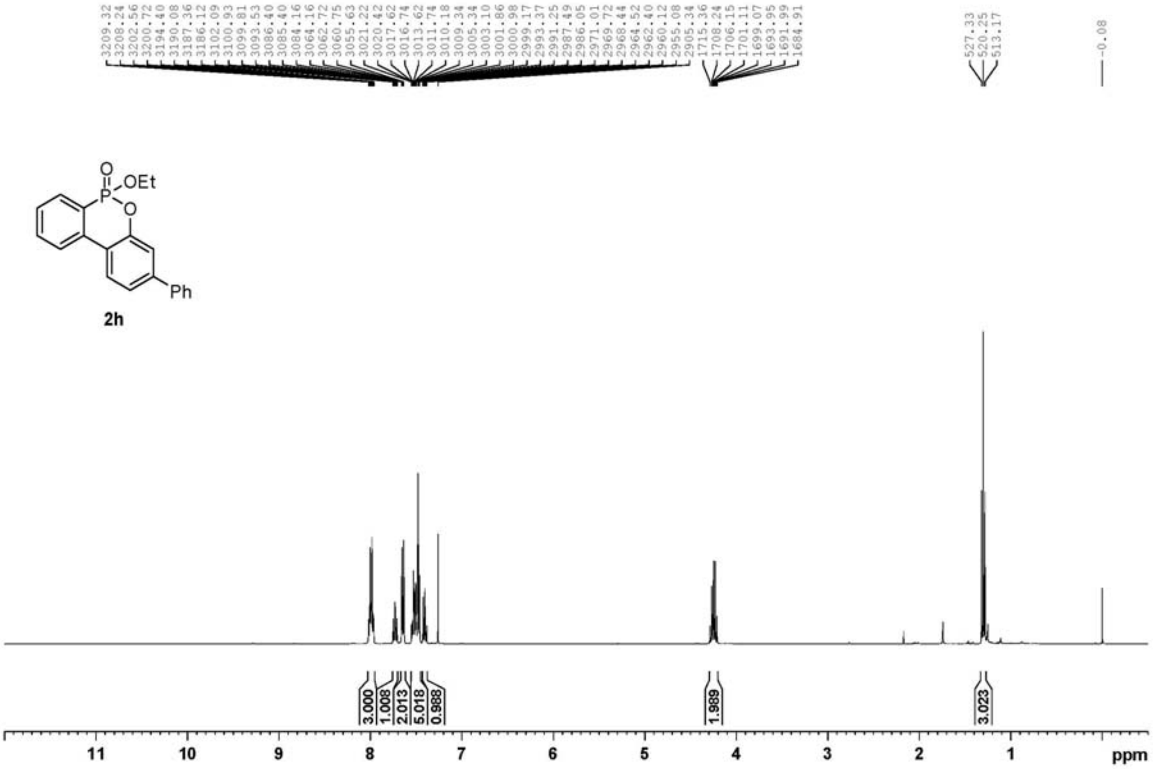
3209.32
3208.24
3202.56
3200.72
3194.40
3190.08
3187.36
3186.12
3102.09
3100.93
3099.81
3093.53
3086.40
3085.40
3084.16
3064.16
3062.72
3060.75
3055.63
3021.22
3020.42
3017.62
3016.74
3013.62
3011.74
3010.18
3009.34
3005.34
3003.10
3001.86
3000.98
2999.17
2993.37
2991.25
2987.49
2986.05
2971.01
2969.72
2968.44
2964.52
2962.40
2960.12
2955.08
2905.34
1715.36
1708.24
1706.15
1701.11
1699.07
1693.95
1691.99
1684.91
527.33
520.25
513.17
-0.08
OEt
O
P
O
Ph
2h
3.000
1.008
2.013
5.018
0.988
1.989
3.023
11
10
9
8
7
6
5
4
3
2
1
ppm

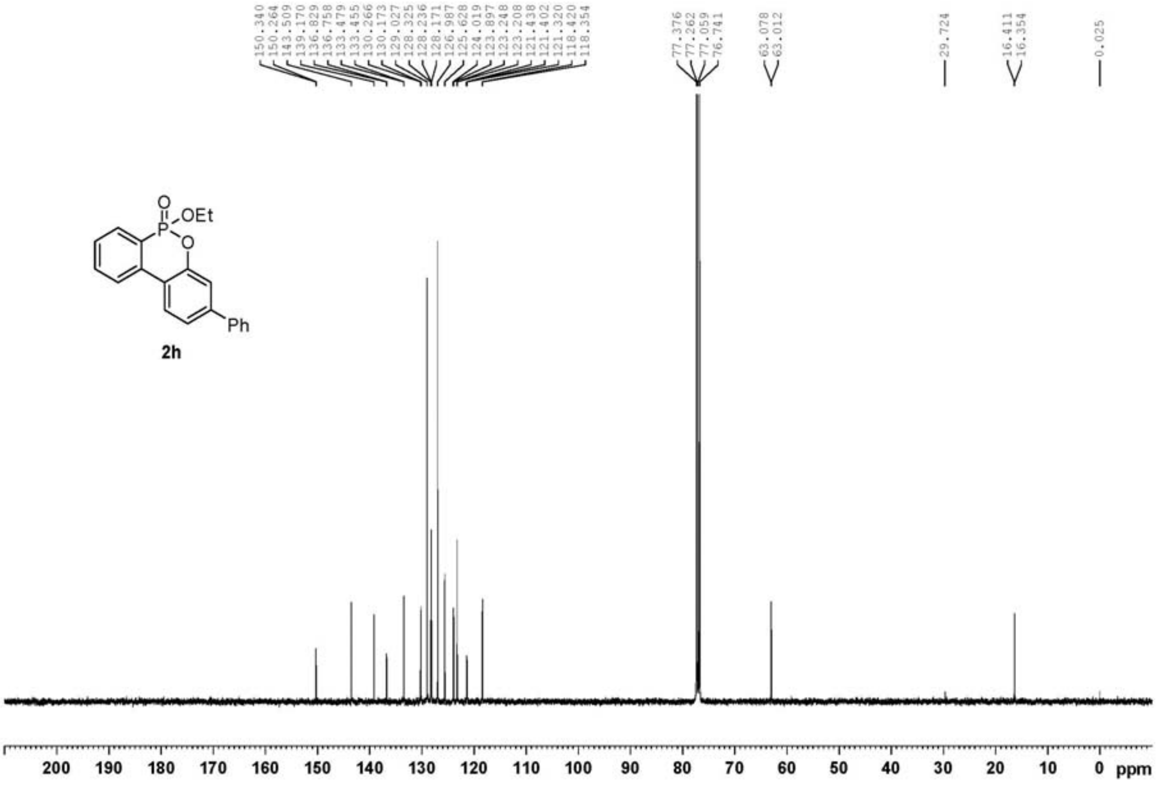
150.340
150.264
143.509
139.170
136.829
136.758
133.479
133.455
130.266
130.173
129.027
128.325
128.236
128.171
126.987
125.628
124.019
123.897
123.248
123.208
121.438
121.402
121.320
118.420
118.354
77.376
77.262
77.059
76.741
63.078
63.012
29.724
16.411
16.354
0.025
OEt
O
P
O
Ph
2h
200
190
180
170
160
150
140
130
120
110
100
90
80
70
60
50
40
30
20
10
0
ppm

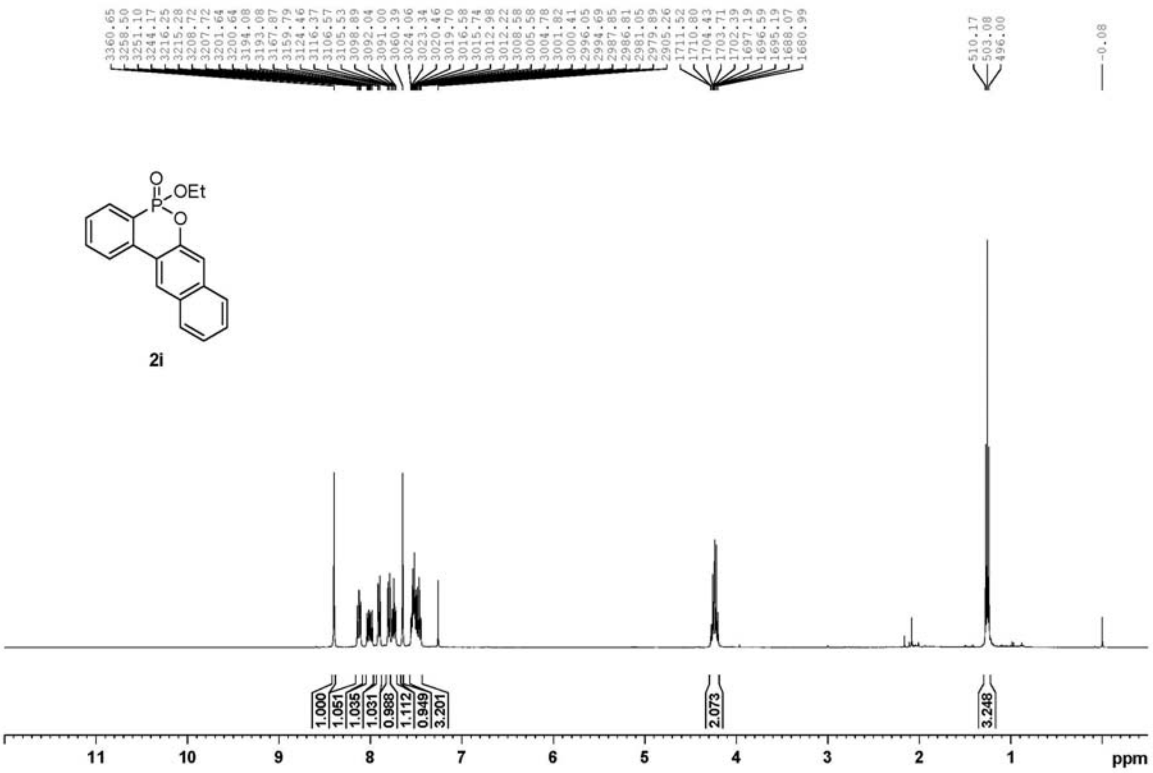
3360.65
3258.50
3251.10
3244.17
3216.25
3215.28
3208.72
3207.72
3201.64
3200.64
3194.08
3193.08
3167.87
3159.79
3124.46
3116.37
3106.57
3105.53
3098.89
3092.04
3091.00
3060.39
3024.06
3023.34
3020.46
3019.70
3016.58
3015.74
3012.98
3012.22
3008.58
3005.58
3004.78
3001.82
3000.41
2996.05
2994.69
2987.85
2986.81
2981.05
2979.89
2905.26
1711.52
1710.80
1704.43
1703.71
1702.39
1697.19
1696.59
1695.19
1688.07
1680.99
510.17
503.08
496.00
-0.08
O
P
OEt
O
2i
1.000
1.051
1.035
1.031
0.988
1.112
0.949
3.201
2.073
3.248
11
10
9
8
7
6
5
4
3
2
1
ppm

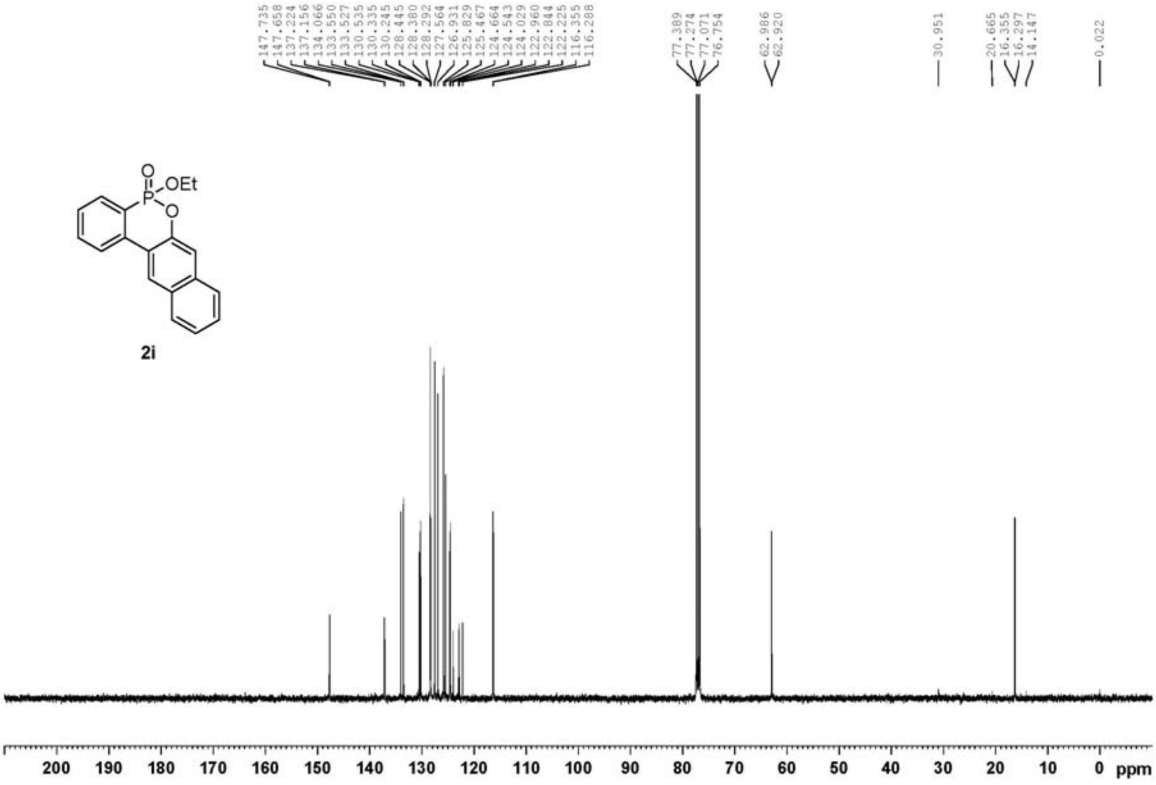
147.735
147.658
137.224
137.156
134.066
133.550
133.527
130.535
130.335
130.245
128.445
128.380
128.292
127.564
126.931
125.829
125.467
124.664
124.543
124.029
122.960
122.844
122.225
116.355
116.288
77.389
77.274
77.071
76.754
62.986
62.920
30.951
20.665
16.355
16.297
14.147
0.022
O
P
OEt
O
2i
200
190
180
170
160
150
140
130
120
110
100
90
80
70
60
50
40
30
20
10
0
ppm

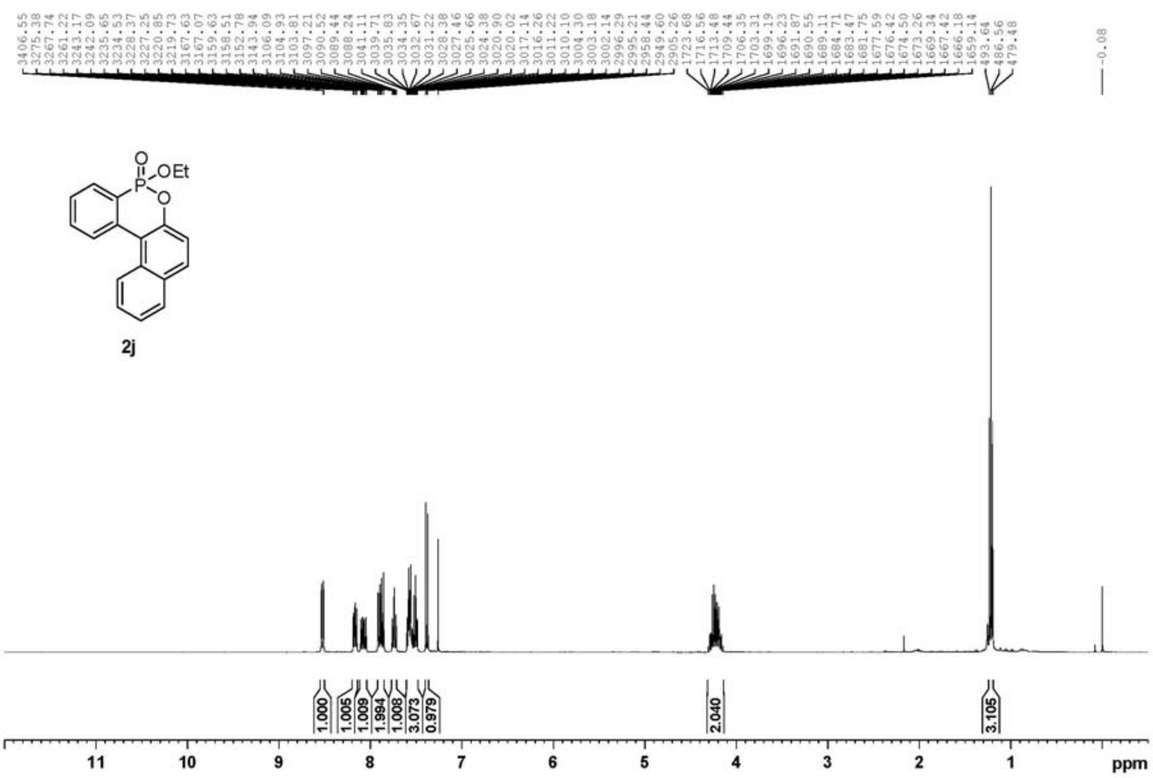
3406.55
3275.38
3267.74
3261.22
3243.17
3242.09
3235.65
3234.53
3228.37
3227.25
3220.85
3219.73
3167.63
3167.07
3159.63
3158.51
3152.78
3143.94
3106.09
3104.93
3103.81
3097.21
3090.52
3089.44
3088.24
3041.11
3039.71
3035.83
3034.35
3032.67
3031.22
3028.38
3027.46
3025.66
3024.38
3020.90
3020.02
3017.14
3016.26
3011.22
3010.10
3004.30
3003.18
3002.14
2996.29
2995.21
2958.44
2949.60
2905.26
1723.68
1716.56
1713.48
1709.44
1706.35
1703.31
1699.19
1696.23
1691.87
1690.55
1689.11
1684.71
1683.47
1681.75
1677.59
1676.42
1674.50
1673.26
1669.34
1667.42
1666.18
1659.14
493.64
486.56
479.48
-0.08
O
P
OEt
O
2j
1.000
1.005
1.009
1.994
1.008
3.073
0.979
2.040
3.105
11
10
9
8
7
6
5
4
3
2
1
ppm

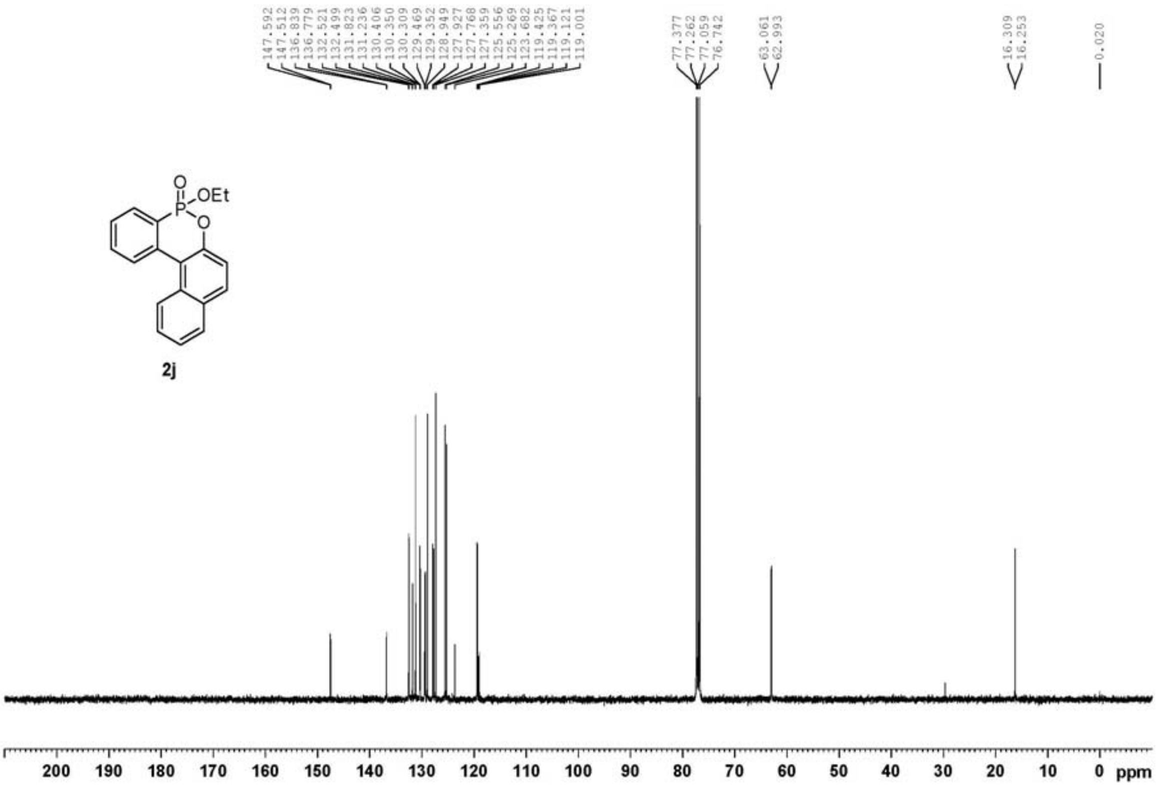
147.592
147.512
136.839
136.779
132.521
132.499
131.823
131.236
130.406
130.350
130.309
129.469
129.352
128.949
127.927
127.768
127.359
125.556
125.269
123.682
119.425
119.367
119.121
119.001
77.377
77.262
77.059
76.742
63.061
62.993
16.309
16.253
0.020
O
P
OEt
O
2j
200
190
180
170
160
150
140
130
120
110
100
90
80
70
60
50
40
30
20
10
0
ppm

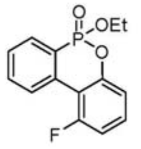
O
P
OEt
O
F

2k

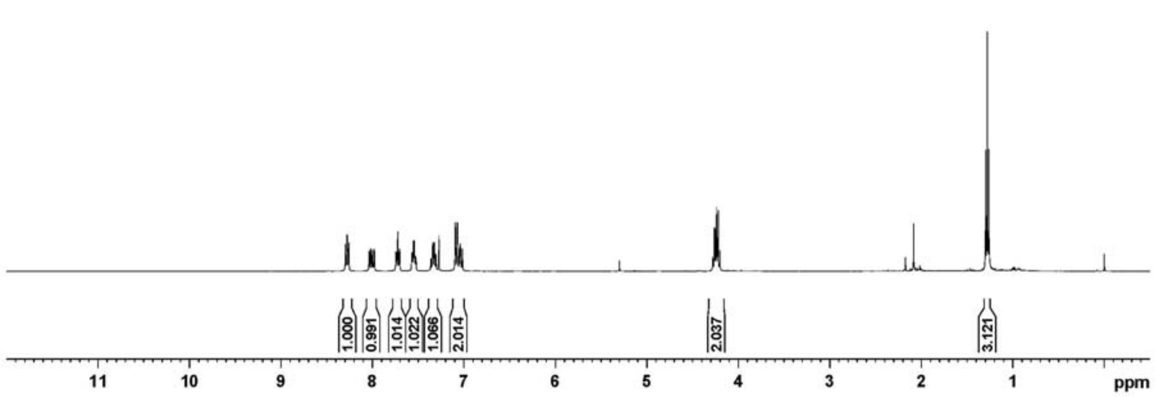
3319.04
3311.68
3304.43
3215.80
3214.72
3208.28
3207.16
3200.84
3199.72
3193.32
3192.20
3097.73
3090.04
3082.12
3029.42
3028.26
3025.90
3024.90
3021.54
3020.94
3018.02
3014.06
3010.54
2945.76
2944.84
2937.51
2936.59
2931.51
2930.55
2923.27
2922.39
2910.27
2838.56
2830.12
2829.20
2820.04
2816.28
2807.91
1712.24
1711.60
1705.07
1704.51
1702.99
1697.39
1695.79
1688.67
1681.59
519.21
512.13
505.04
-0.08
1.000
0.991
1.014
1.022
1.066
2.014
2.037
3.121
11
10
9
8
7
6
5
4
3
2
1
ppm

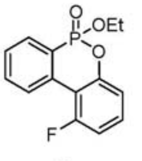
O
P
OEt
O
F

2k

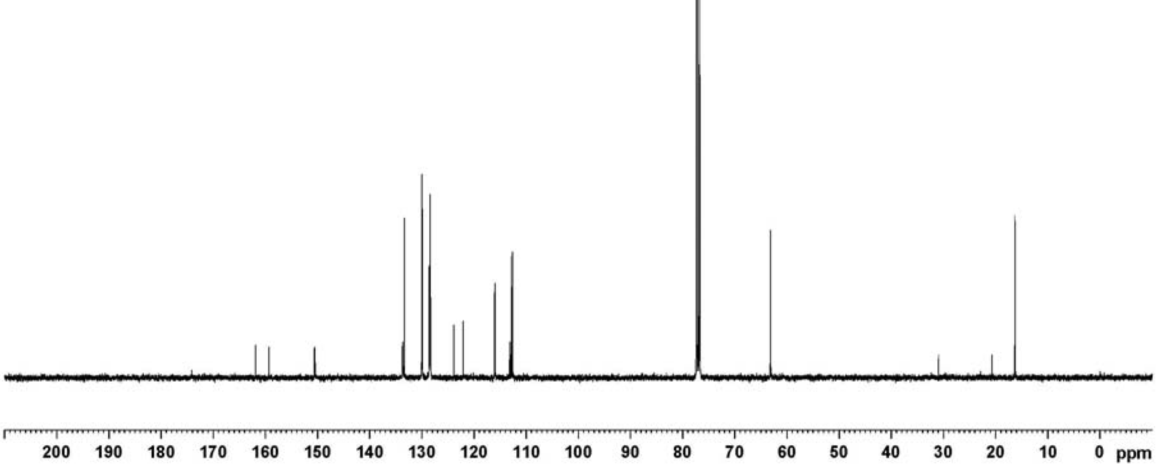
161.874
159.340
150.625
150.565
150.487
133.768
133.729
133.708
133.666
133.382
130.039
129.954
129.917
128.628
128.586
128.515
128.437
128.322
123.904
122.089
116.062
116.028
115.999
115.967
113.245
113.124
113.003
112.873
112.628
77.380
77.265
77.063
76.744
63.207
63.141
16.320
16.263
0.002
200
190
180
170
160
150
140
130
120
110
100
90
80
70
60
50
40
30
20
10
0 ppm

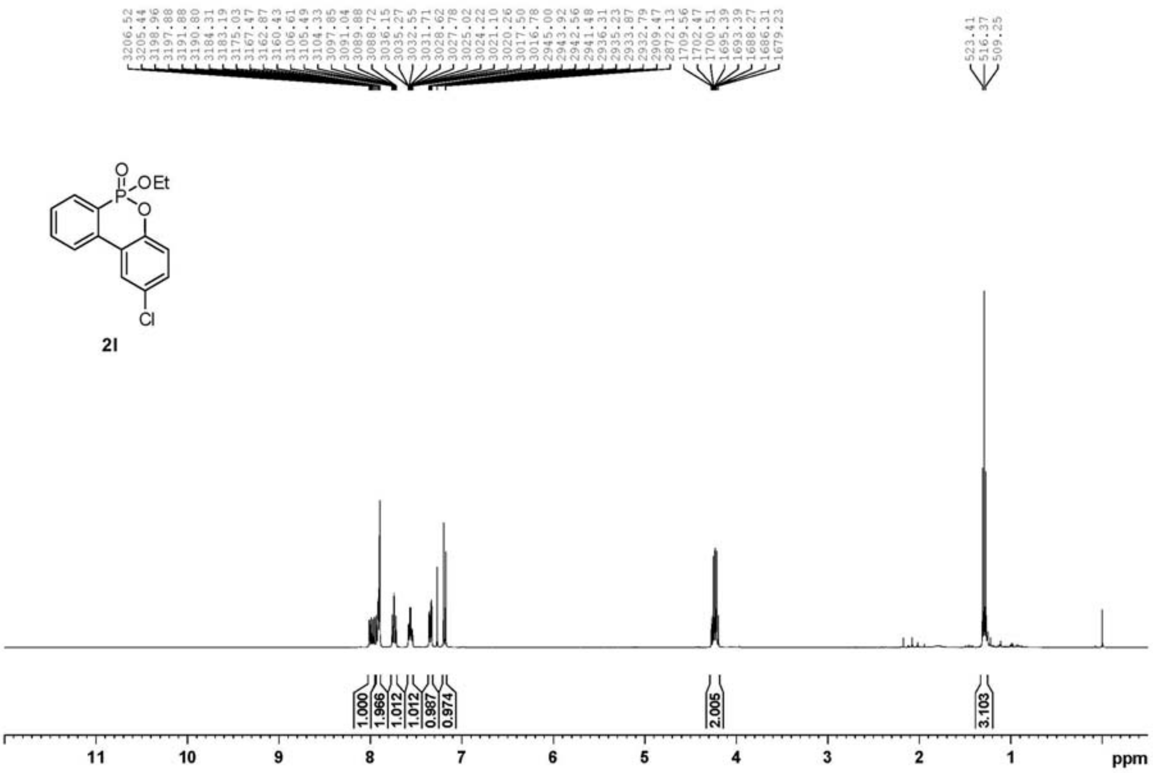
3206.52
3205.44
3198.96
3197.88
3191.88
3190.80
3184.31
3183.19
3175.03
3167.47
3162.87
3160.43
3106.61
3105.49
3104.33
3097.85
3091.04
3089.88
3088.72
3036.15
3035.27
3032.55
3031.71
3028.62
3027.78
3025.02
3024.22
3021.10
3020.26
3017.50
3016.78
2945.00
2943.92
2942.56
2941.48
2936.31
2935.23
2933.87
2932.79
2909.47
2872.13
1709.56
1702.47
1700.51
1695.39
1693.39
1688.27
1686.31
1679.23
523.41
516.37
509.25
O
OEt
P
O
Cl
2l
1.000
1.966
1.012
1.012
0.987
0.974
2.005
3.103
11 10 9 8 7 6 5 4 3 2 1 ppm

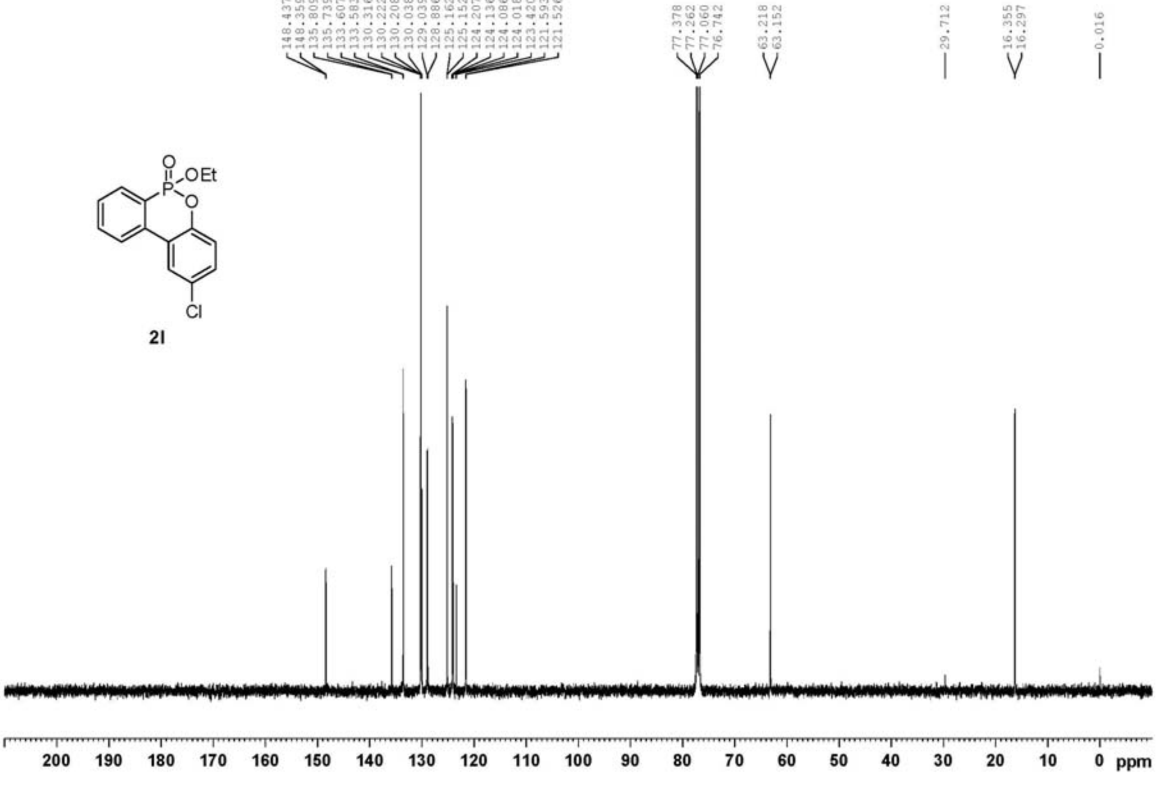
148.437
148.359
135.809
135.739
133.607
133.583
130.316
130.222
130.208
130.038
129.039
128.886
125.162
125.152
124.207
124.136
124.086
124.018
123.420
121.593
121.526
77.378
77.262
77.060
76.742
63.218
63.152
29.712
16.355
16.297
0.016
O
OEt
P
O
Cl
2l
200 190 180 170 160 150 140 130 120 110 100 90 80 70 60 50 40 30 20 10 0 ppm

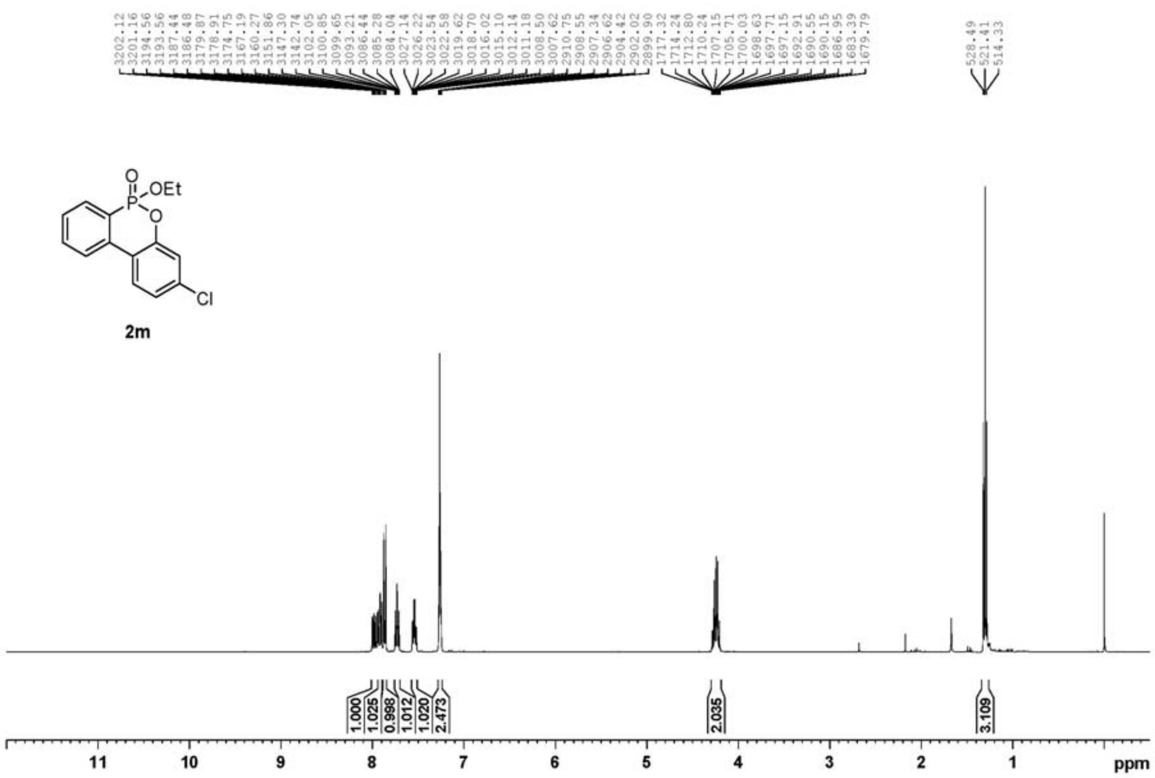

3202.12
3201.16
3194.56
3193.56
3187.44
3186.48
3179.91
3179.87
3174.75
3167.19
3160.27
3151.86
3147.30
3142.74
3102.05
3100.85
3099.65
3093.21
3086.44
3085.28
3084.04
3027.14
3026.22
3023.54
3022.58
3019.62
3018.70
3016.02
3015.10
3012.14
3011.18
3008.50
3007.62
2910.75
2908.55
2907.34
2906.62
2904.42
2902.02
2899.90
1717.32
1714.24
1712.80
1710.24
1707.15
1705.71
1700.03
1698.63
1697.71
1697.15
1692.91
1690.55
1686.95
1683.39
1679.79
528.49
521.41
514.33
OEt
O
P
O
Cl
2m
1.000
1.025
0.998
1.012
1.020
2.473
2.035
3.109
11
10
9
8
7
6
5
4
3
2
1
ppm

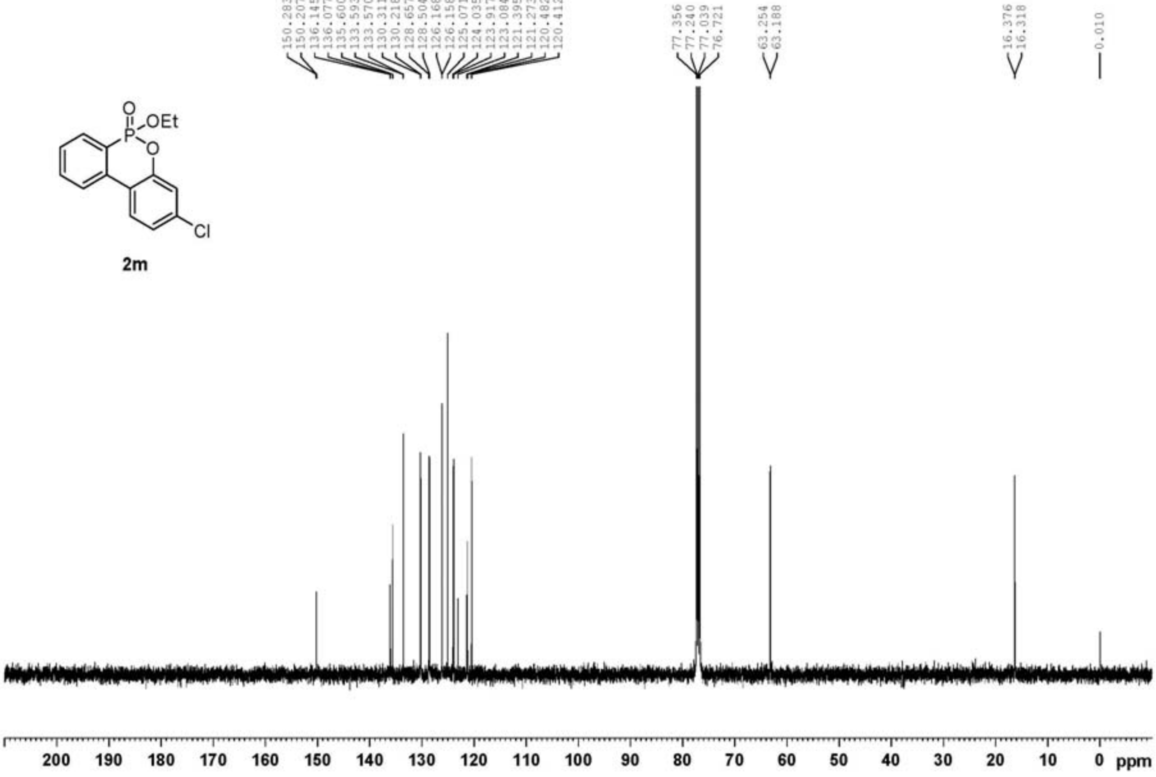

150.283
150.207
136.145
136.077
135.600
133.593
133.570
130.311
130.218
128.657
128.504
126.168
126.158
125.071
124.035
123.917
123.084
121.395
121.273
120.482
120.412
77.356
77.240
77.039
76.721
63.254
63.188
16.376
16.318
0.010
OEt
O
P
O
Cl
2m
200
190
180
170
160
150
140
130
120
110
100
90
80
70
60
50
40
30
20
10
0
ppm

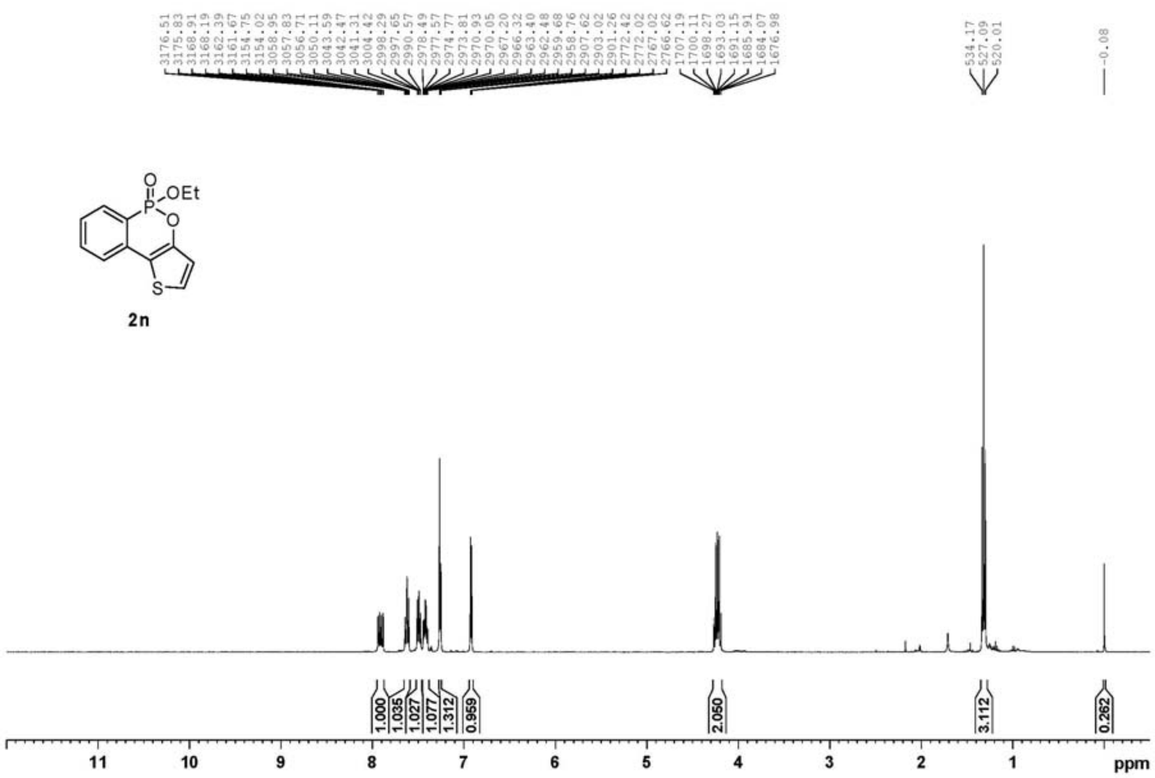
2n
ppm

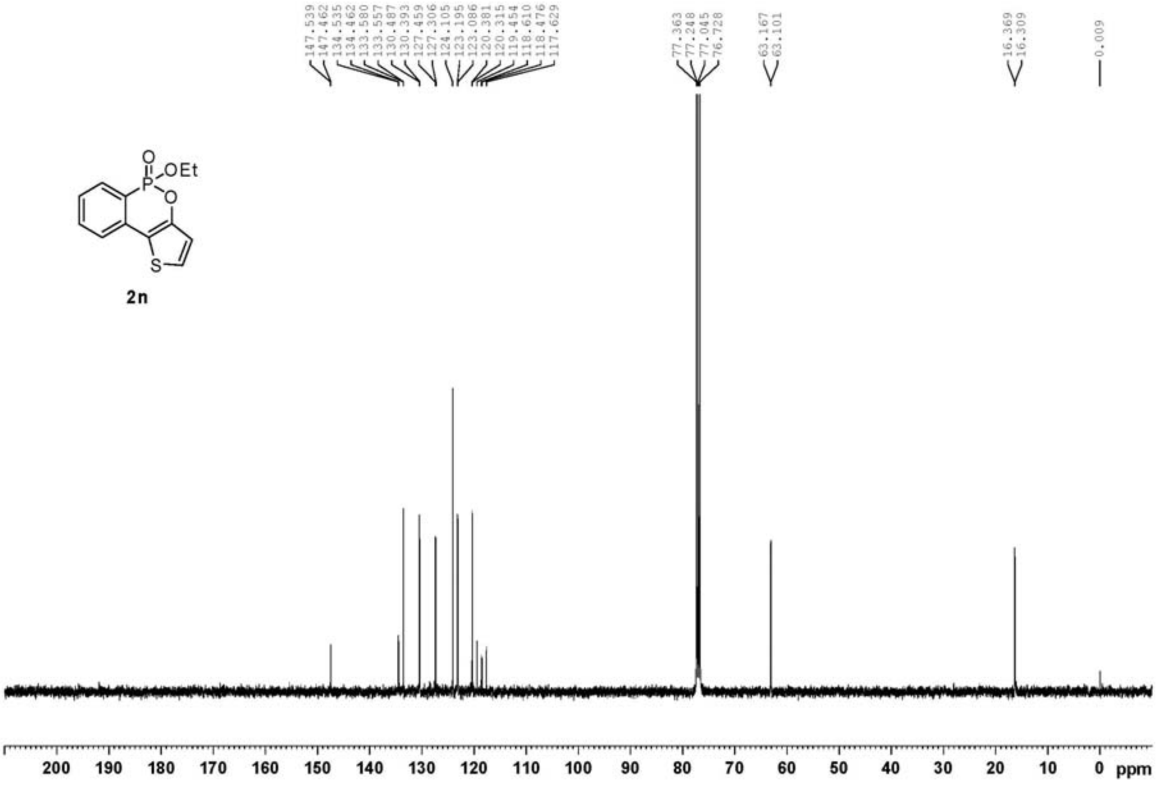
2n
ppm

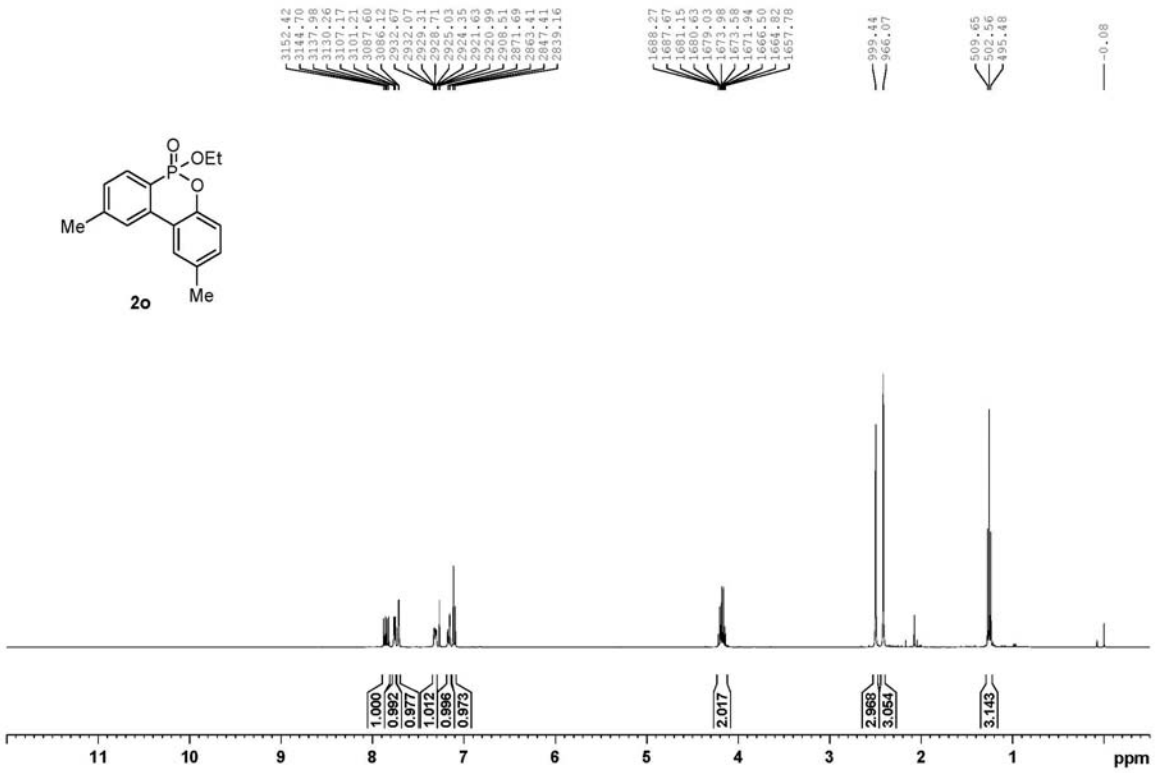
3152.42
3144.70
3137.98
3130.26
3107.17
3101.21
3087.60
3086.12
2932.67
2932.07
2929.31
2928.71
2925.03
2924.35
2921.63
2920.99
2908.51
2871.69
2863.41
2847.41
2839.16
1688.27
1687.67
1681.15
1680.63
1679.03
1673.98
1673.58
1671.94
1666.50
1664.82
1657.78
999.44
966.07
509.65
502.56
495.48
-0.08
OEt
O
P
O
Me
Me
2o
1.000
0.992
0.977
1.012
0.996
0.973
2.017
2.968
3.054
3.143
11
10
9
8
7
6
5
4
3
2
1
ppm

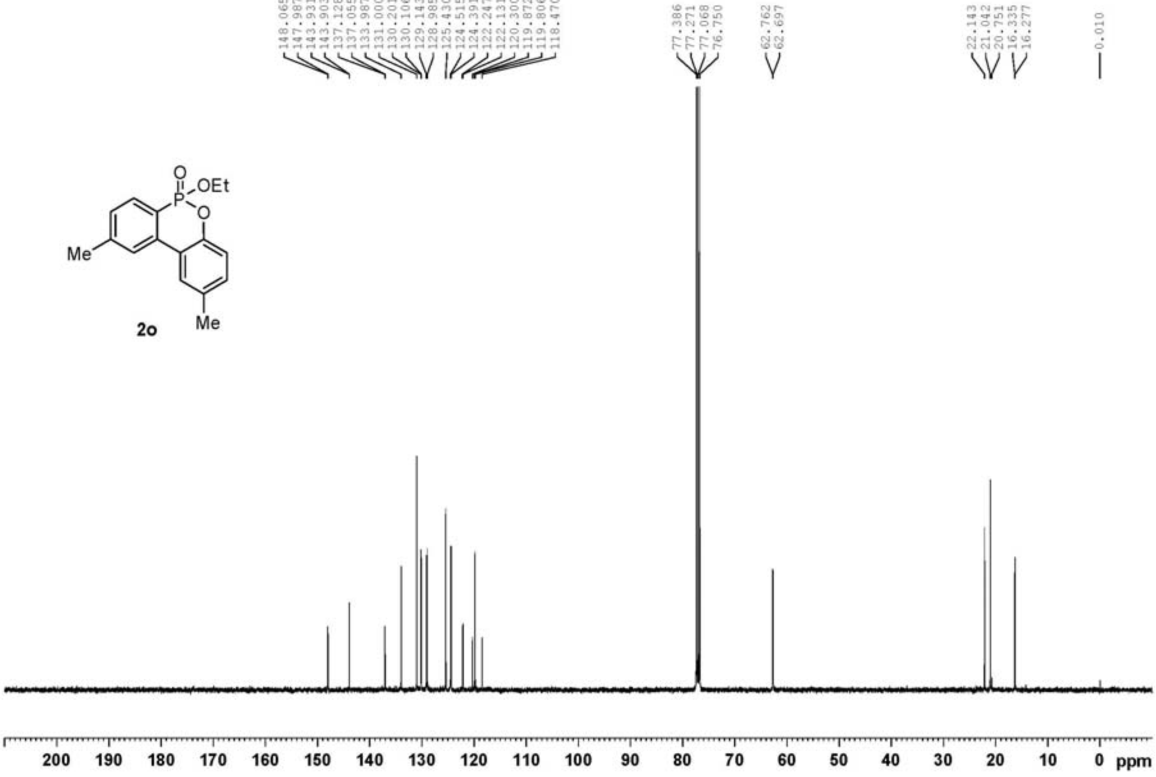
148.065
147.987
143.931
143.903
137.128
137.055
133.987
131.000
130.201
130.106
129.143
128.985
125.430
124.515
124.391
122.247
122.131
120.300
119.872
119.806
118.470
77.386
77.271
77.068
76.750
62.762
62.697
22.143
21.042
20.751
16.335
16.277
0.010
OEt
O
P
O
Me
Me
2o
200
190
180
170
160
150
140
130
120
110
100
90
80
70
60
50
40
30
20
10
0
ppm

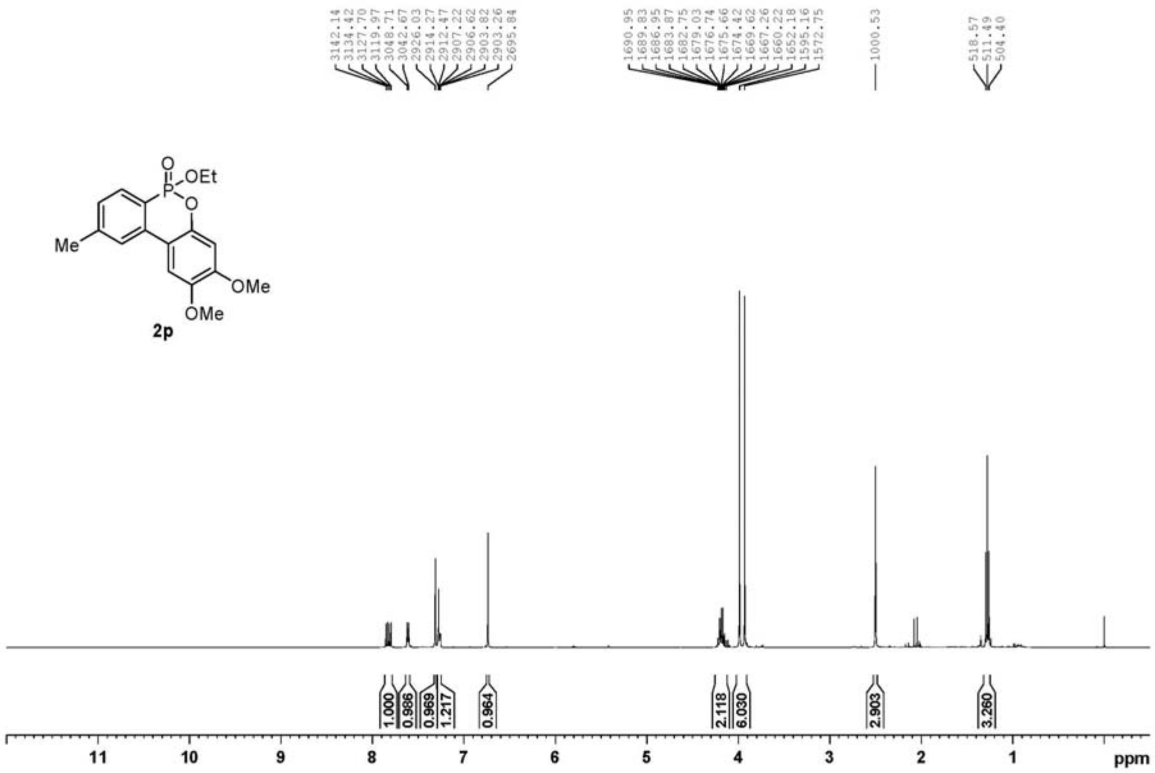

3142.14
3134.42
3127.70
3119.97
3048.71
3042.67
2926.03
2914.27
2912.47
2907.22
2906.62
2903.82
2903.26
2695.84
1690.95
1689.83
1686.95
1683.87
1682.75
1679.03
1676.74
1675.66
1674.42
1669.62
1667.26
1660.22
1652.18
1595.16
1572.75
1000.53
518.57
511.49
504.40
OEt
O
P
O
Me
OMe
OMe
2p
1.000
0.986
0.969
1.217
0.964
2.118
6.030
2.903
3.260
11 10 9 8 7 6 5 4 3 2 1 ppm

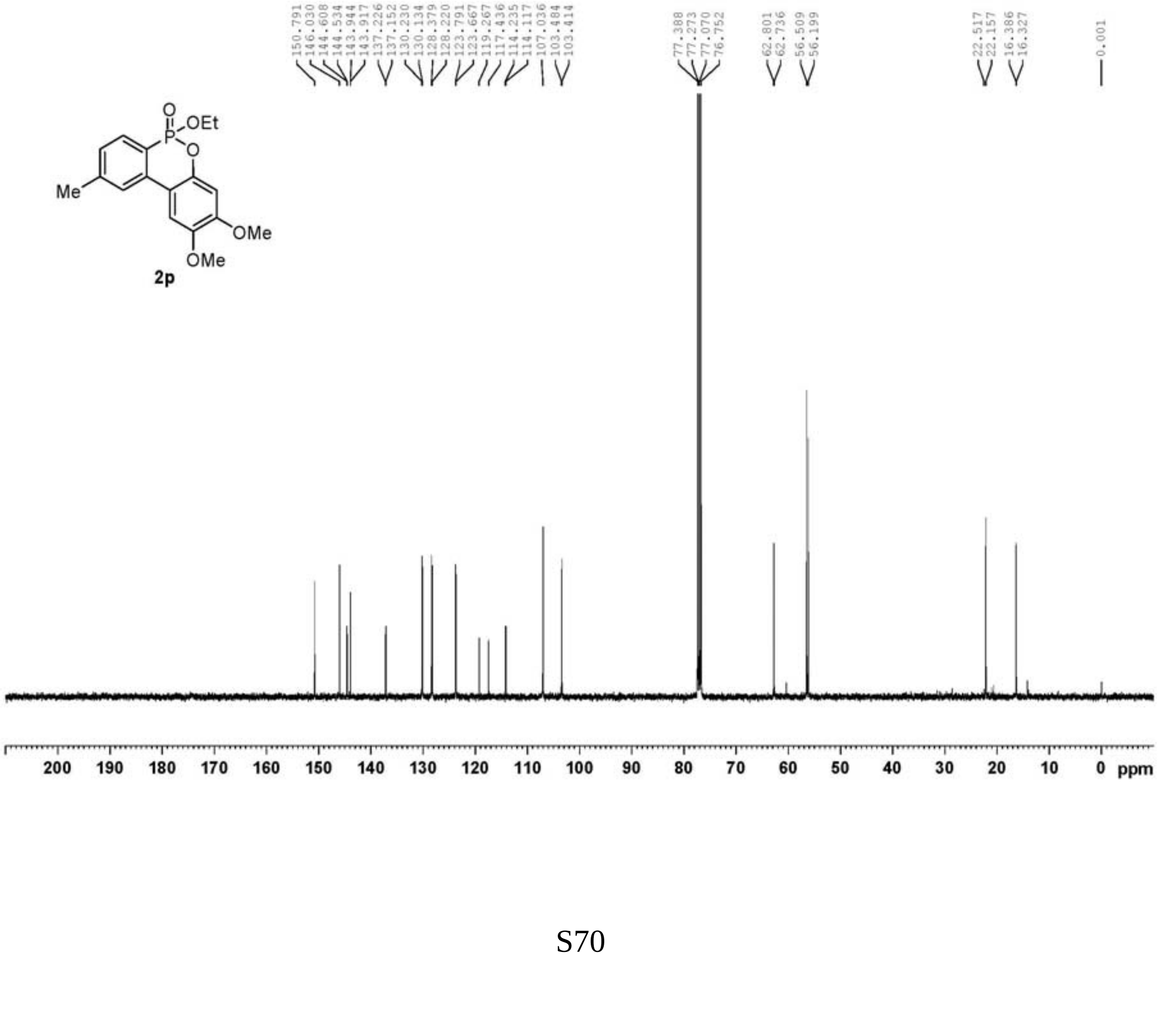

150.791
146.030
144.608
144.534
143.944
143.917
137.226
137.152
130.230
130.134
128.379
128.220
123.791
123.667
119.267
117.436
114.235
114.117
107.036
103.484
103.414
77.388
77.273
77.070
76.752
62.801
62.736
56.509
56.199
22.517
22.157
16.386
16.327
0.001
OEt
O
P
O
Me
OMe
OMe
2p
200 190 180 170 160 150 140 130 120 110 100 90 80 70 60 50 40 30 20 10 0 ppm

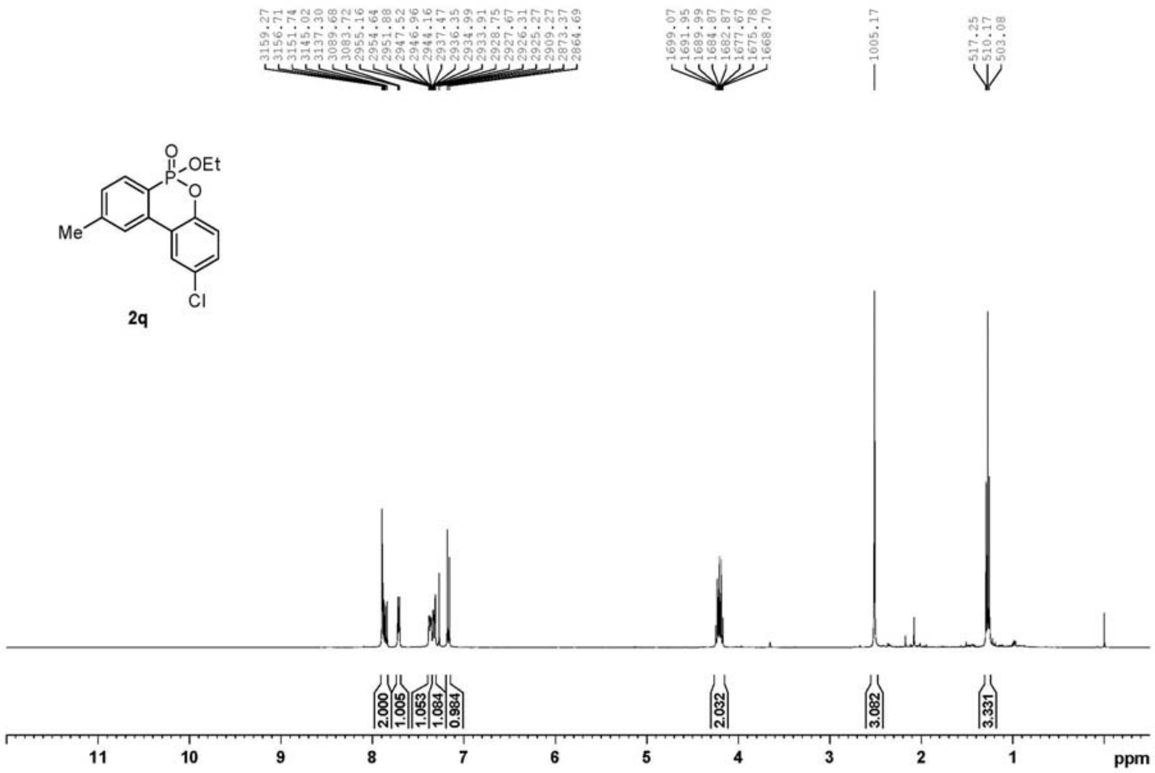
3159.27
3156.71
3151.74
3145.02
3137.30
3089.68
3083.72
2955.16
2954.64
2951.88
2947.52
2946.96
2944.16
2937.47
2936.35
2934.99
2933.91
2928.75
2927.67
2926.31
2925.27
2909.27
2873.37
2864.69
1699.07
1691.95
1689.99
1684.87
1682.87
1677.67
1675.78
1668.70
1005.17
517.25
510.17
503.08
OEt
Me
Cl
2q
2.000
1.005
1.053
1.084
0.984
2.032
3.082
3.331
11 10 9 8 7 6 5 4 3 2 1 ppm

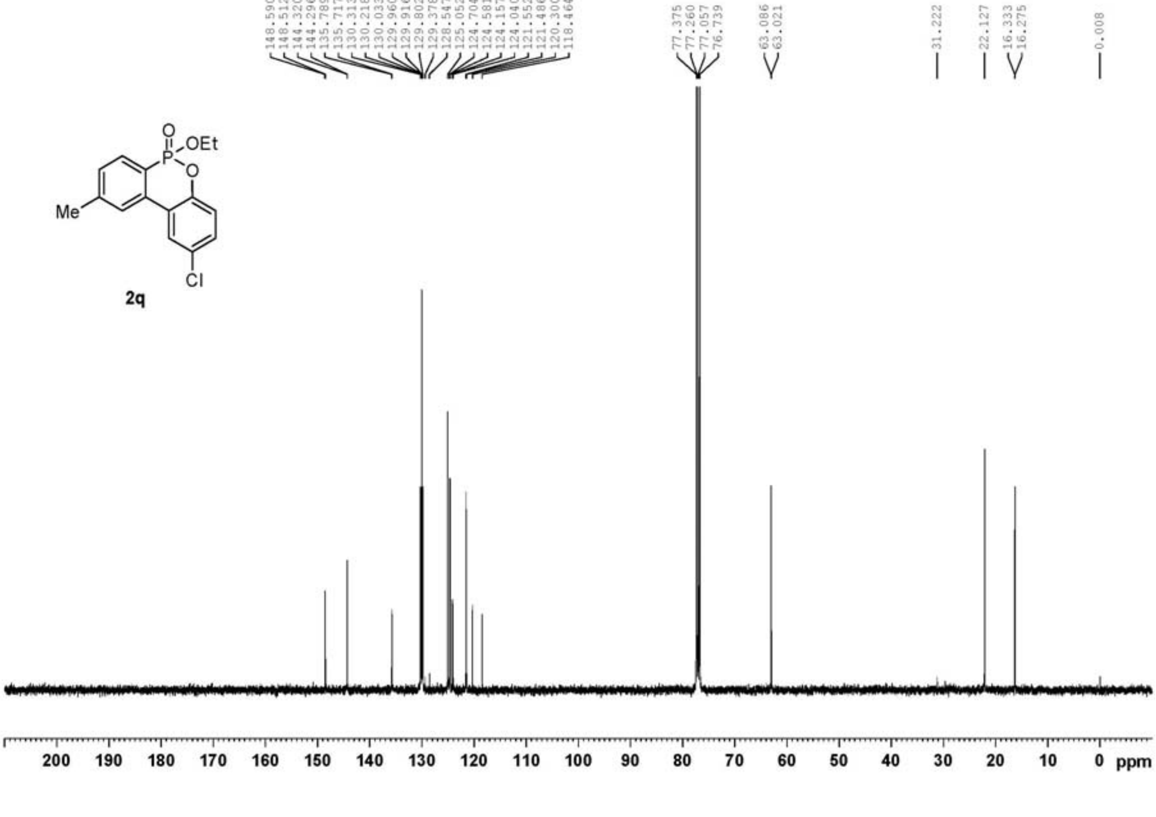
148.590
148.512
144.320
144.296
135.789
135.717
130.313
130.218
130.033
129.960
129.916
129.802
129.378
128.547
125.052
124.704
124.581
124.157
124.040
121.552
121.486
120.300
118.464
77.375
77.260
77.057
76.739
63.086
63.021
31.222
22.127
16.333
16.275
0.008
OEt
Me
Cl
2q
200 190 180 170 160 150 140 130 120 110 100 90 80 70 60 50 40 30 20 10 0 ppm

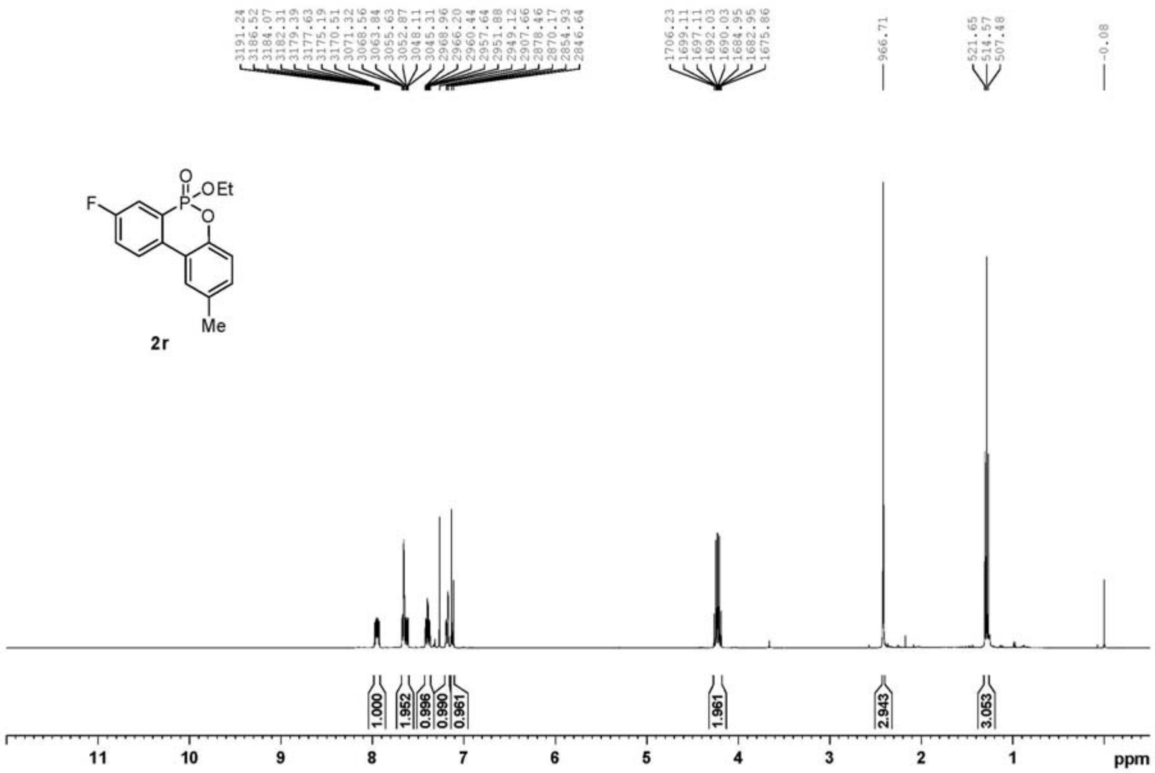
3191.24
3186.52
3184.07
3182.31
3179.39
3177.63
3175.19
3170.51
3071.32
3068.56
3063.84
3055.63
3052.87
3048.11
3045.31
2968.96
2966.20
2960.44
2957.64
2951.88
2949.12
2907.66
2878.46
2870.17
2854.93
2846.64
1706.23
1699.11
1697.11
1692.03
1690.03
1684.95
1682.95
1675.86
966.71
521.65
514.57
507.48
-0.08
F
O
P
OEt
O
Me
2r
1.000
1.952
0.996
0.990
0.961
1.961
2.943
3.053
11
10
9
8
7
6
5
4
3
2
1
ppm

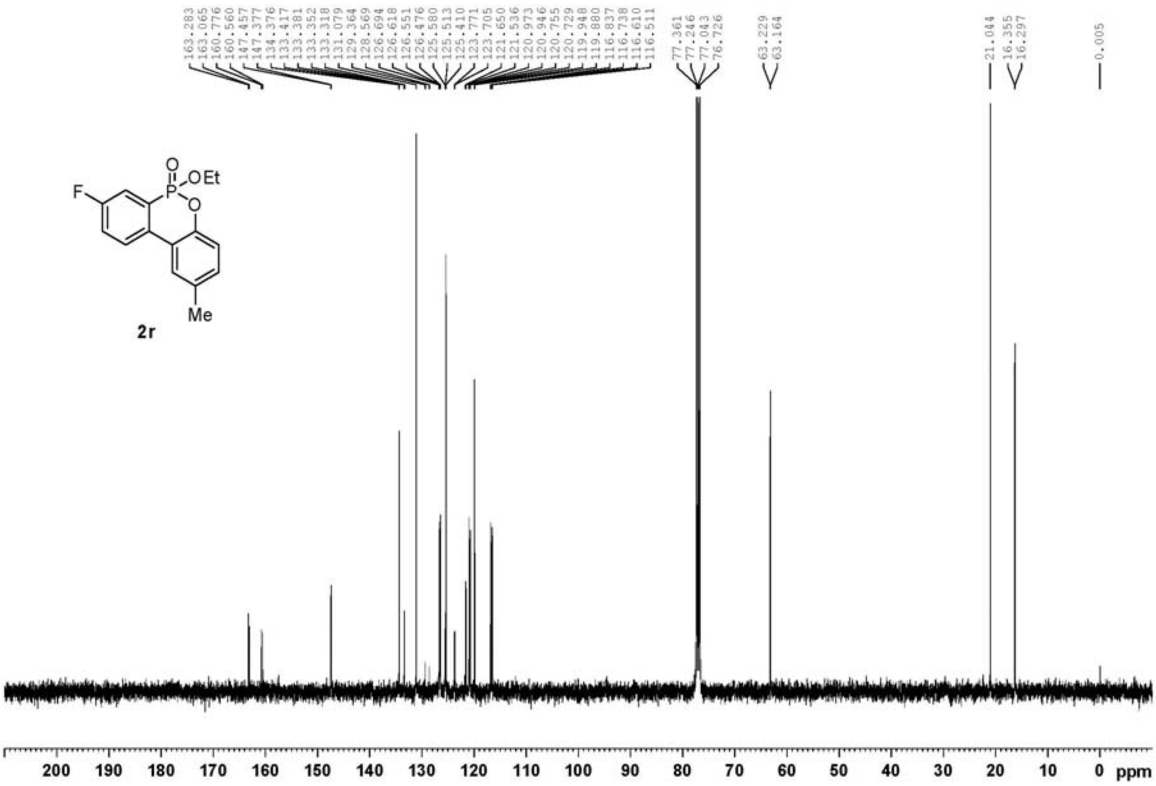
163.283
163.065
160.776
160.560
147.457
147.377
134.376
133.417
133.381
133.352
133.318
131.079
129.364
128.569
126.694
126.618
126.551
126.476
125.580
125.513
125.410
123.771
123.705
121.650
121.536
120.973
120.946
120.755
120.729
119.948
119.880
116.837
116.738
116.610
116.511
77.361
77.246
77.043
76.726
63.229
63.164
21.044
16.355
16.297
0.005
F
O
P
OEt
O
Me
2r
200
190
180
170
160
150
140
130
120
110
100
90
80
70
60
50
40
30
20
10
0
ppm

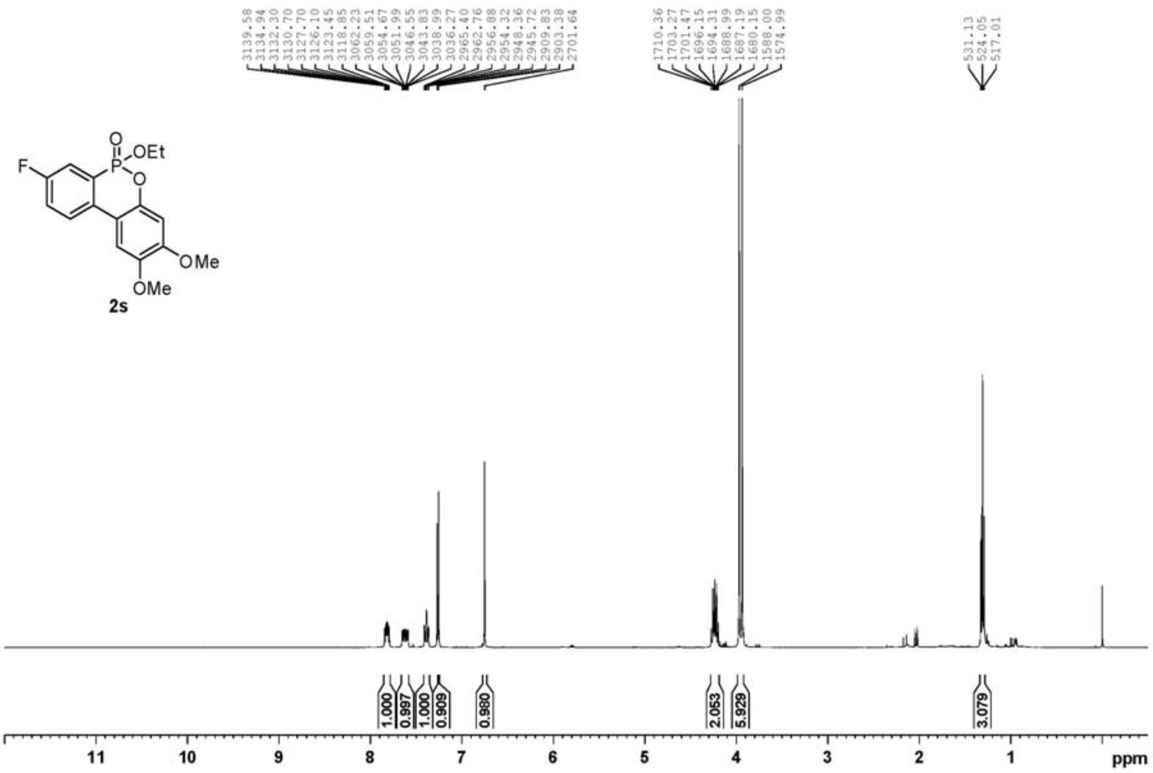
F
OEt
O
P
OMe
OMe
2s
ppm

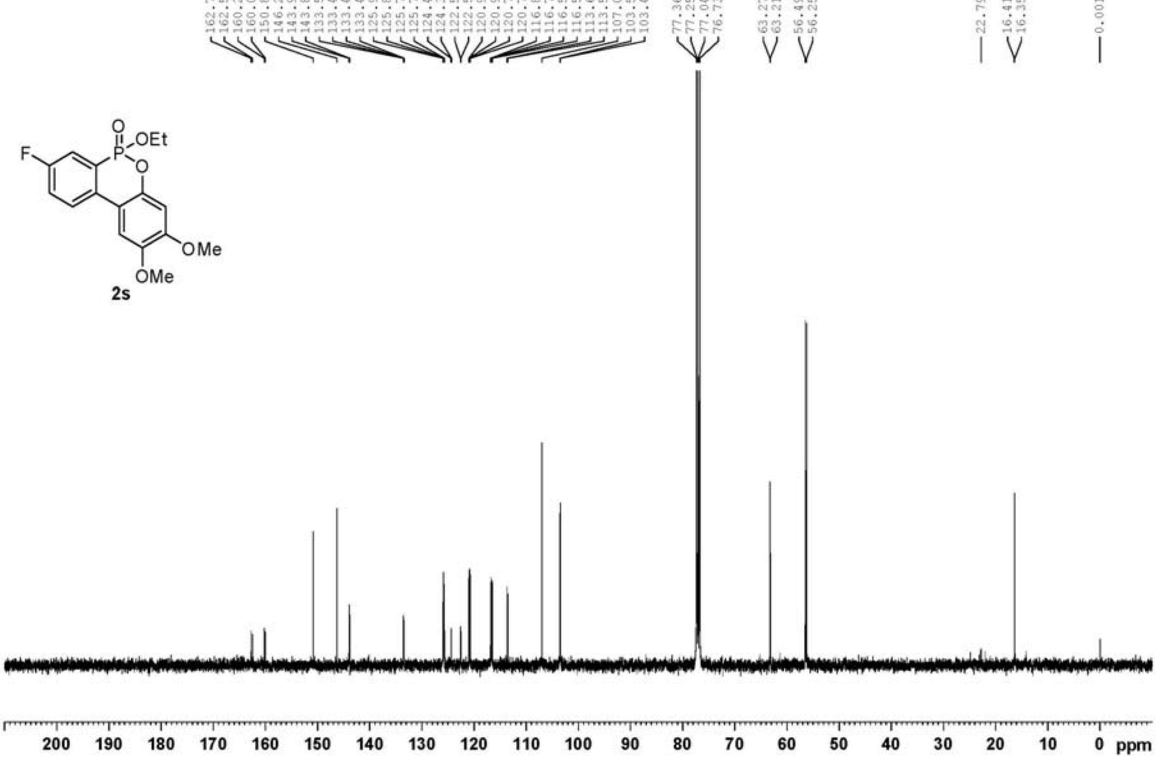
F
OEt
O
P
OMe
OMe
2s
ppm

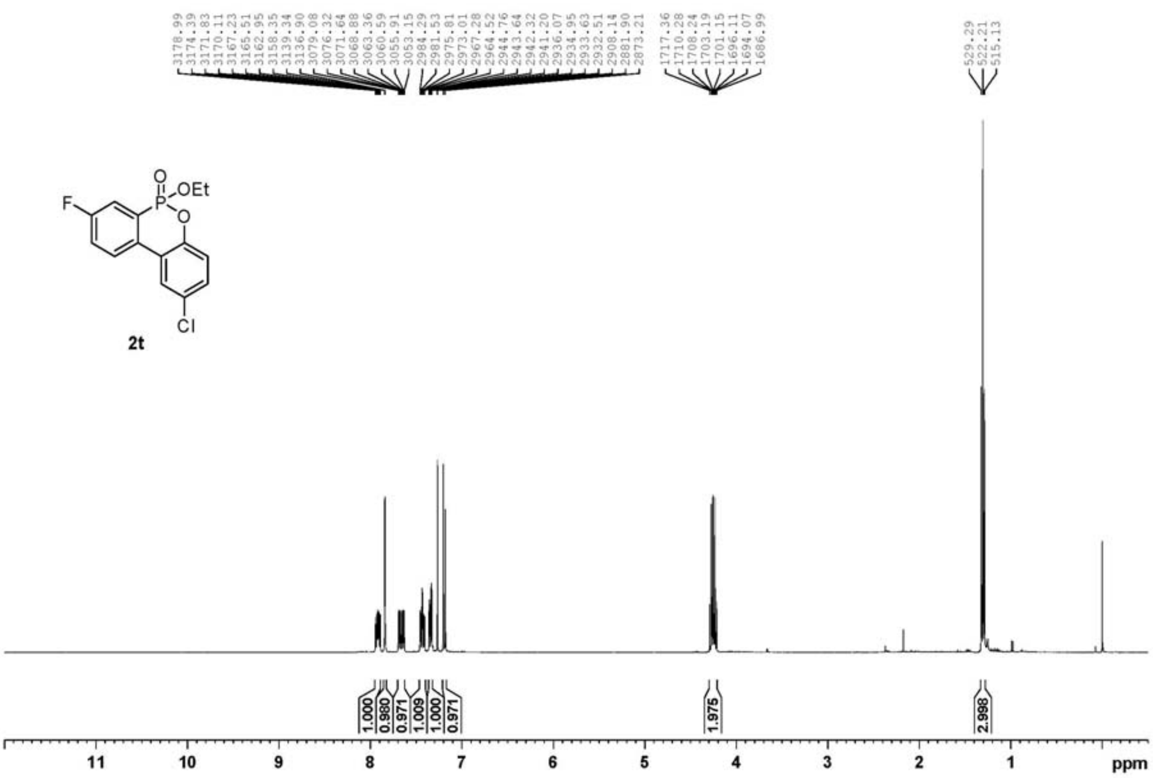
3178.99
3174.39
3171.83
3170.11
3167.23
3165.51
3162.95
3158.35
3139.34
3136.90
3079.08
3076.32
3071.64
3068.88
3063.36
3060.59
3055.91
3053.15
2984.29
2981.53
2975.81
2973.01
2967.28
2964.52
2944.76
2943.64
2942.32
2941.20
2936.07
2934.95
2933.63
2932.51
2908.14
2881.90
2873.21
1717.36
1710.28
1708.24
1703.19
1701.15
1696.11
1694.07
1686.99
529.29
522.21
515.13
F
O
P
OEt
O
Cl
2t
1.000
0.980
0.971
1.009
1.000
0.971
1.975
2.998
11 10 9 8 7 6 5 4 3 2 1 ppm

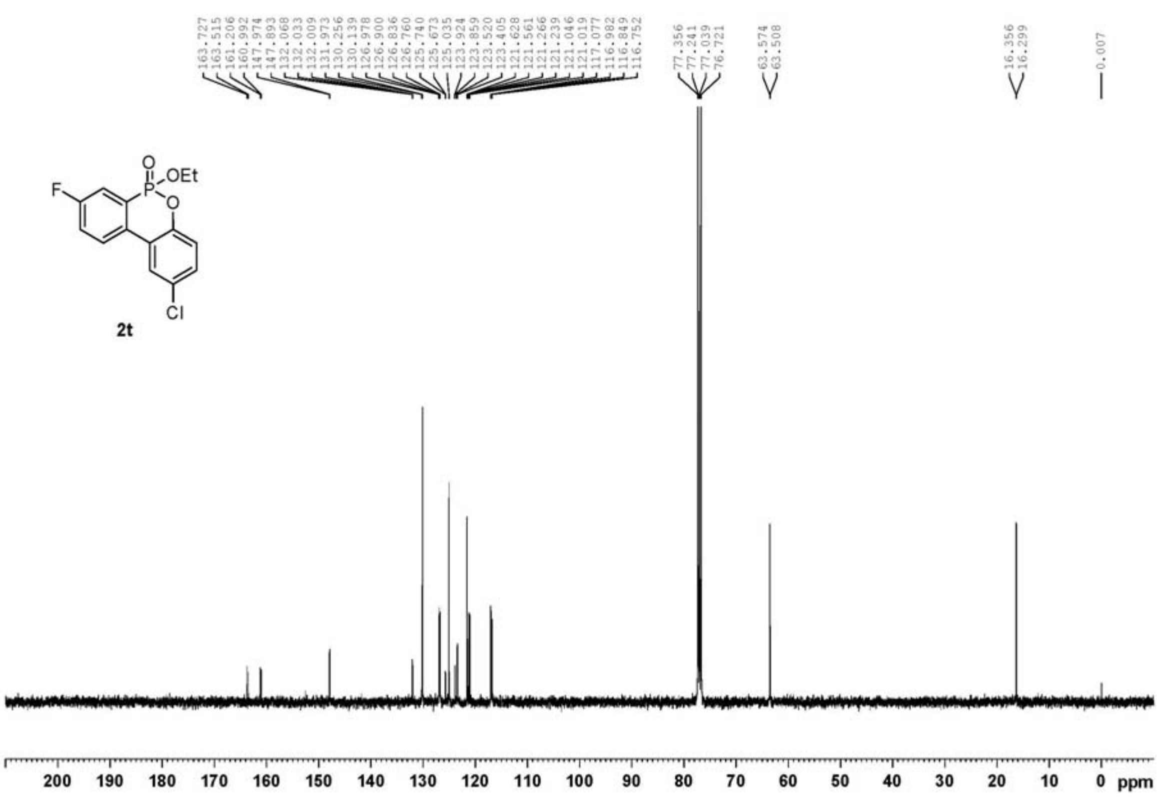
163.727
163.515
161.206
160.992
147.974
147.893
132.068
132.033
132.009
131.973
130.256
130.139
126.978
126.900
126.836
126.760
125.740
125.673
125.035
123.924
123.859
123.520
123.405
121.628
121.561
121.266
121.239
121.046
121.019
117.077
116.982
116.849
116.752
77.356
77.241
77.039
76.721
63.574
63.508
16.356
16.299
0.007
F
O
P
OEt
O
Cl
2t
200 190 180 170 160 150 140 130 120 110 100 90 80 70 60 50 40 30 20 10 0 ppm

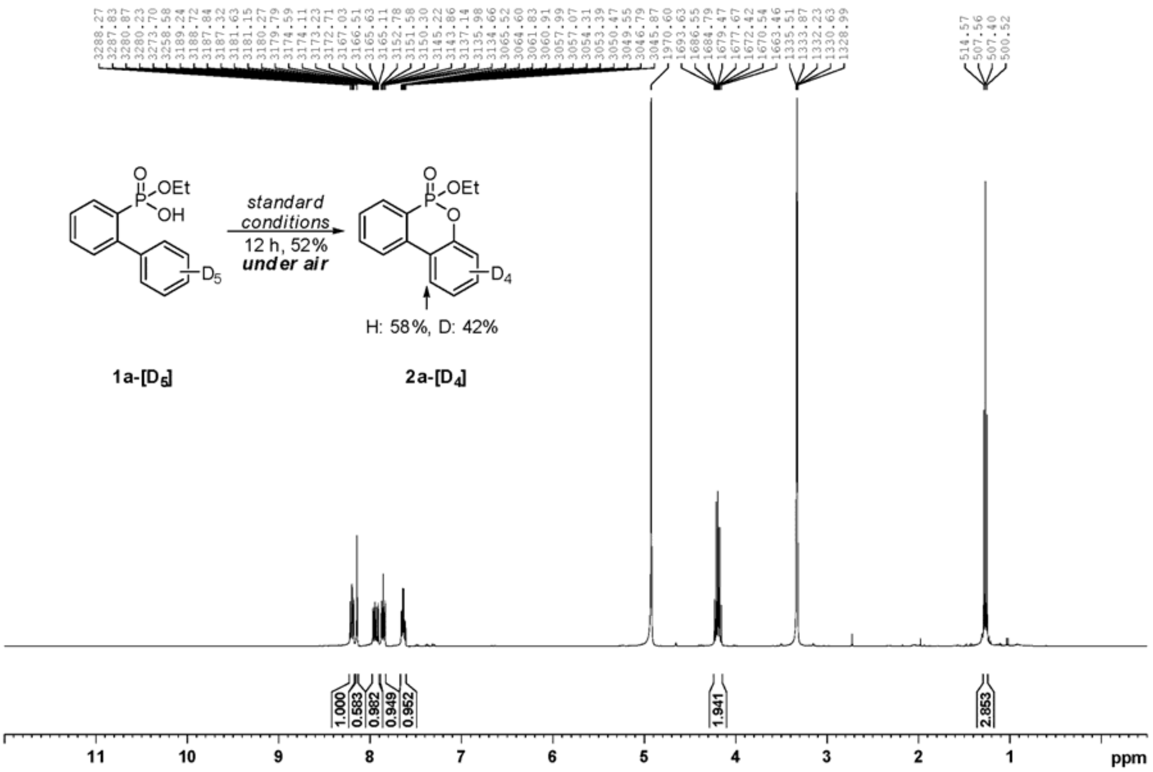
OEt
OH
standard conditions
12 h, 52%
under air
OEt
D5
D4
H: 58%, D: 42%
1a-[D5]
2a-[D4]
ppm

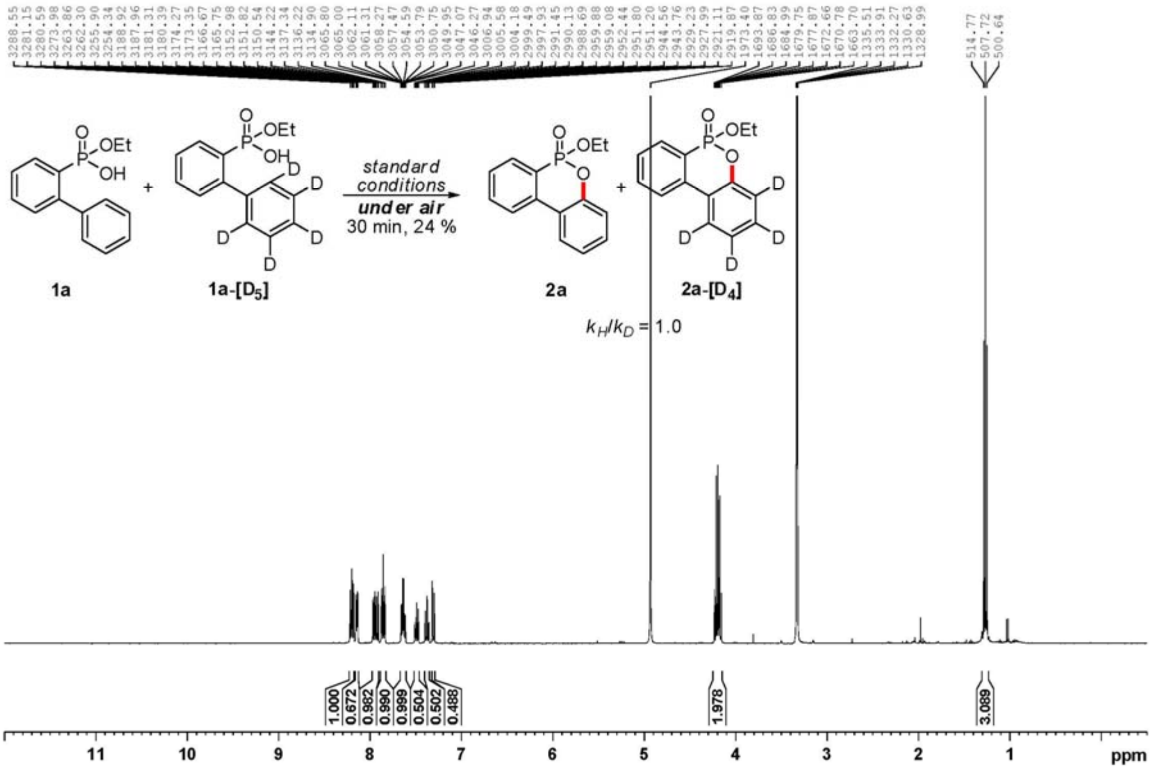
OEt
OH
1a
1a-[D5]
standard conditions
under air
30 min, 24 %
2a
2a-[D4]
kH/kD = 1.0
ppm

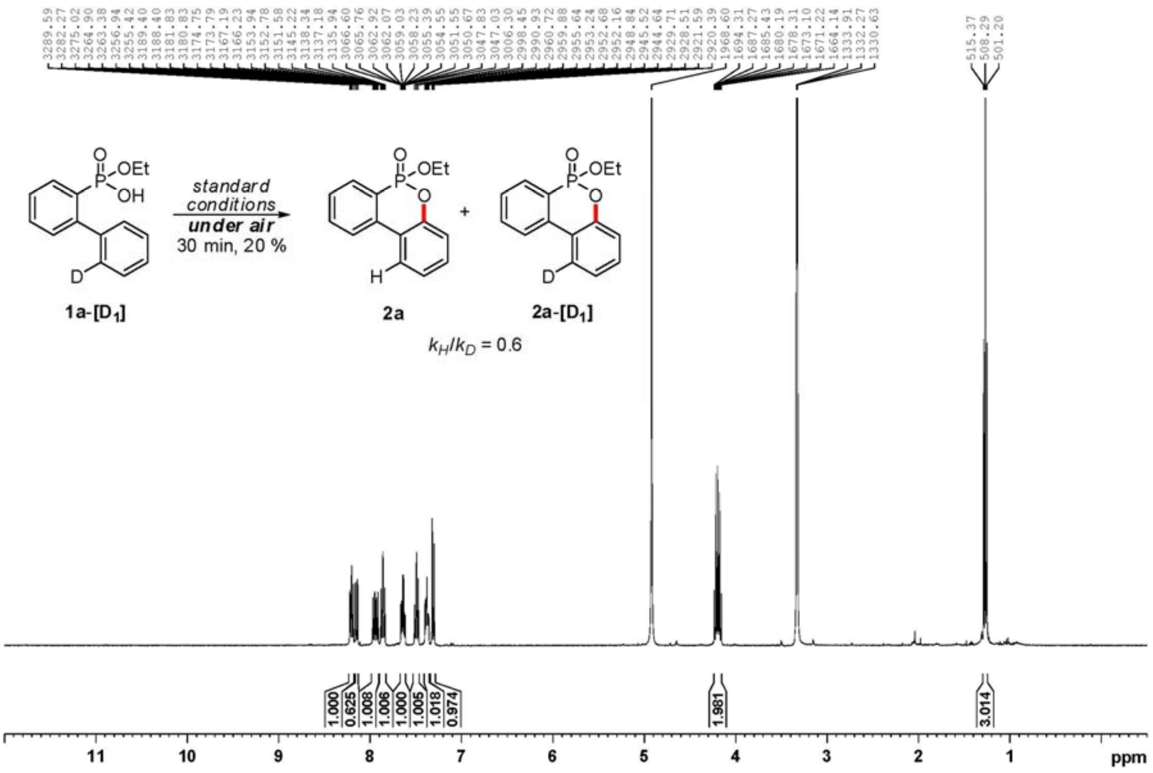

standard
conditions
under air
30 min, 20 %
1a-[D1]
2a
2a-[D1]
kH/kD = 0.6
ppm
